# Supplementary material for: Nuclear cGAS restricts L1 retrotransposition by promoting TRIM41-mediated ORF2p ubiquitination and degradation
Source: Nat Commun. 2023 Dec 12;14:8217. doi: 10.1038/s41467-023-43001-y (PMC10716122; doi:10.1038/s41467-023-43001-y)
Supplement: Supplementary file 1 — Supplementary Information [file 41467_2023_43001_MOESM1_ESM.pdf]

Supplementary Information

Nuclear cGAS restricts L1 retrotransposition by promoting TRIM41-mediated ORF2p ubiquitination and degradation

Zhengyi Zhen, Yu Chen, Haiyan Wang, Huanyin Tang, Haiping Zhang, Haipeng Liu, Ying Jiang, Zhiyong Mao[*]

These authors contributed equally: Zhengyi Zhen, Yu Chen.

[*]Corresponding author. Email: zhiyong_mao@tongji.edu.cn; Tel: +86-21-65978166; Fax: +86-21-65981041.

This file contains:

- Supplementary Figure 1-7
- Supplementary Table 1-2

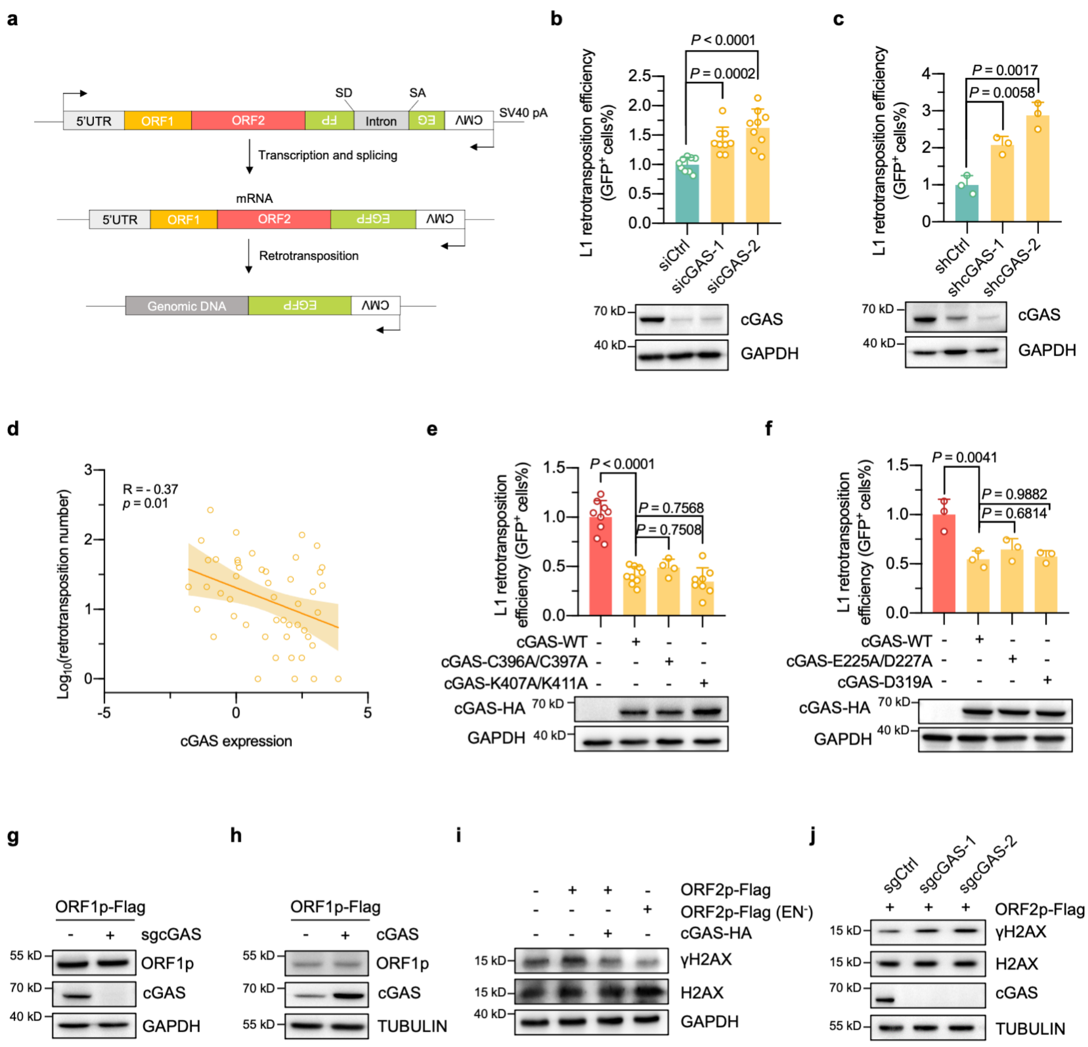

Supplementary Fig. 1 cGAS inhibits L1 retrotransposition independently on its DNA binding ability and canonical enzyme activity.

a, Schematic diagram showing the L1 retrotransposition efficiency reporter pEGFP-LRE3. **b,** Analysis of L1 retrotransposition efficiency in cGAS-knockdown HeLa cells. The retrotransposition efficiency is shown in the top, and the expression of cGAS analyzed by Western blot is shown in the bottom. n = 9 independent experiments. **c,** Analysis of L1 retrotransposition efficiency in HeLa cells with chromosome-integrated shRNA against cGAS. n = 3 independent experiments. **d,** Analysis of the correlation between cGAS mRNA levels and L1 retrotransposition events in lung squamous cell carcinoma (SCC). **e,** Comparison of the effect of overexpression of WT cGAS and its DNA-binding mutants (C396A/C397A and K407A/K411A) on L1 retrotransposition efficiency in HeLa cells. n = at least 4 independent experiments. **f,** Comparison of the effect of overexpression of WT cGAS and its enzyme-dead mutants (E225A/D227A and D319A) on L1 retrotransposition efficiency in HeLa cells. n = 3 independent experiments. **g,** The effect of cGAS knockout on ORF1p protein levels in HeLa cells as determined by Western blotting. **h,** Analysis of the effect of cGAS overexpression on ORF1p protein levels in HeLa cells. **i,** Analysis of the influence of cGAS overexpression on the level of γH2AX induced by ORF2p endonuclease. HEK293T cells were transfected with a vector encoding ORF2p-Flag WT or EN mutant and/or cGAS-HA. Whole-cell lysates were prepared for Western blot analysis using an antibody against γH2AX. **j,** Analysis of the influence of cGAS knockout on the level of γH2AX induced by ORF2p in cGAS-knockout cells. Data are presented as mean values ± s.d. for **b, c, e, f**. Student's t test for **b**, **c**, **e** and **f**. All experiments were repeated at least three times.

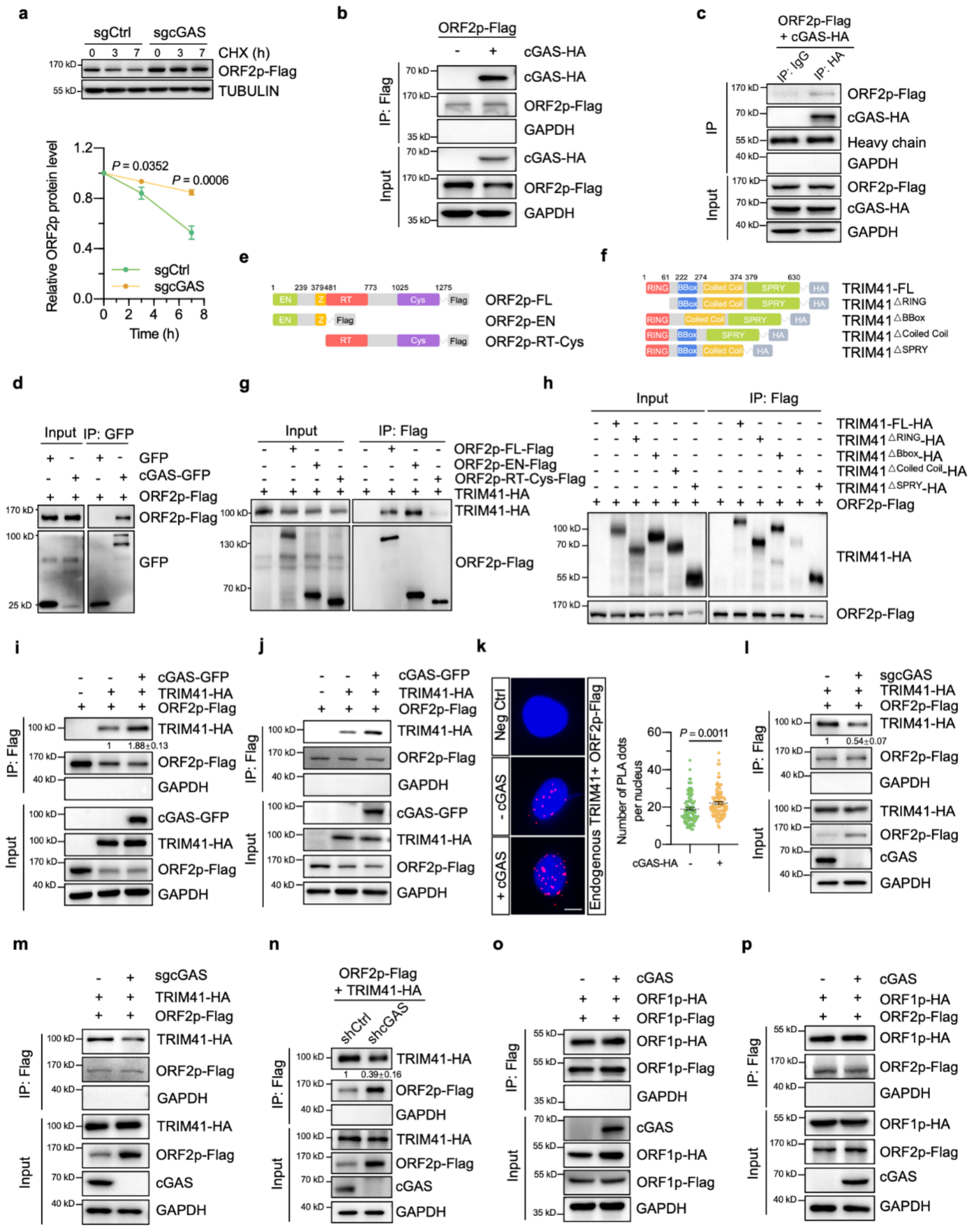

Supplementary Fig. 2 cGAS interacts with ORF2p, and promotes the interaction between TRIM41 and ORF2p.

a, Analysis of ORF2p protein levels in cGAS-knockout and control cells treated with CHX. Data are presented as mean values ± s.d. **b, c,** Reciprocal co-IP analysis of the interaction between cGAS and ORF2p in HEK293T cells. Cells were transfected with the indicated plasmids and harvested for use in co-IP experiments with beads coupled with antibodies against Flag or HA 24 h post-transfection. **d,** Analysis of the interaction between cGAS and ORF2p *in vitro* with purified proteins. **e,** Schematic representation of ORF2p truncated mutants used in this study. **f,** Schematic representation of TRIM41 truncated mutants used in this study. **g,** Identification of the domains of ORF2p mediating its interaction with TRIM41 by co-IP in HEK293T cells. **h,** Identification of the domains of TRIM41 mediating its interaction with ORF2p in HEK293T cells as determined by co-IP. **i, j,** Analysis of the effect of cGAS overexpression on the TRIM41-ORF2p interaction by using both the HRP-conjugated antibody and fluorescent antibody. **k,** Analysis of the effect of cGAS overexpression on the TRIM41-ORF2p interaction in HeLa cells with using PLA assay (scale bar: 10 μm). n > 50 cells per group. Data are presented as mean values ± s.e.m.. **l, m,** Analysis of the effect of cGAS knockout on the TRIM41-ORF2p interaction by using the HRP-conjugated antibody and fluorescent antibody. **n,** Analysis of the effect of cGAS knockdown on the TRIM41-ORF2p interaction. **o,** The effect of cGAS overexpression on ORF1p trimerization analyzed by co-IP and Western blotting. **p,** Analysis of the effect of cGAS overexpression on the ORF1p-ORF2p interaction. Student's t test for **a**, and the Mann–Whitney U test for **k**. All the Inputs and IPs were from the same experiments. All experiments were repeated at least three times.

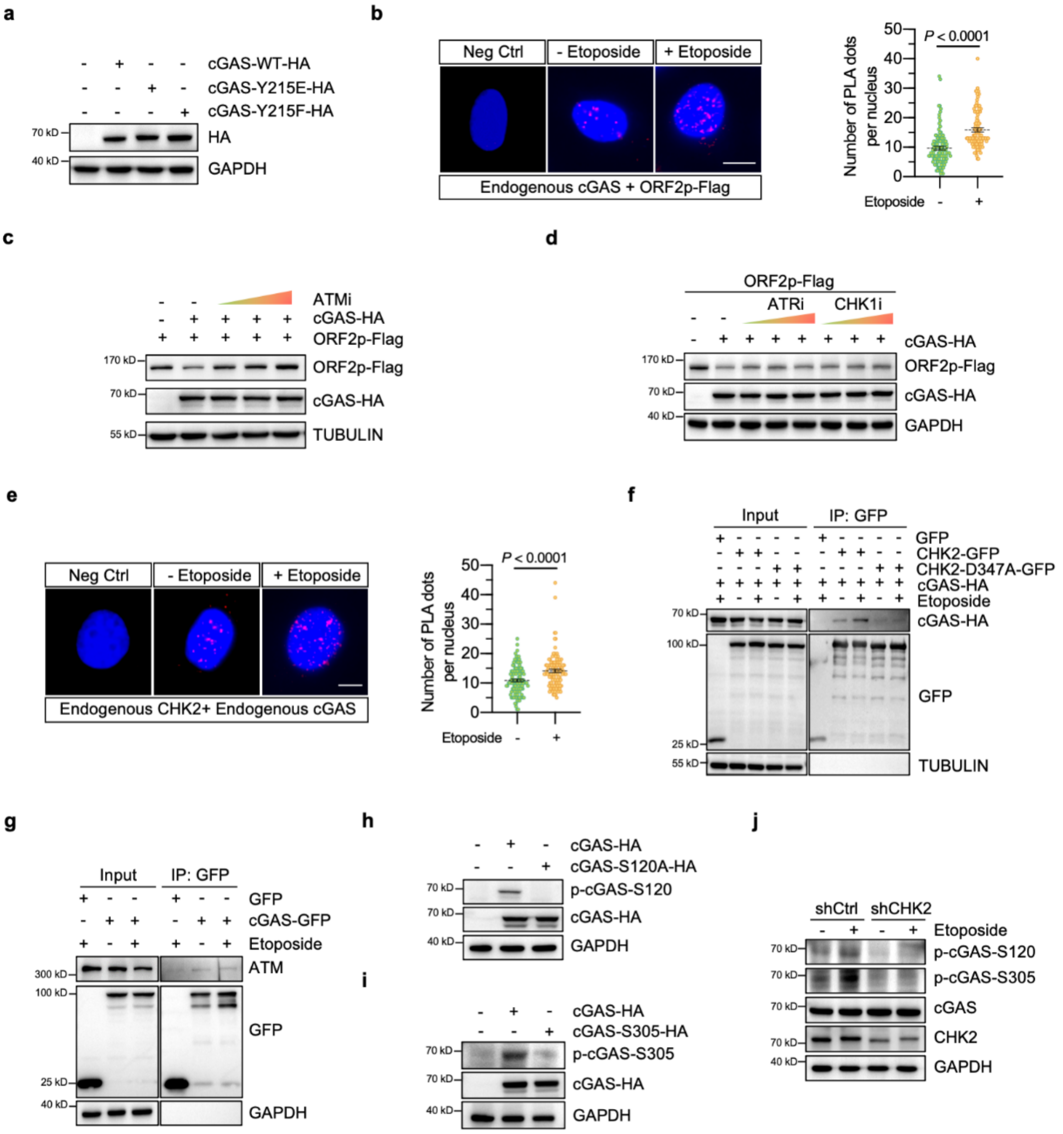

Supplementary Fig. 3 CHK2 kinase regulates cGAS$^{S120/S305}$ phosphorylation in response to DNA damage.

a, Western blot analysis of the expression of HA-tagged cGAS and its mutants. **b,** Analysis of the association between cGAS and ORF2p in HeLa cells post the occurrence of DNA damage using PLA assay (scale bar: 10 µm). n > 50 cells per group. Data are presented as mean values ± s.e.m.. **c,** The effect of an ATM inhibitor on cGAS-mediated repression of ORF2p protein levels in HeLa cells. **d,** The effect of ATR and CHK1 inhibitors on cGAS-mediated repression of ORF2p protein levels in HeLa cells. **e,** Analysis of the association between cGAS and CHK2 in HeLa cells post the occurrence of DNA damage using PLA assay (scale bar: 10 µm). n > 50 cells per group. Data are presented as mean values ± s.e.m.. **f,** Comparison of the interaction between cGAS and CHK2-WT or CHK2-D347A using fluorescent antibody after DNA was damaged. Cells were treated with 100 µg/mL etoposide for 4 h before being harvested for use in co-IP and Western blot analysis. **g,** Co-IP analysis of the interaction between cGAS and ATM upon after DNA was damaged in HEK293T cells. **h,** Validation of the antibody against phosphorylated cGAS at S120 residue. **i,** Validation of the antibody against phosphorylated cGAS at S305 residue. **j,** The effect of CHK2 depletion on cGAS phosphorylation levels at the S120 or S305 residues after DNA was damaged. Cells were treated with 100 µg/mL etoposide and lysed for Western blot analysis 30 min post-etoposide exposure. The Mann–Whitney U test was performed for **b** and **e**. All the Inputs and IPs were from the same experiments. All experiments were repeated at least three times.

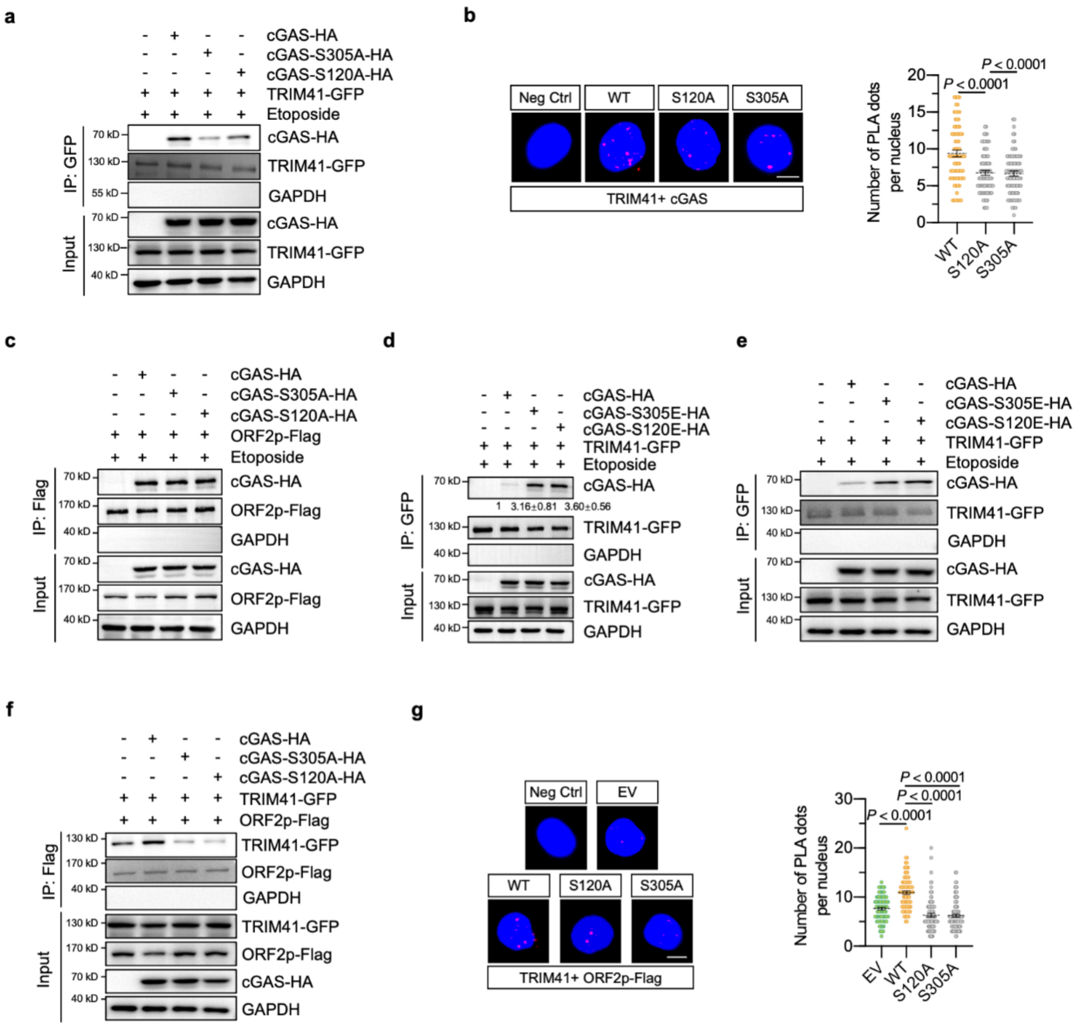

Supplementary Fig. 4 CHK2 phosphorylates cGAS at S120/S305 residues to enhance the association between cGAS-TRIM41 and TRIM41-ORF2p.

a, Comparison of the interaction between TRIM41 and cGAS or its mutants after DNA was damaged using the fluorescent antibody. Cells were transfected with the indicated plasmids and treated with 100 μg/mL etoposide for 4 h before being harvested at 24 h post-transfection. **b,** Comparison of the interaction between TRIM41 and cGAS or its mutants after DNA was damaged by PLA assay (scale bar: 10 μm). n > 50 cells per group. Data are presented as mean values ± s.e.m. **c,** Comparison of the interaction between ORF2p and cGAS or its mutants after DNA was damaged. **d, e,** Comparison of the interaction between TRIM41 and cGAS or its mutants after DNA was damaged, by using both the HRP-conjugated antibody and fluorescent antibody. **f,** Analysis of the effect of overexpressing cGAS or its mutants on the TRIM41-ORF2p interaction in HEK293T cells by using the fluorescent antibody. **g,** Analysis of the effect of overexpressing cGAS or its mutants on the TRIM41-ORF2p interaction in HeLa cells by using PLA assay (scale bar: 10 μm). n > 50 cells per group. Data are presented as mean values ± s.e.m. Mann–Whitney U test for **b** and **g.** All the Inputs and IPs were from the same experiments. All experiments were repeated at least three times.

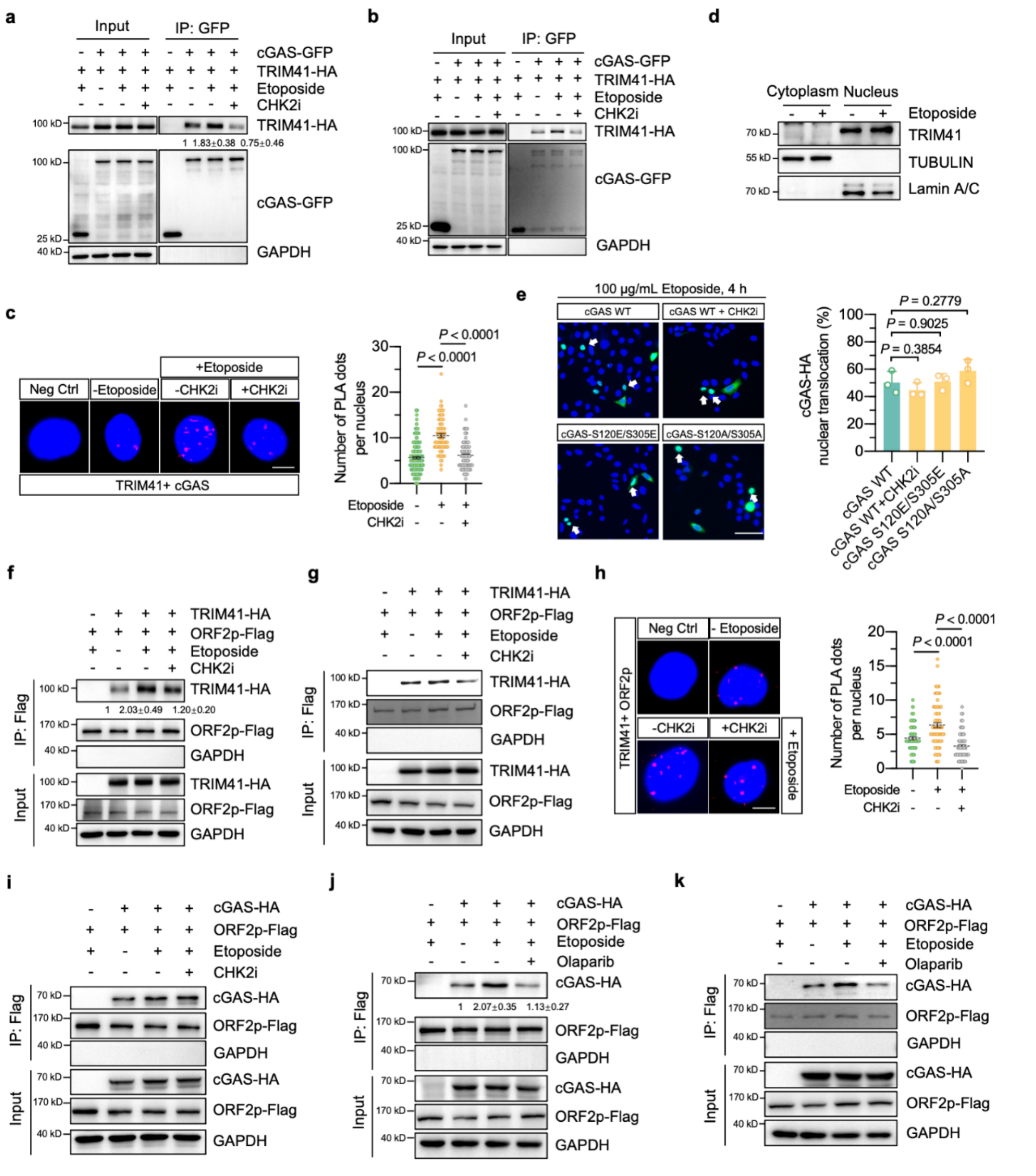

Supplementary Fig. 5 CHK2 mediates the enhanced association between cGAS-TRIM41 and TRIM41-ORF2p upon the occurrence of DNA damage.

a, b, Analysis of the effect of a CHK2 inhibitor on the interaction between cGAS and TRIM41 after DNA was damaged. Cells were treated with 10 µM CHK2 inhibitor for 6 h, followed by treatment with 100 µg/mL etoposide for 4 h before being harvested for use in co-IP and Western blot analysis by using both the HRP-conjugated antibody and fluorescent antibody. **c,** Analysis of the effect of a CHK2 inhibitor on cGAS-TRIM41 interaction using PLA assay after DNA was damaged. (scale bar: 10 µm). n > 50 cells per group. **d,** Analysis of the subcellular localization of TRIM41 in HEK293 cells after DNA was damaged. **e,** Analysis of the nuclear localization of cGAS or its mutants after DNA was damaged (scale bar: 100 µm). n > 50 cells examined over 3 independent experiments per group. **f, g,** Analysis of the effect of a CHK2 inhibitor on the interaction between TRIM41 and ORF2p after DNA was damaged, by using both the HRP-conjugated antibody and fluorescent antibody. **h,** Analysis of the effect of a CHK2 inhibitor on the association of TRIM41-ORF2p after DNA was damaged in HeLa cells using PLA assay (scale bar: 10 µm). n > 50 cells per group. **i,** Analysis of the effect of the CHK2 inhibitor on the interaction between cGAS and ORF2p after DNA was damaged. **j, k,** PARPi disrupted the increase in the cGAS-ORF2p interaction after DNA was damaged by using both the HRP-conjugated antibody and fluorescent antibody. Cells were treated with 5 µM olaparib for 6 h post-transfection, and then treated with 100 µg/mL etoposide for 4 h before being harvested. Data are presented as mean values ± s.e.m. for **c** and **h**, and are presented as mean values ± s.d. for **e**. Mann–Whitney U test for **c** and **h**, and Student's t test for **e**. All the Inputs and IPs were from the same experiments. All experiments were repeated at least three times.

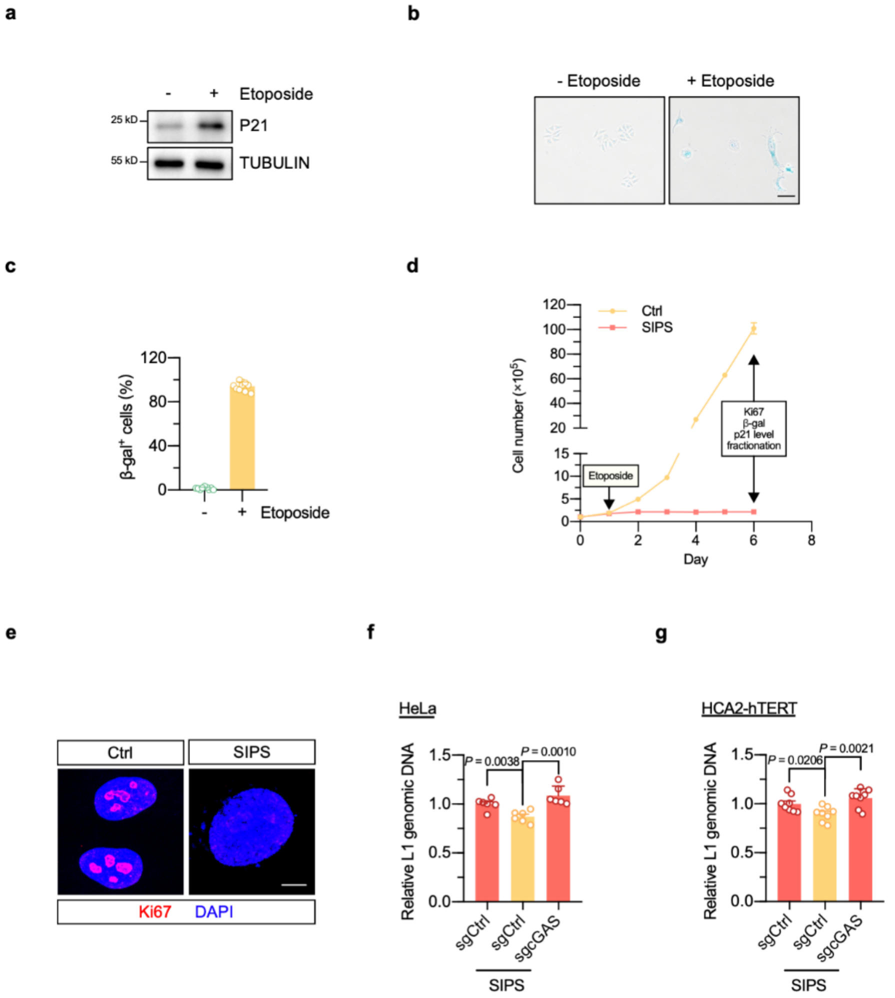

Supplementary Fig. 6 L1 retrotransposition is repressed in SIPS cells.

a, Analysis of the p21 protein level, a well-documented senescence marker, in HeLa cells treated with or without etoposide. **b, c,** SA-β-gal staining of HeLa cells treated with or without etoposide. Representative images are shown in **b** (scale bar: 100 μm), and the percentage of SA-β-gal-positive cells is shown in **c**. **d,** The growth curve of HeLa cells treated with or without etoposide. HeLa cells (1×10^5) were seeded on Day 0 and treated with etoposide on Day 1. Cells were counted every 24 h from day 1 to 6. Data are presented as mean values ± s.d. **e,** Ki67 staining of HeLa cells treated with or without etoposide (scale bar: 10 μm). **f,** Analysis of endogenous L1 copy number in control or cGAS-knockout senescent HeLa cells. n = 6 independent experiments. **g,** Analysis of endogenous L1 copy number in control or cGAS-knockout senescent HCA2-hTERT cells. Data are presented as mean values ± s.d.. n = 8 independent experiments. Student's t test for **f** and **g**. All experiments were repeated at least three times.

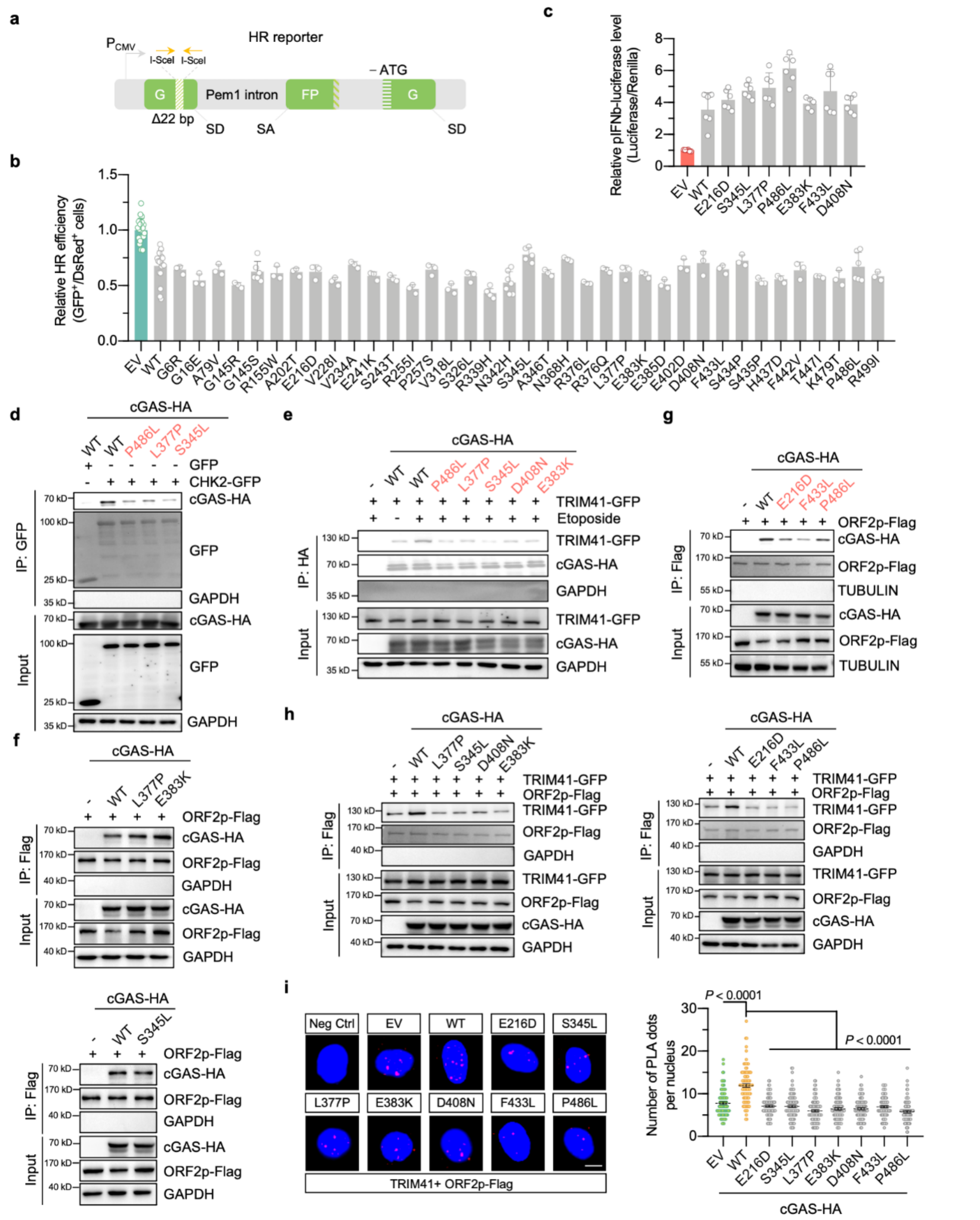

Supplementary Fig. 7 Cancer-associated cGAS mutations abolish the repressive effect of cGAS on L1 retrotransposition by disrupting the CHK2-cGAS-TRIM41-ORF2p regulatory axis.

a, Schematic diagram showing the HR efficiency reporter. **b,** The effect of cGAS overexpression and its cancer-associated mutants on HR efficiency. n = at least 3 independent experiments. **c,** The effect of cancer-associated cGAS mutants on IFN promoter activity. A total of 3×10^5 HEK293T cells with STING stably integrated into the genome were transfected with 6 µg of pIFN-luciferase vector and 20 ng of Renilla luciferase vector and then lysed for use in a luciferase activity analysis 24 h post-transfection. n = at least 5 independent experiments. **d,** Analysis of the interaction between CHK2 and WT or mutated cGAS using the fluorescent antibody. Cells were transfected with the indicated plasmids and collected for co-IP experiments 24 h post-transfection. **e,** Analysis of the association between TRIM41 and WT or mutated cGAS after DNA was damaged using the fluorescent antibody. The cells were transfected with the indicated plasmids and treated with 100 µg/mL etoposide for 90 min before being harvested for use in co-IP experiment. **f, g,** Analysis of the interaction between ORF2p and WT or mutated cGAS by using both the HRP-conjugated antibody and fluorescent antibody. **h,** Analysis of the effect of overexpressing WT or mutated cGAS on the TRIM41-ORF2p interaction using the fluorescent antibody. **i,** Analysis of the effect of overexpressing WT or mutated cGAS on the TRIM41-ORF2p interaction using the PLA assay (scale bar: 10 µm). n > 50 cells per group. Data are presented as mean values ± s.e.m.. Mann–Whitney U test for **i**. All the Inputs and IPs were from the same experiments. All experiments were repeated at least three times.

Supplementary Tables

Supplementary Table 1. The qPCR primers used in this study.

Category	Sequences	Application
qL1-F<br>qL1-R	CAAACACCGCATATTCTCACTCA<br>CTTCCTGTGTCCATGTGATCTCA	RT-qPCR for analyzing L1 mRNA level
qGAPDH-F<br>qGAPDH-R	ATGACATCAAGAAGGTGGTG<br>CATACCAGGAAATGAGCTTG	Internal control for RT-qPCR
ghL1-ORF2-F<br>ghL1-ORF2-R	TGCGGAGAAATAGGAACACTTTT<br>TGAGGAATCGCCACACTGACT	RT-qPCR for analyzing L1 copy number in human cells
gh5S-F<br>gh5S-R	CTCGTCTGATCTCGGAAGCTAAG<br>GCGGTCTCCCATCCAAGTAC	Internal control for L1 copy number analysis (human)
gmL1-ORF2-F<br>gmL1-ORF2-R	CTGGCGAGGATGTGGAGAA<br>CCTGCAATCCCACCAACAAT	RT-qPCR for analyzing L1 copy number in mice
gm5S-F<br>gm5S-R	ACGGCCATACCACCCTGAA<br>GGTCTCCCATCCAAGTACTAACCA	Internal control for L1 copy number analysis (mouse)

Supplementary Table 2. The siRNA, shRNA and sgRNA sequences used in this study.

Category	Sequences
cGAS siRNA-1	CGUGAAGAUUUCUGCACCUAA
cGAS siRNA-2	CUUUGAUAACUGCGUGACAUA
cGAS shRNA-1	CGTGAAGATTTCTGCACCTAA
cGAS shRNA-2	GGCTATCCTTCTCTCACAT
cGAS sgRNA-1	GGCCGCCCGTCCGCGCAACT
cGAS sgRNA-2	ACACTCGTGCATATTACTTT
TRIM41 sgRNA	GATTACTTCACGGACCCCG
CHK2 shRNA	GCCTTAAGACACCCGTGGC
