# Supplementary material for: Nuclear cGAS restricts L1 retrotransposition by promoting TRIM41-mediated ORF2p ubiquitination and degradation
Source: Nat Commun. 2023 Dec 12;14:8217. doi: 10.1038/s41467-023-43001-y (PMC10716122; doi:10.1038/s41467-023-43001-y)
Supplement: Supplementary file 3 — Reporting Summary [file 41467_2023_43001_MOESM3_ESM.pdf]

nature portfolio

Corresponding author(s): Zhiyong Mao

Last updated by author(s): Oct 20, 2023

Reporting Summary

Nature Portfolio wishes to improve the reproducibility of the work that we publish. This form provides structure for consistency and transparency in reporting. For further information on Nature Portfolio policies, see our Editorial Policies and the Editorial Policy Checklist.

Statistics

For all statistical analyses, confirm that the following items are present in the figure legend, table legend, main text, or Methods section.

n/a	Confirmed	
☐	☒	The exact sample size (*n*) for each experimental group/condition, given as a discrete number and unit of measurement
☐	☒	A statement on whether measurements were taken from distinct samples or whether the same sample was measured repeatedly
☐	☒	The statistical test(s) used AND whether they are one- or two-sided<br>*Only common tests should be described solely by name; describe more complex techniques in the Methods section.*
☒	☐	A description of all covariates tested
☒	☐	A description of any assumptions or corrections, such as tests of normality and adjustment for multiple comparisons
☐	☒	A full description of the statistical parameters including central tendency (e.g. means) or other basic estimates (e.g. regression coefficient) AND variation (e.g. standard deviation) or associated estimates of uncertainty (e.g. confidence intervals)
☐	☒	For null hypothesis testing, the test statistic (e.g. *F*, *t*, *r*) with confidence intervals, effect sizes, degrees of freedom and *P* value noted<br>*Give P values as exact values whenever suitable.*
☒	☐	For Bayesian analysis, information on the choice of priors and Markov chain Monte Carlo settings
☒	☐	For hierarchical and complex designs, identification of the appropriate level for tests and full reporting of outcomes
☒	☐	Estimates of effect sizes (e.g. Cohen's *d*, Pearson's *r*), indicating how they were calculated

Our web collection on statistics for biologists contains articles on many of the points above.

Software and code

Policy information about availability of computer code

Data collection	To analyze repair efficiency and retrotransposition efficiency at cellular level, cells were analyzed by flow cytometry on a BD FACSVerse. The qPCR was carried out on a ViiA 7 Real-Time PCR system (Applied Biosystems). The immunofluorescence images were taken with Nikon A1R confocal microscope.
Data analysis	Flow cytometry data was analyzed using Flowjo. Semi-quantitative analysis of western blot data was carried out by ImageJ.

For manuscripts utilizing custom algorithms or software that are central to the research but not yet described in published literature, software must be made available to editors and reviewers. We strongly encourage code deposition in a community repository (e.g. GitHub). See the Nature Portfolio guidelines for submitting code & software for further information.

Data

Policy information about availability of data

All manuscripts must include a data availability statement. This statement should provide the following information, where applicable:

- Accession codes, unique identifiers, or web links for publicly available datasets
- A description of any restrictions on data availability
- For clinical datasets or third party data, please ensure that the statement adheres to our policy

The author declare that source data for all figures are available upon request.

Research involving human participants, their data, or biological material

Policy information about studies with human participants or human data. See also policy information about sex, gender (identity/presentation), and sexual orientation and race, ethnicity and racism.

Reporting on sex and gender	No human participants and human biological materials were included in our study.
Reporting on race, ethnicity, or other socially relevant groupings	N/A
Population characteristics	N/A
Recruitment	N/A
Ethics oversight	N/A

Note that full information on the approval of the study protocol must also be provided in the manuscript.

Field-specific reporting

Please select the one below that is the best fit for your research. If you are not sure, read the appropriate sections before making your selection.

☒ Life sciences ☐ Behavioural & social sciences ☐ Ecological, evolutionary & environmental sciences

For a reference copy of the document with all sections, see nature.com/documents/nr-reporting-summary-flat.pdf

Life sciences study design

All studies must disclose on these points even when the disclosure is negative.

Sample size	Sample sizes were not predetermined. Sample sizes were generally determined based on previous studies involving similar experiments. The exact sample size (n) for each experiment was included in the related figure legend.
Data exclusions	No data were excluded from analysis.
Replication	All experimental data was reliably reproduces in multiple independent experiments as indicated in the figure legends.
Randomization	Cells were allocated randomly to each treatment group.
Blinding	The investigators were blinded during data collection and analysis where possible. This included immunofluorescence assay data collection for the quantification of gammaH2AX positive cells and β-gal positive cells.

Reporting for specific materials, systems and methods

We require information from authors about some types of materials, experimental systems and methods used in many studies. Here, indicate whether each material, system or method listed is relevant to your study. If you are not sure if a list item applies to your research, read the appropriate section before selecting a response.

Materials & experimental systems

n/a	Involved in the study	
☐	☒	Antibodies
☐	☒	Eukaryotic cell lines
☒	☐	Palaeontology and archaeology
☐	☒	Animals and other organisms
☒	☐	Clinical data
☒	☐	Dual use research of concern
☒	☐	Plants

Methods

n/a	Involved in the study	
☒	☐	ChIP-seq
☐	☒	Flow cytometry
☒	☐	MRI-based neuroimaging

Antibodies

Antibodies used	anti-Flag (MBL, Cat# M185-3L, 1/2000 for WB), Anti-Flag (Sigma–Aldrich, Cat. #F3165, 1/300 for PLA), anti-cGAS (Cell Signaling Technology, Cat# 15102, 1/800 for WB), anti-cGAS (Cell Signaling Technology, Cat# 79978, 1/300 for PLA), anti-β-tubulin (Bioworld, Cat# AP0064, 1/5000), anti-HA tag (Cell Signaling Technology, Cat# 3724, 1/1000), anti-GFP (ABclonal, Cat# AE012, 1/2000), anti-

	phospho-cGAS-S305 (ABclonal, Cat# AP1176, 1/1000), anti-phospho-cGAS-S120 (ABclonal, customized, 1/1000), anti-CHK2 (Cell Signaling Technology, Cat# 2662, 1/1000 for WB), anti-CHK2 (Cell Signaling Technology, Cat# 3440, 1/300 for PLA), anti-phospho-CHK2 (Cell Signaling Technology, Cat# 2661, 1/1000), anti-γH2AX (S139) (Cell Signaling Technology, Cat# 9718, 1/2000 for WB, 1/300 for IF), anti-H2AX (ABclonal, Cat# A11540, 1/2000), anti-Lamin A/C (ABclonal, Cat# A0249, 1/1000), anti-Ki67 (Thermo Fisher, Cat# MA5-14520, 1/500), anti-GAPDH (Proteintech, Cat# 60004, 1/5000), anti-rabbit IgG-HRP (Bio–Rad, Cat# 170-6515), anti-rabbit IgG H&L DyLight 488 (Abcam, Cat# ab96899, 1/500) and anti-mouse IgG H&L DyLight 594 (Abcam, Cat# ab96881, 1/500).
Validation	Flag antibody (MBL, Cat# M185-3L, 1/2000 for WB) was validated by Western blot of cell extracts in HeLa and HEK293T cells. Validation information can be found on the manufacturer's website.<br>Flag antibody (Sigma–Aldrich, Cat. #F3165, 1/300 for PLA) was validated by immunofluorescence analysis of HeLa cells transfected with Flag-tagged ORF2p. Validation information can be found on the manufacturer's website.<br>cGAS antibody (Cell Signaling Technology, Cat# 15102, 1/800 for WB) was validated by WB of cell extracts in HeLa and HEK293T cells. Validation information can be found on the manufacturer's website.<br>cGAS antibody (Cell Signaling Technology, Cat# 79978, 1/300 for PLA) was validated by immunofluorescence analysis of HeLa cells (PMID: 9900338).<br>β-tubulin (Bioworld, Cat# AP0064, 1/5000) antibody was validated by WB of cell extracts in HeLa, HEK293T, TMRT90-hTERT and HCA2-hTERT cells. Validation information can be found on the manufacturer's website.<br>HA antibody (Cell Signaling Technology, Cat# 3724, 1/1000 for WB, 1/300 for PLA) was validated by WB analysis of extracts from HeLa and HET293T cells with expressing HA-tagged TRIM41 or HA-tagged cGAS and its mutants. Validation information can be found on the manufacturer's website.<br>GFP antibody (ABclonal, Cat# AE012, 1/2000) was validated by WB of cell extracts in HEK293T (PMID: 33473130).<br>phospho-cGAS-S305 antibody (ABclonal, Cat# AP1176, 1/1000) was validated by WB of cell extracts in HeLa (PMID: 7186227).<br>phospho-cGAS-S120 antibody (ABclonal, customized, 1/1000) was validated by WB of cell extracts in HeLa. Validation information in Supplementary figure 3h of this paper.<br>CHK2 antibody (Cell Signaling Technology, Cat# 2662, 1/1000 for WB) was validated by WB of cell extracts in HEK293T and HeLa cells. Validation information can be found on the manufacturer's website.<br>CHK2 antibody (Cell Signaling Technology, Cat# 3440, 1/300 for PLA) was validated by immunofluorescence analysis of HeLa cells. Validation information can be found on the manufacturer's website.<br>phospho-CHK2 antibody (Cell Signaling Technology, Cat# 2661, 1/1000) was validated by WB of cell extracts in HeLa cells. Validation information can be found on the manufacturer's website.<br>γH2AX (S139) (Cell Signaling Technology, Cat# 9718, 1/2000 for WB, 1/300 for IF) was validated by immunofluorescence analysis of HeLa cells and WB analysis of HEK193T cells. Validation information can be found on the manufacturer's website.<br>H2AX (ABclonal, Cat# A11540, 1/2000) was validated by WB of cell extracts in HEK293T cells. Validation information can be found on the manufacturer's website.<br>Lamin A/C antibody (ABclonal, Cat# A0249, 1/1000) was validated by WB of cell extracts in HeLa, HEK293T, TMRT90-hTERT and HCA2-hTERT cells. Validation information can be found on the manufacturer's website.<br>Ki67 antibody (Thermo Fisher, Cat# MA5-14520, 1/500) was validated by immunofluorescence analysis of HeLa cells. Validation information can be found on the manufacturer's website.<br>GAPDH antibody (Proteintech, Cat# 60004, 1/5000) was validated by WB of cell extracts in HeLa and HEK293T cells. Validation information can be found on the manufacturer's website.<br>Rabbit IgG H&L DyLight 488 antibody (Abcam, Cat# ab96899, 1/500) was validated by immunofluorescence WB of cell extracts in HeLa and HEK293T cells. Validation information can be found on the manufacturer's website.<br>Mouse IgG H&L DyLight 594 (Abcam, Cat# ab96881, 1/500) was validated by immunofluorescence WB of cell extracts in HeLa and HEK293T cells. Validation information can be found on the manufacturer's website.

Eukaryotic cell lines

Policy information about cell lines and Sex and Gender in Research

Cell line source(s)	Human embryonic kidney epithelial cells (HEK293T; ATCC CRL-11268), human cervical adenocarcinoma epithelial cells (HeLa; ATCC CRM-CCL-2), IMRT90 (an immortalized human lung fibroblast cell line), HCA1-hTERT (an immortalized foreskin fibroblast cell line) and human fibroblast H15C cells were originated from Vera Gorbunova's lab (University of Rochester, Rochester, NY).
Authentication	The cells were authenticated by morphology.
Mycoplasma contamination	The cells were routinely checked for mycoplasmas to secure mycoplasma-free.
Commonly misidentified lines<br>(See ICLAC register)	None of the cell lines used are listed in the ICLAC database.

Animals and other research organisms

Policy information about studies involving animals; ARRIVE guidelines recommended for reporting animal research, and Sex and Gender in Research

Laboratory animals	Mice were housed in a specific pathogen-free (SPF)-grade environment with temperature ranging from 23-27°C, humidity of 30-45%. All mice were maintained under a 12-hour light/dark cycle and had free access to food and water. All experiments were performed in accordance with the Health Guide for the Care and Use of Laboratory Animals and were approved by the Biological Research Ethics Committee. Both 3-4-month old male and female Cgas knockout mice and wild type mice were included in this study, however, no sex-based analysis was performed because the number of mice utilized is insufficient to draw clear sex-based conclusions.

Wild animals	The study did not involve wild animals.
Reporting on sex	Both 3-4-month old male and female Cgas knockout mice and wild type mice were included in this study, however, no sex-based analysis was performed because the number of mice utilized is insufficient to draw clear sex-based conclusions.
Field-collected samples	No field-collected samples were used in this study.
Ethics oversight	All experiments were performed in accordance with the Health Guide for the Care and Use of Laboratory Animals and were approved by the Biological Research Ethics Committee of Tongji University (TJAB04022103).

Note that full information on the approval of the study protocol must also be provided in the manuscript.

Plants

Seed stocks	The study did not involve plants.
Novel plant genotypes	The study did not involve plants.
Authentication	The study did not involve plants.

Flow Cytometry

Plots

Confirm that:

- [x] The axis labels state the marker and fluorochrome used (e.g. CD4-FITC).
- [x] The axis scales are clearly visible. Include numbers along axes only for bottom left plot of group (a 'group' is an analysis of identical markers).
- [x] All plots are contour plots with outliers or pseudocolor plots.
- [x] A numerical value for number of cells or percentage (with statistics) is provided.

Methodology

Sample preparation	See methods section entitled: L1 retrotransposition assay.
Instrument	BD FACSVerse (BD Biosciences, San Jose, CA).
Software	FlowJo software (Ashland, OR).
Cell population abundance	At least 10,000 cells were included for each replicate.
Gating strategy	Following routine gating strategy.

- [ ] Tick this box to confirm that a figure exemplifying the gating strategy is provided in the Supplementary Information.
