# Supplementary material for: Nuclear cGAS restricts L1 retrotransposition by promoting TRIM41-mediated ORF2p ubiquitination and degradation
Source: Nat Commun. 2023 Dec 12;14:8217. doi: 10.1038/s41467-023-43001-y (PMC10716122; doi:10.1038/s41467-023-43001-y)

Fig. 1b

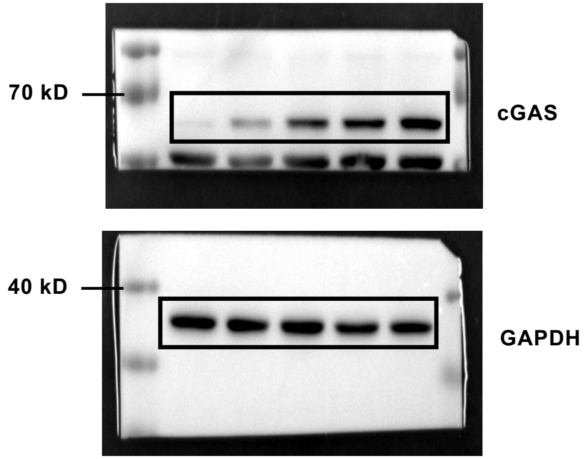

Fig. 1f

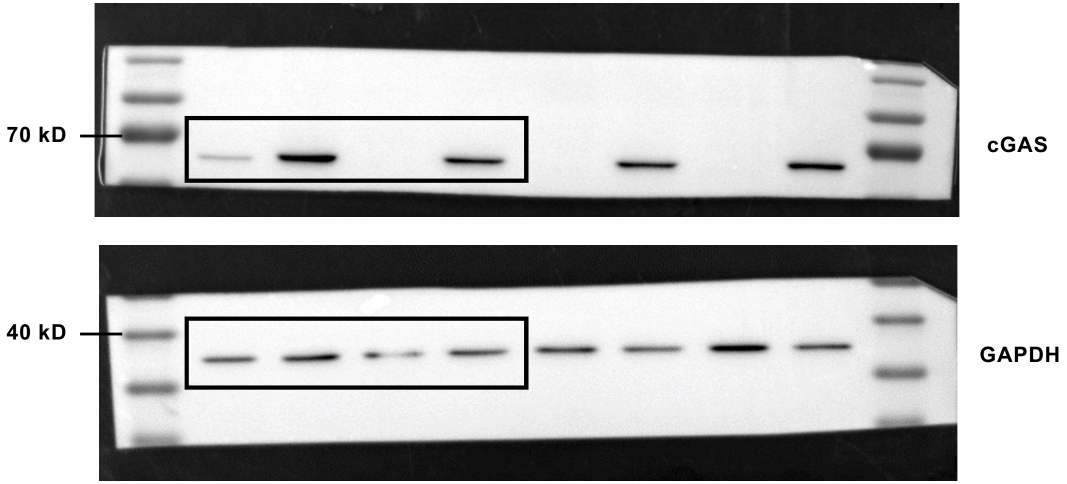

Fig. 1d

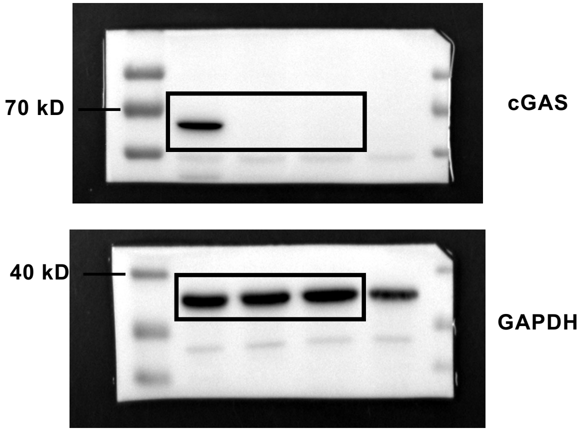

Fig. 1j

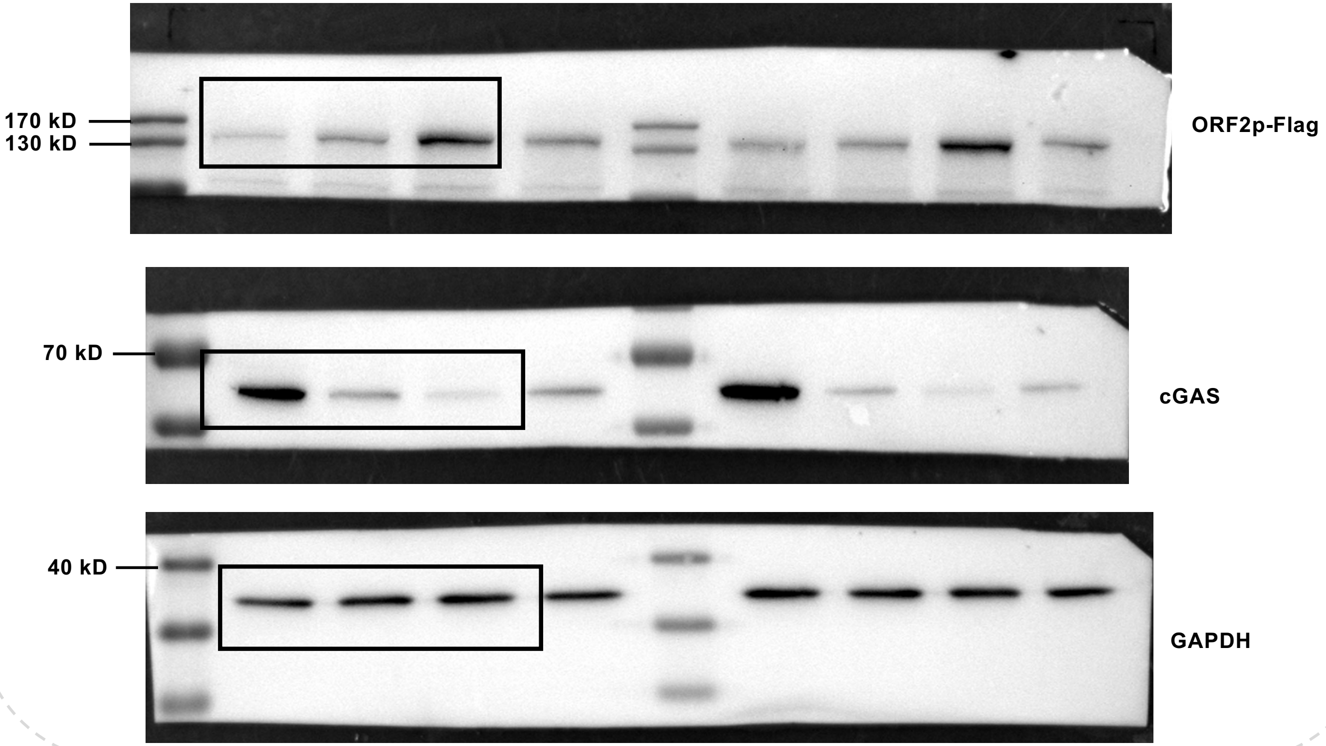

Fig. 1k

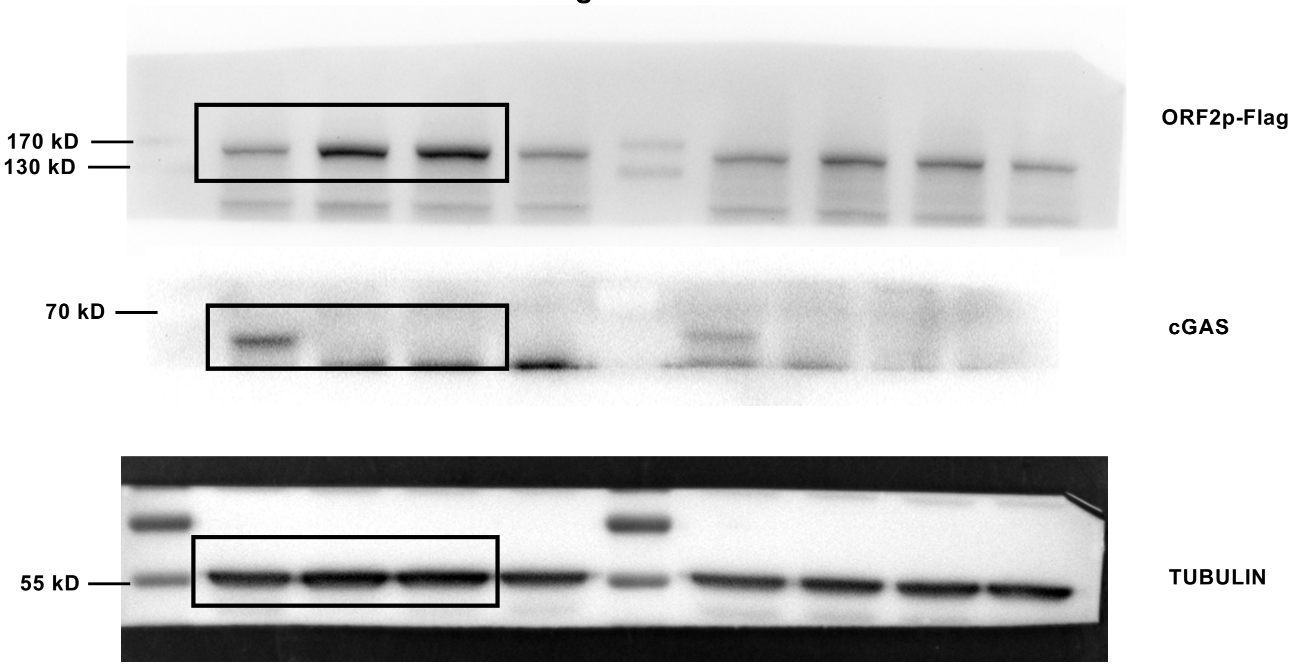
170 kD
130 kD
ORF2p-Flag
70 kD
cGAS
55 kD
TUBULIN

Fig. 1l

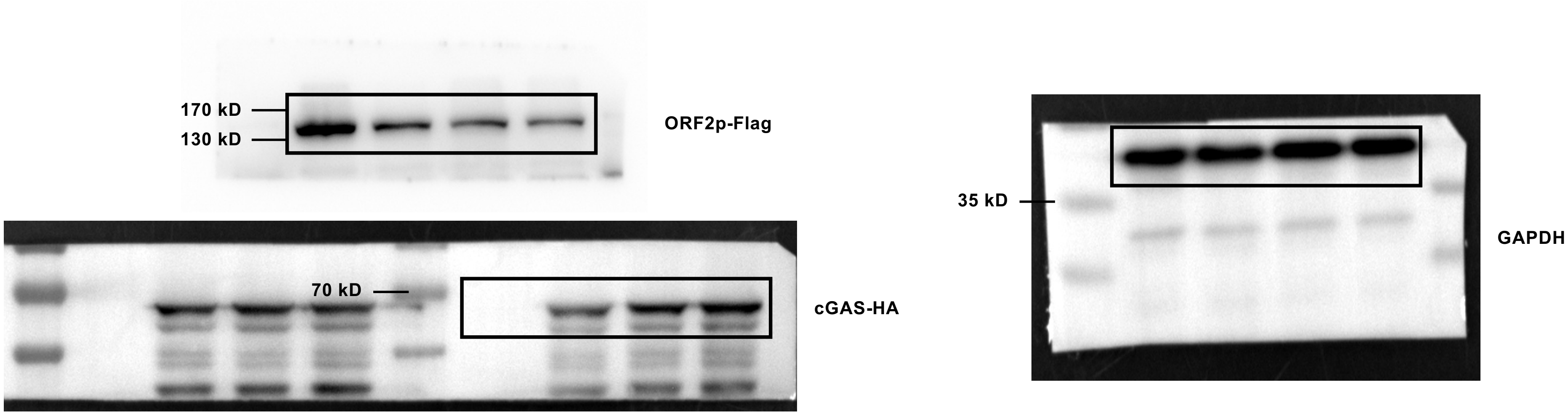
170 kD
130 kD
ORF2p-Flag
70 kD
cGAS-HA
35 kD
GAPDH

Fig. 2a

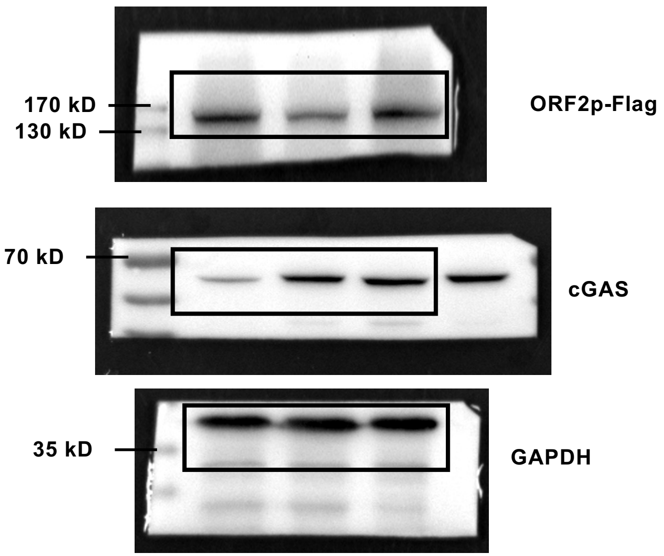
170 kD
130 kD
ORF2p-Flag
70 kD
cGAS
35 kD
GAPDH

Fig. 2c

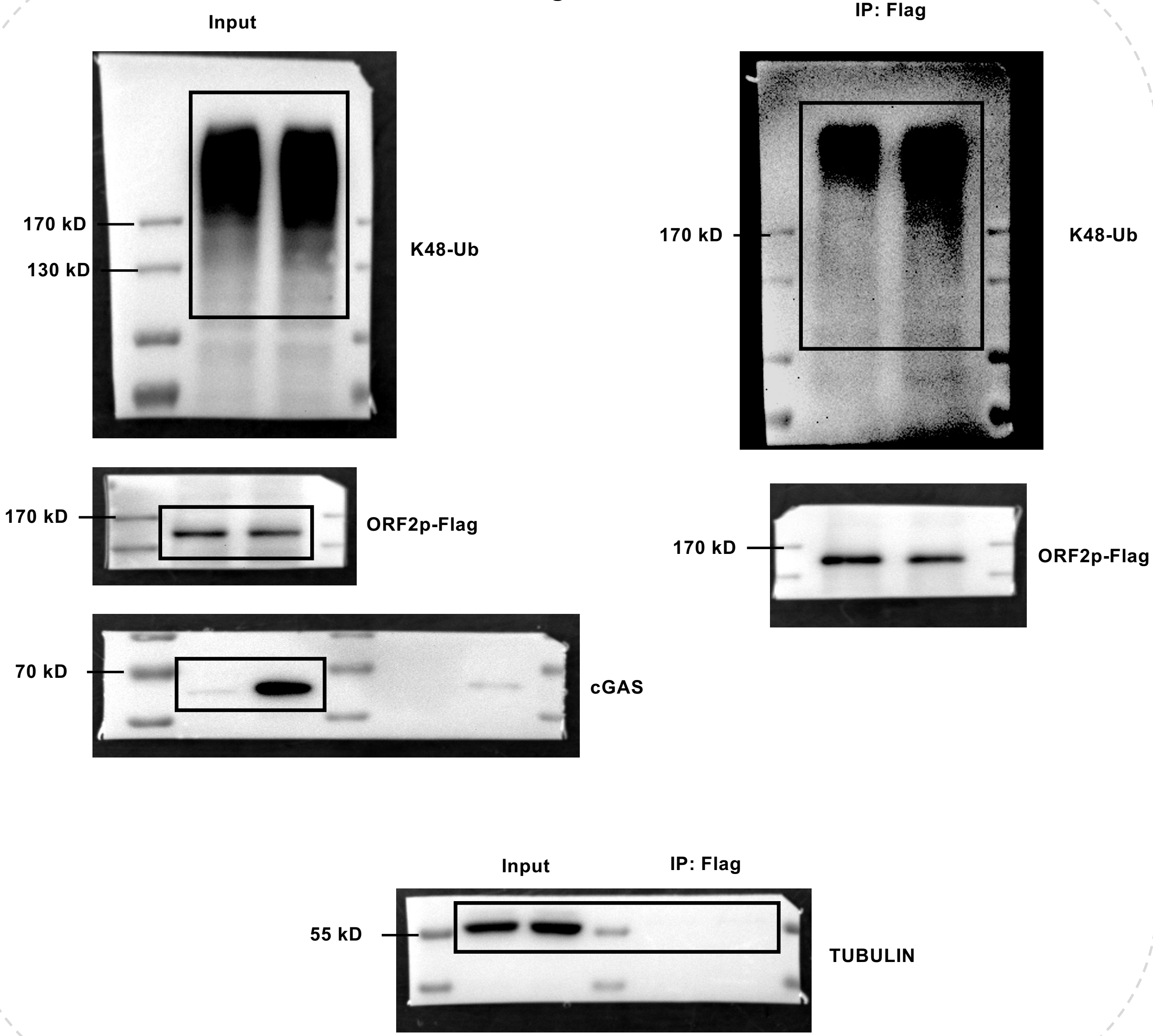
Input
IP: Flag
170 kD
130 kD
K48-Ub
170 kD
K48-Ub
170 kD
ORF2p-Flag
170 kD
ORF2p-Flag
70 kD
cGAS
Input
IP: Flag
55 kD
TUBULIN

Fig. 2d

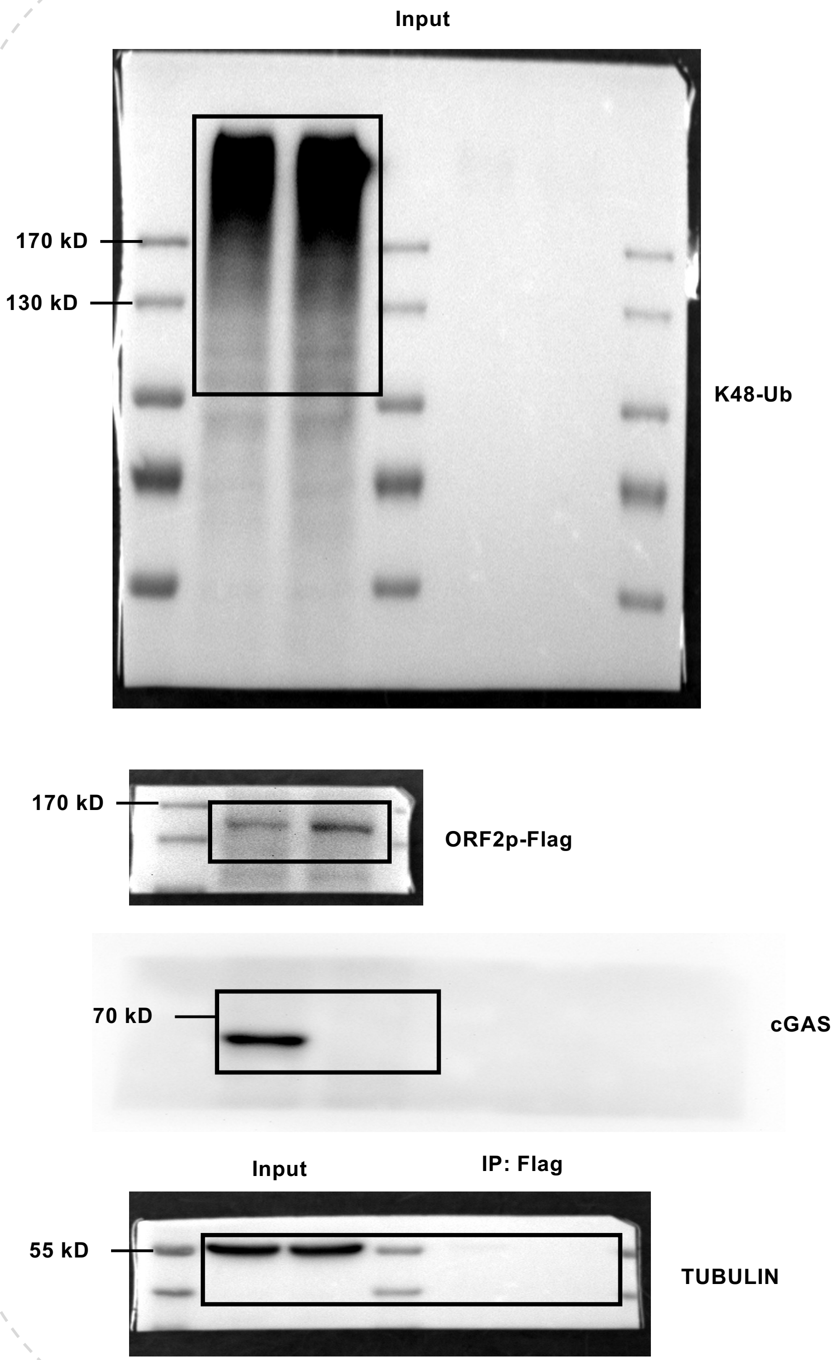

Input
IP: Flag
170 kD
130 kD
K48-Ub
170 kD
130 kD
K48-Ub
170 kD
ORF2p-Flag
170 kD
ORF2p-Flag
70 kD
cGAS
Input
IP: Flag
55 kD
TUBULIN

Fig. 2f

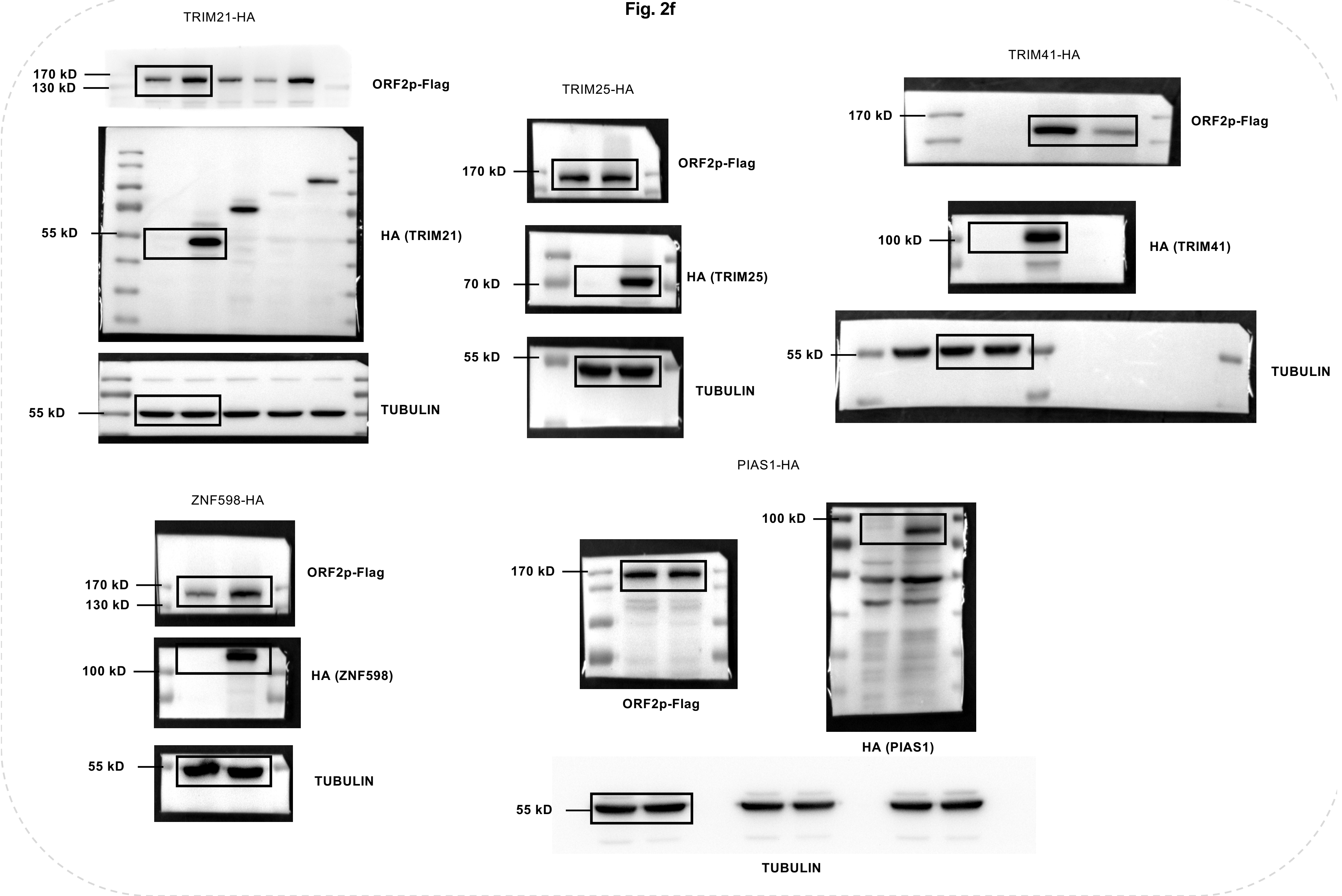
TRIM21-HA
170 kD
130 kD
ORF2p-Flag
55 kD
HA (TRIM21)
55 kD
TUBULIN
TRIM25-HA
170 kD
ORF2p-Flag
70 kD
HA (TRIM25)
55 kD
TUBULIN
TRIM41-HA
170 kD
ORF2p-Flag
100 kD
HA (TRIM41)
55 kD
TUBULIN
ZNF598-HA
170 kD
130 kD
ORF2p-Flag
100 kD
HA (ZNF598)
55 kD
TUBULIN
PIAS1-HA
170 kD
ORF2p-Flag
100 kD
HA (PIAS1)
55 kD
TUBULIN

Fig. 2g

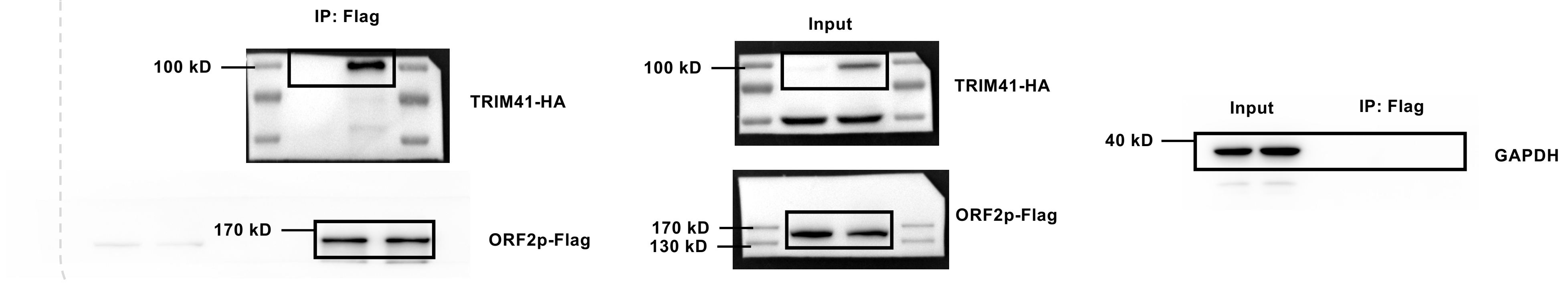
IP: Flag
100 kD
TRIM41-HA
170 kD
ORF2p-Flag
Input
100 kD
TRIM41-HA
170 kD
130 kD
ORF2p-Flag
Input
IP: Flag
40 kD
GAPDH

Fig. 2h

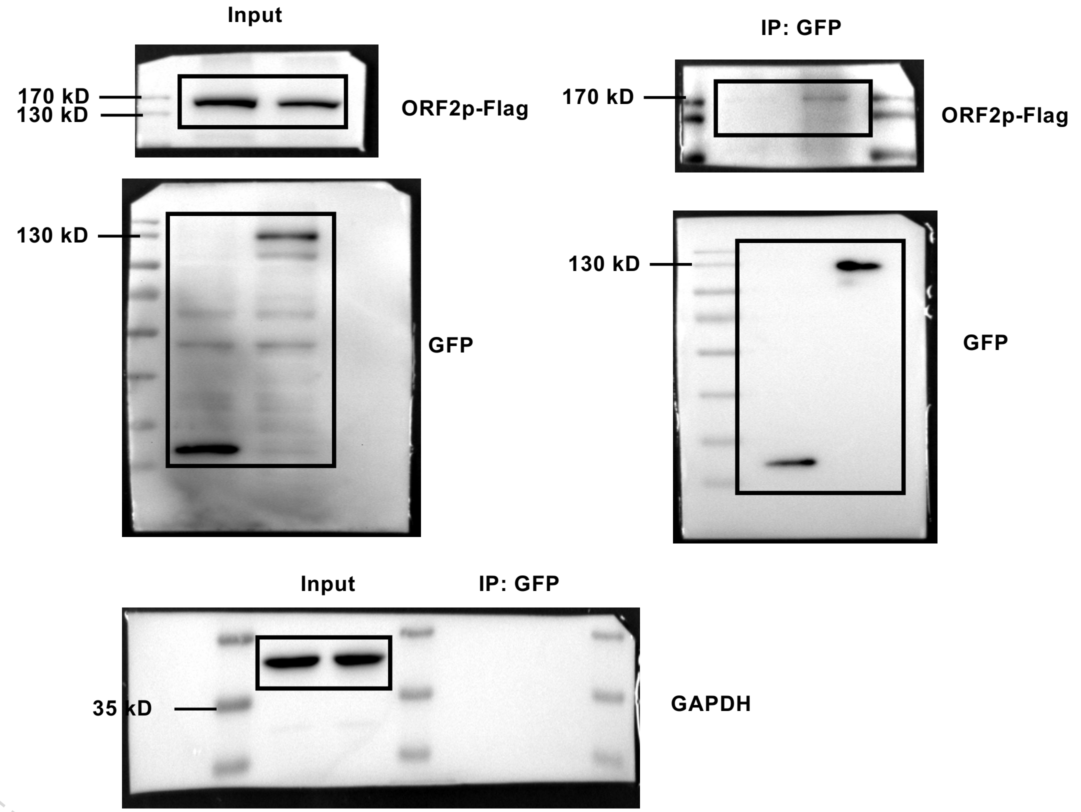
Input
170 kD
130 kD
ORF2p-Flag
130 kD
GFP
IP: GFP
170 kD
ORF2p-Flag
130 kD
GFP
Input
IP: GFP
35 kD
GAPDH

Fig. 2i

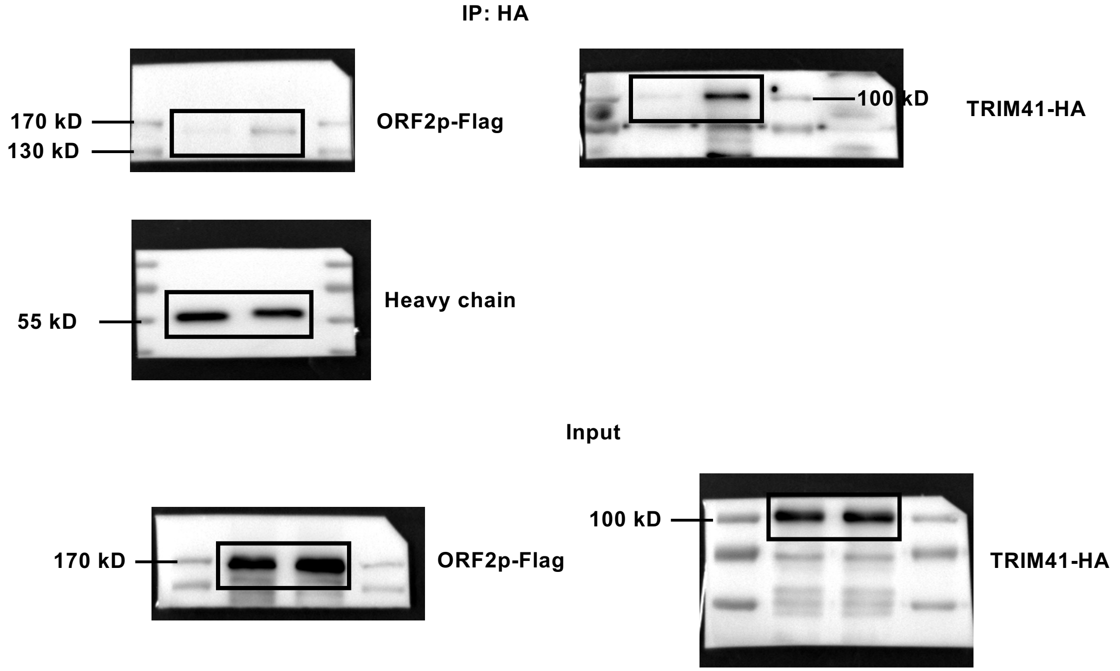
IP: HA
170 kD
130 kD
ORF2p-Flag
100 kD
TRIM41-HA
55 kD
Heavy chain
Input
170 kD
ORF2p-Flag
100 kD
TRIM41-HA

Fig. 2j

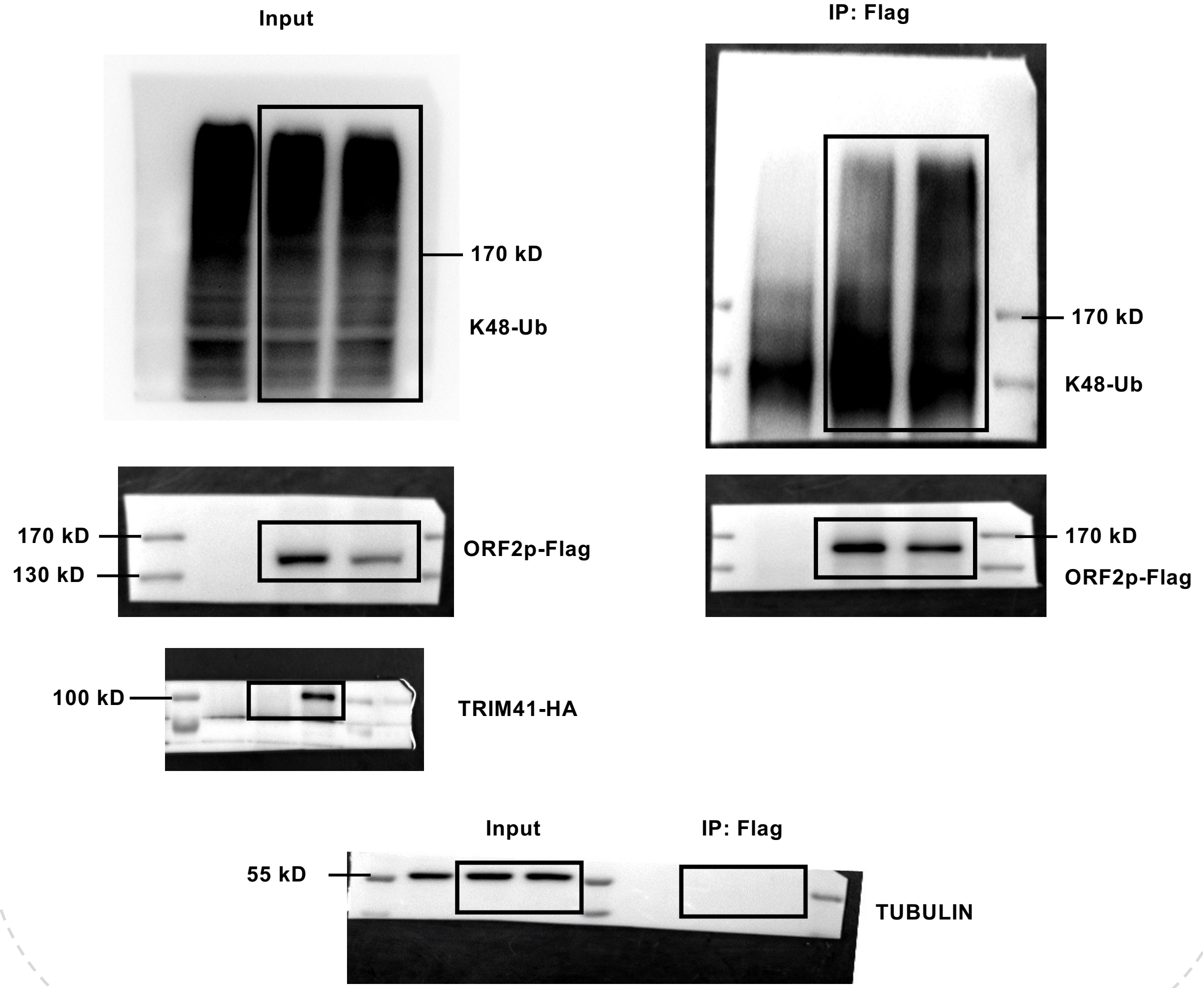
Input
IP: Flag
170 kD
K48-Ub
170 kD
K48-Ub
170 kD
130 kD
ORF2p-Flag
170 kD
ORF2p-Flag
100 kD
TRIM41-HA
Input
IP: Flag
55 kD
TUBULIN

Fig. 2k

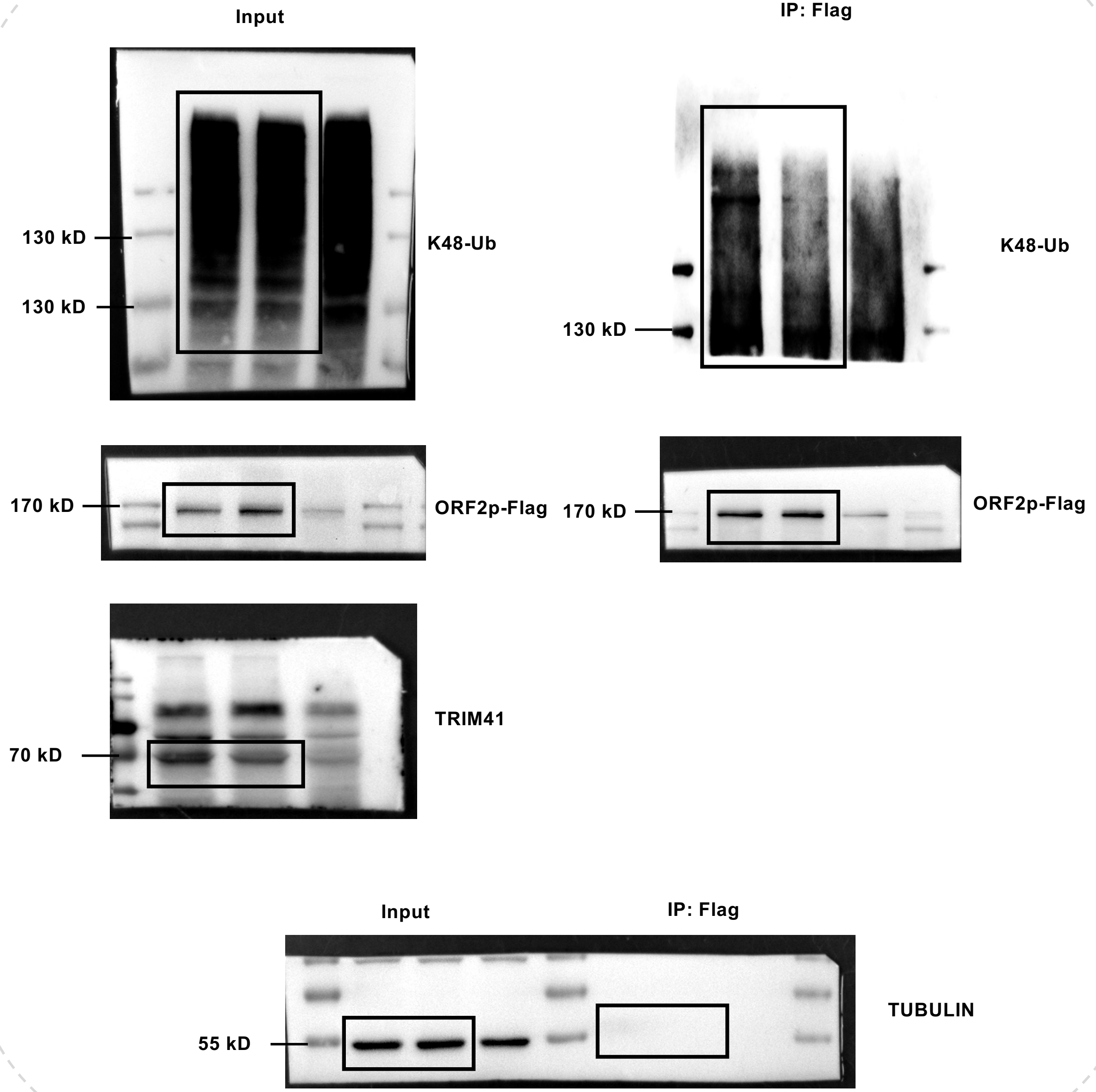

Input
IP: Flag
130 kD
130 kD
K48-Ub
130 kD
K48-Ub
170 kD
ORF2p-Flag
170 kD
ORF2p-Flag
70 kD
TRIM41
Input
IP: Flag
55 kD
TUBULIN

Fig. 2I

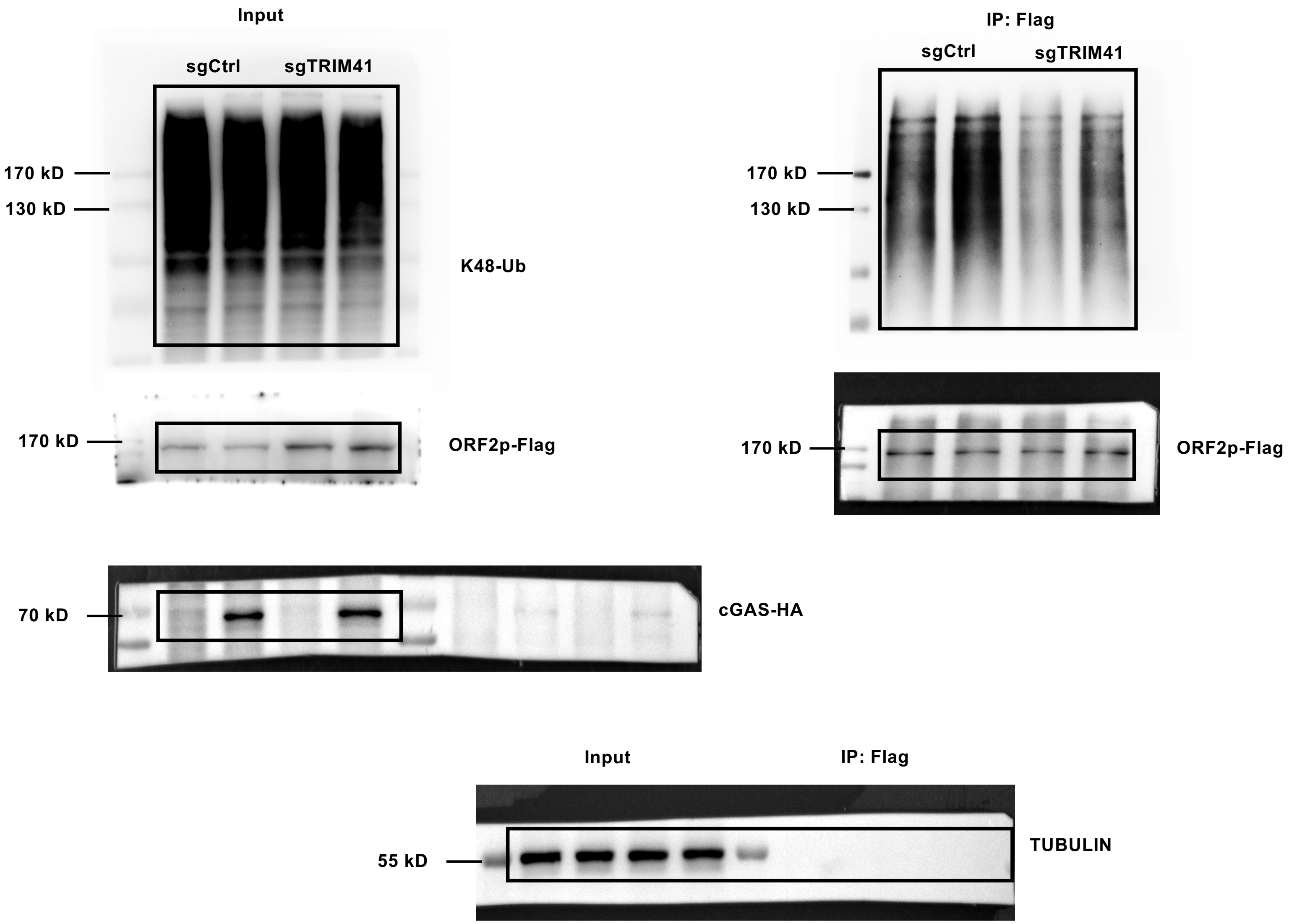
Input
sgCtrl
sgTRIM41
170 kD
130 kD
K48-Ub
IP: Flag
sgCtrl
sgTRIM41
170 kD
130 kD
170 kD
ORF2p-Flag
170 kD
ORF2p-Flag
70 kD
cGAS-HA
Input
IP: Flag
55 kD
TUBULIN

Fig. 2m

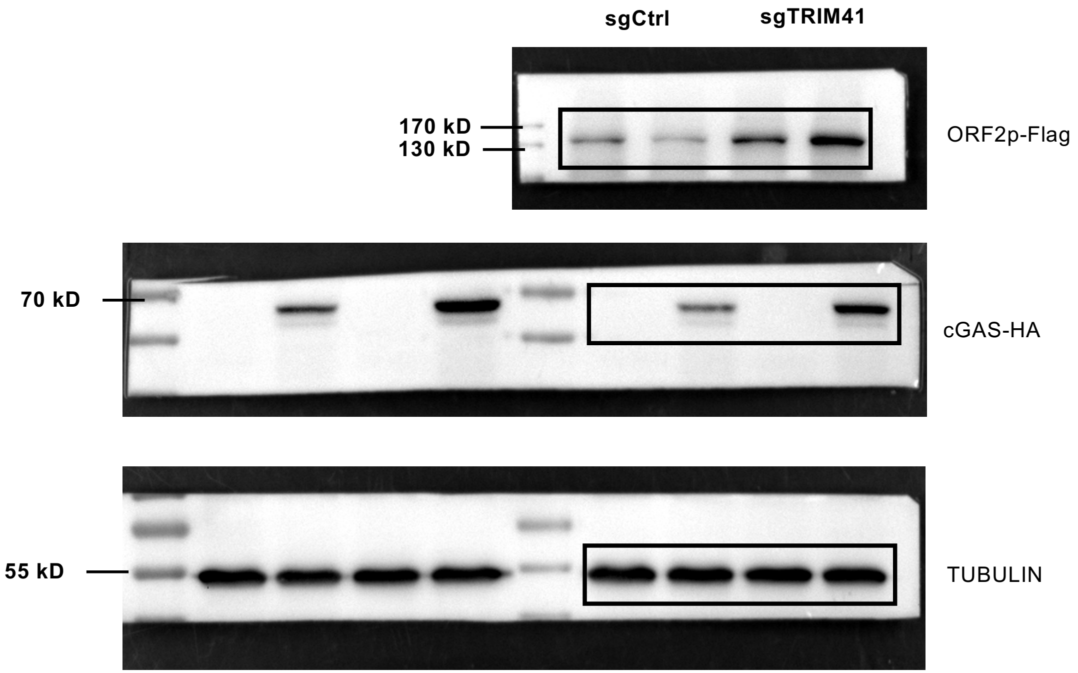
sgCtrl
sgTRIM41
170 kD
130 kD
ORF2p-Flag
70 kD
cGAS-HA
55 kD
TUBULIN

Fig. 3b

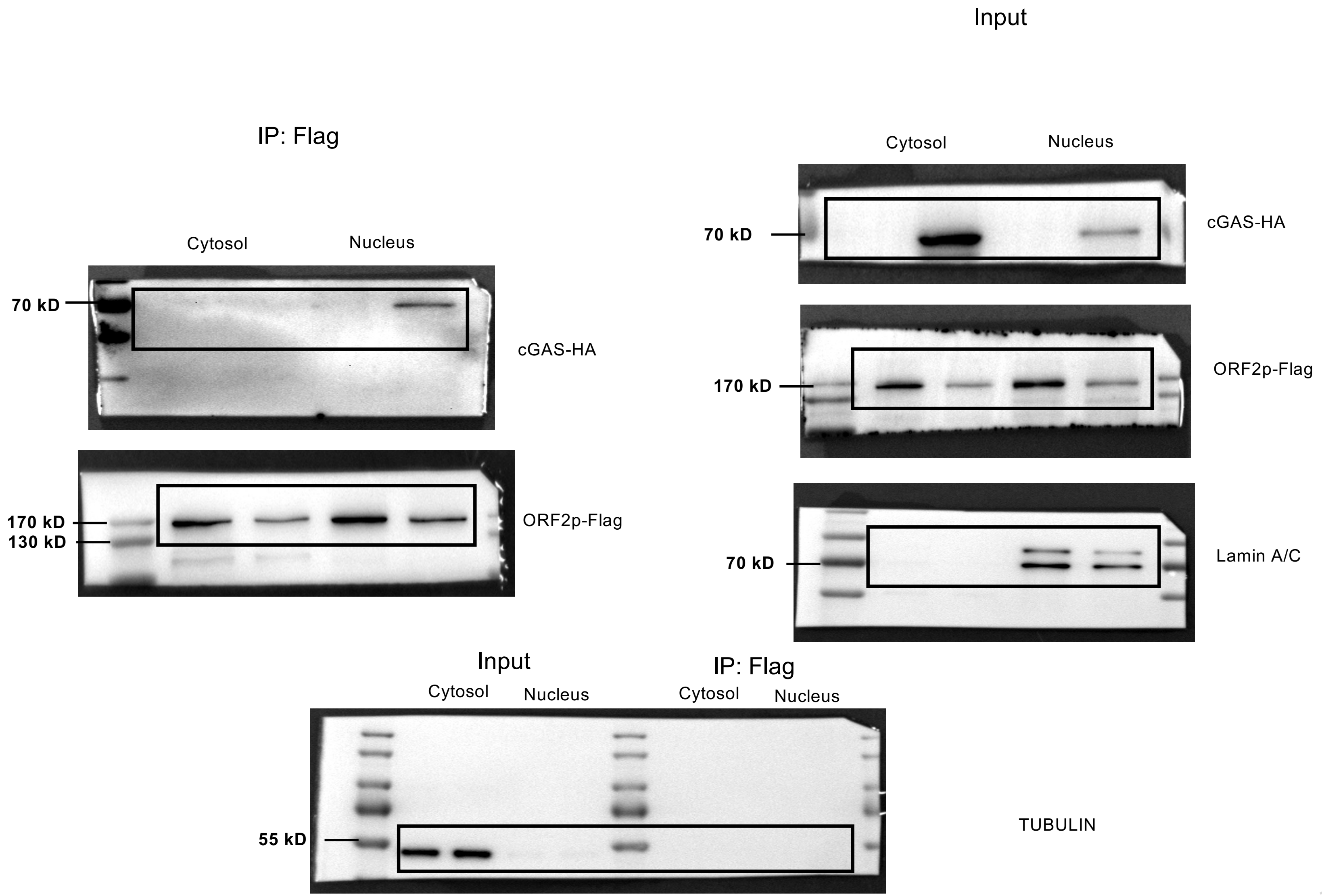

Input
IP: Flag
Cytosol
Nucleus
70 kD
cGAS-HA
170 kD
130 kD
ORF2p-Flag
Cytosol
Nucleus
70 kD
cGAS-HA
170 kD
ORF2p-Flag
70 kD
Lamin A/C
Input
IP: Flag
Cytosol
Nucleus
Cytosol
Nucleus
55 kD
TUBULIN

Fig. 3c

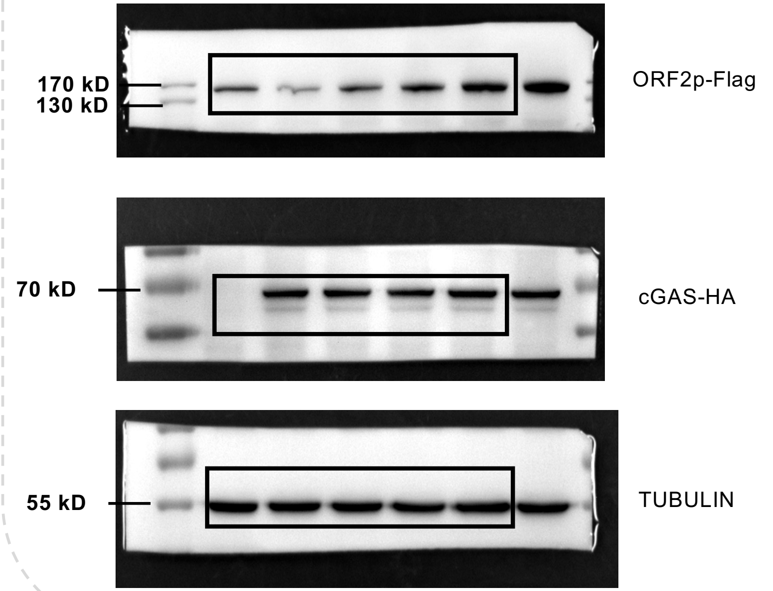
170 kD
130 kD
ORF2p-Flag
70 kD
cGAS-HA
55 kD
TUBULIN

Fig. 3d

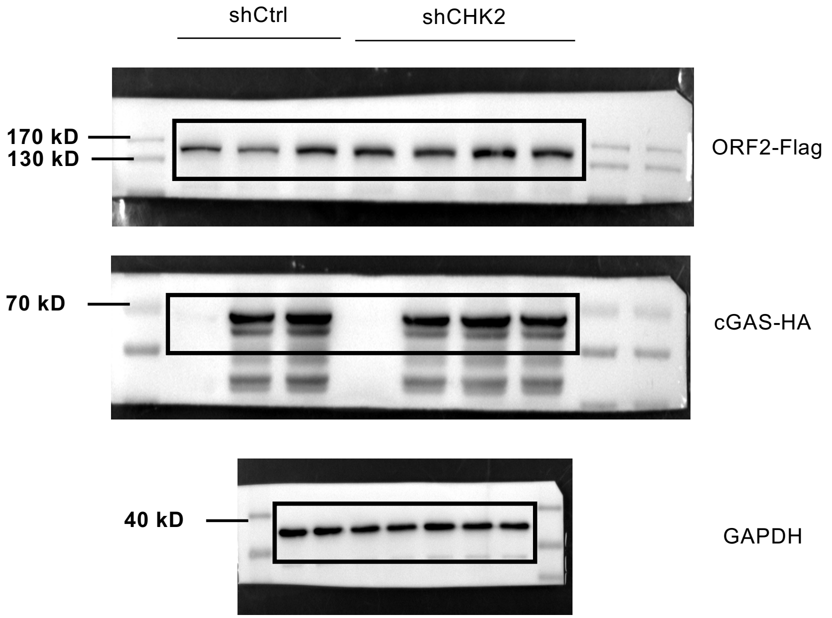
shCtrl
shCHK2
170 kD
130 kD
ORF2-Flag
70 kD
cGAS-HA
40 kD
GAPDH

Fig. 3e

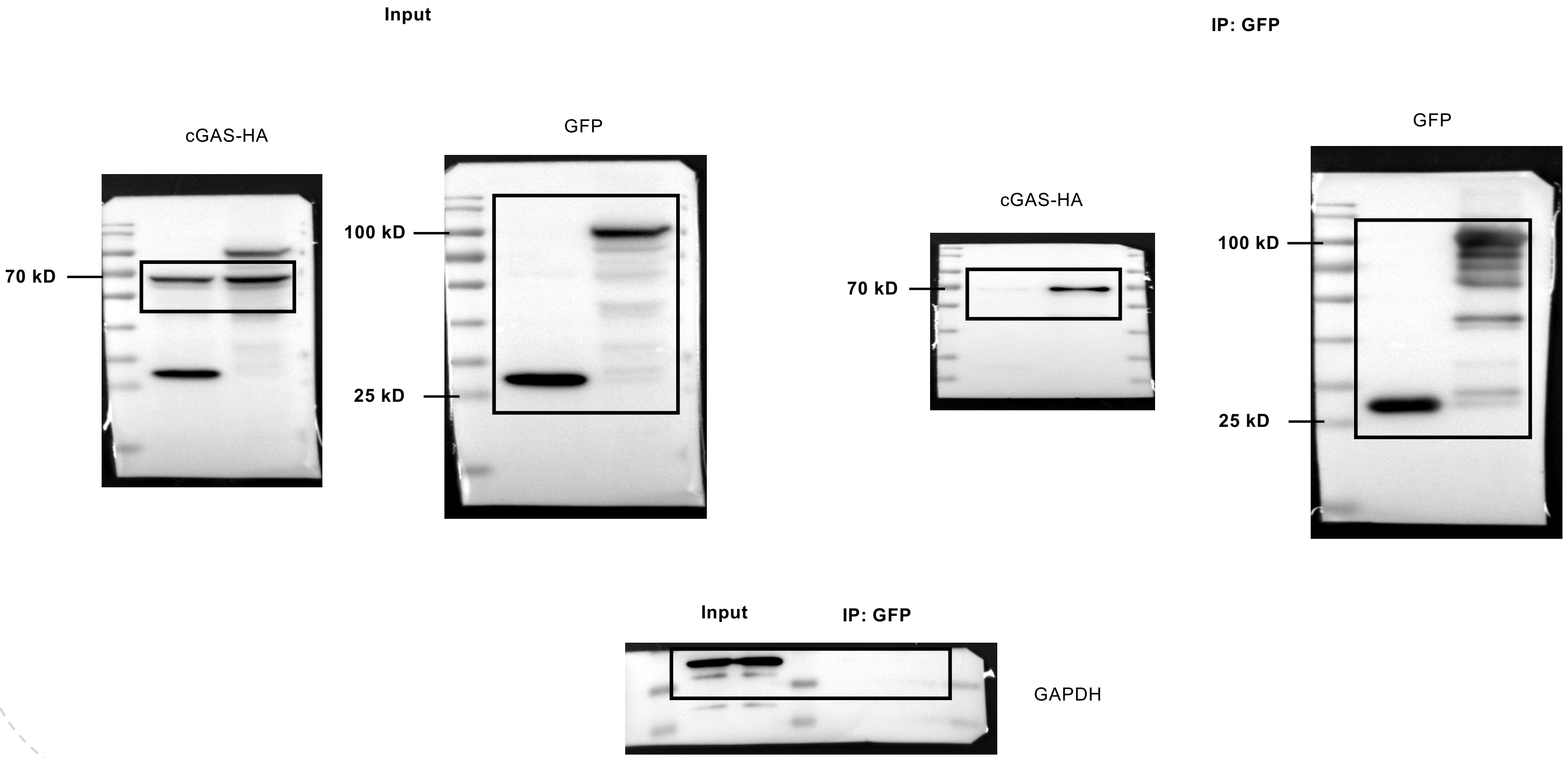
Input
IP: GFP
cGAS-HA
GFP
70 kD
100 kD
25 kD
cGAS-HA
GFP
70 kD
100 kD
25 kD
Input
IP: GFP
GAPDH

Fig. 3f

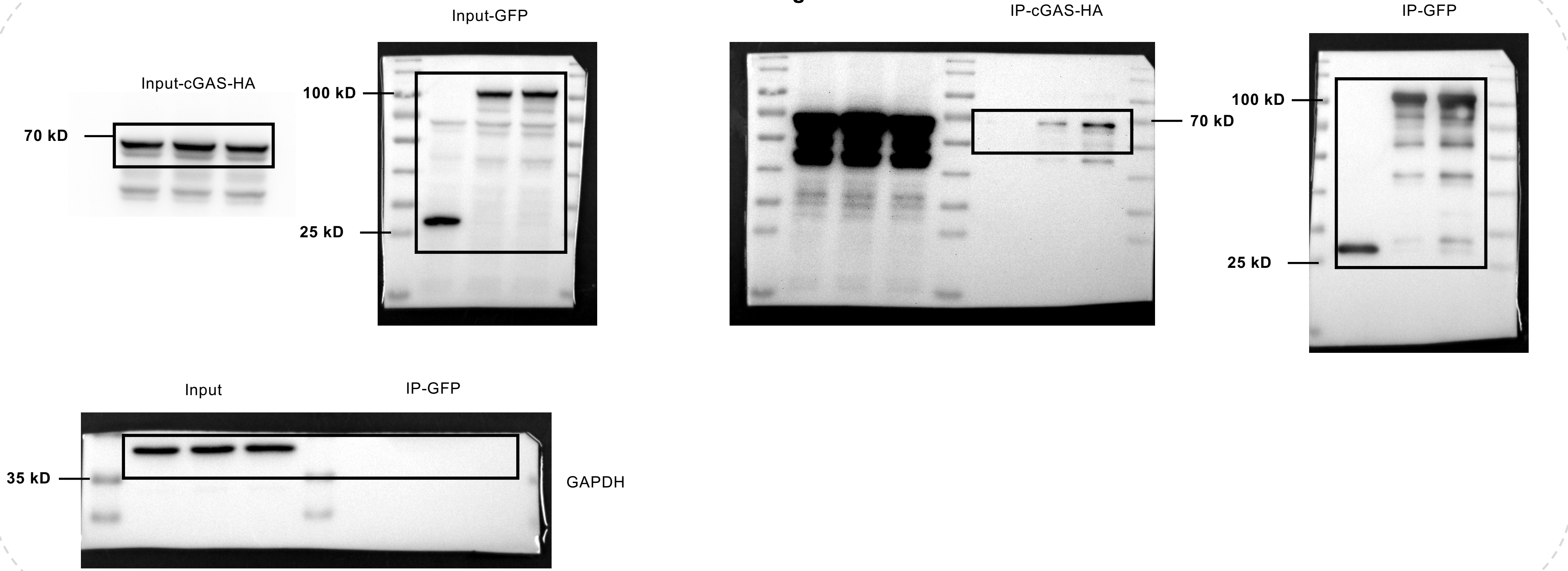

Input-cGAS-HA
70 kD
Input-GFP
100 kD
25 kD
IP-cGAS-HA
70 kD
IP-GFP
100 kD
25 kD
Input
IP-GFP
35 kD
GAPDH

Fig. 3g

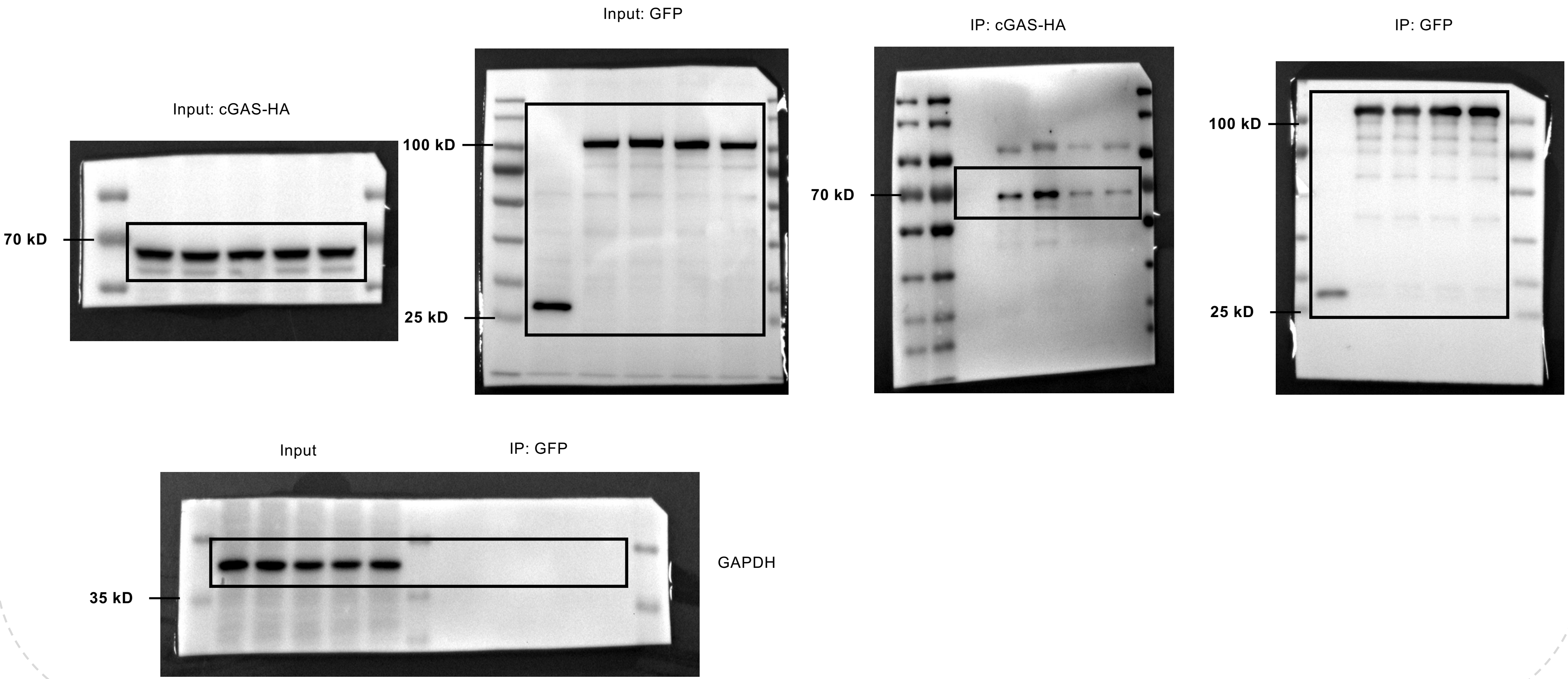
Input: cGAS-HA
70 kD
Input: GFP
100 kD
25 kD
IP: cGAS-HA
70 kD
IP: GFP
100 kD
25 kD
Input
IP: GFP
35 kD
GAPDH

Fig. 3i

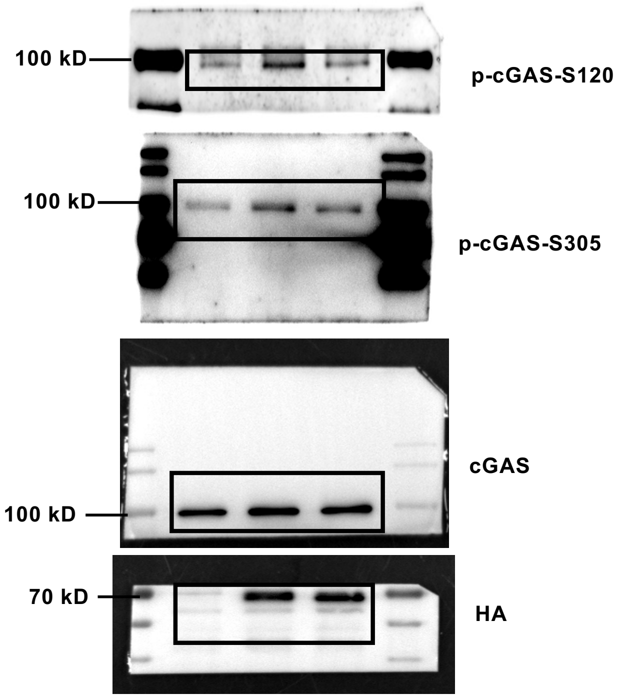
100 kD
p-cGAS-S120
100 kD
p-cGAS-S305
cGAS
100 kD
70 kD
HA

Fig. 3j

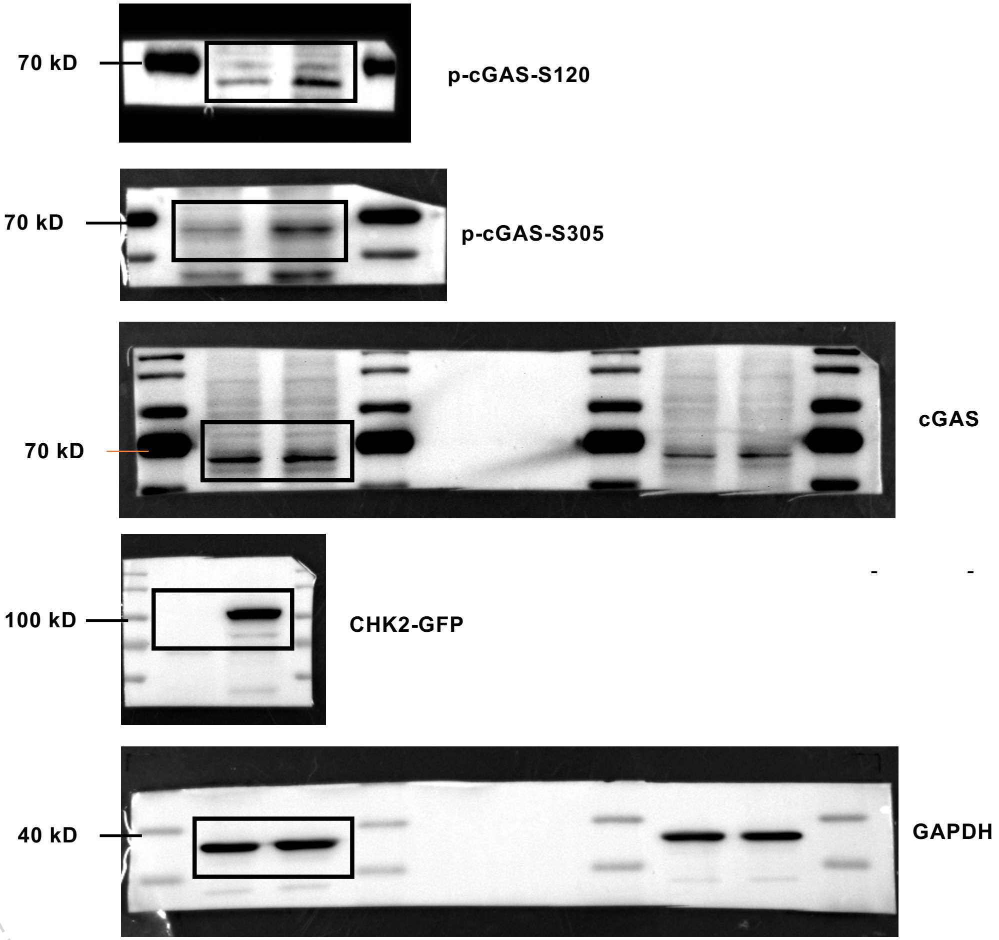
70 kD
p-cGAS-S120
70 kD
p-cGAS-S305
70 kD
cGAS
100 kD
CHK2-GFP
40 kD
GAPDH

Fig. 3k

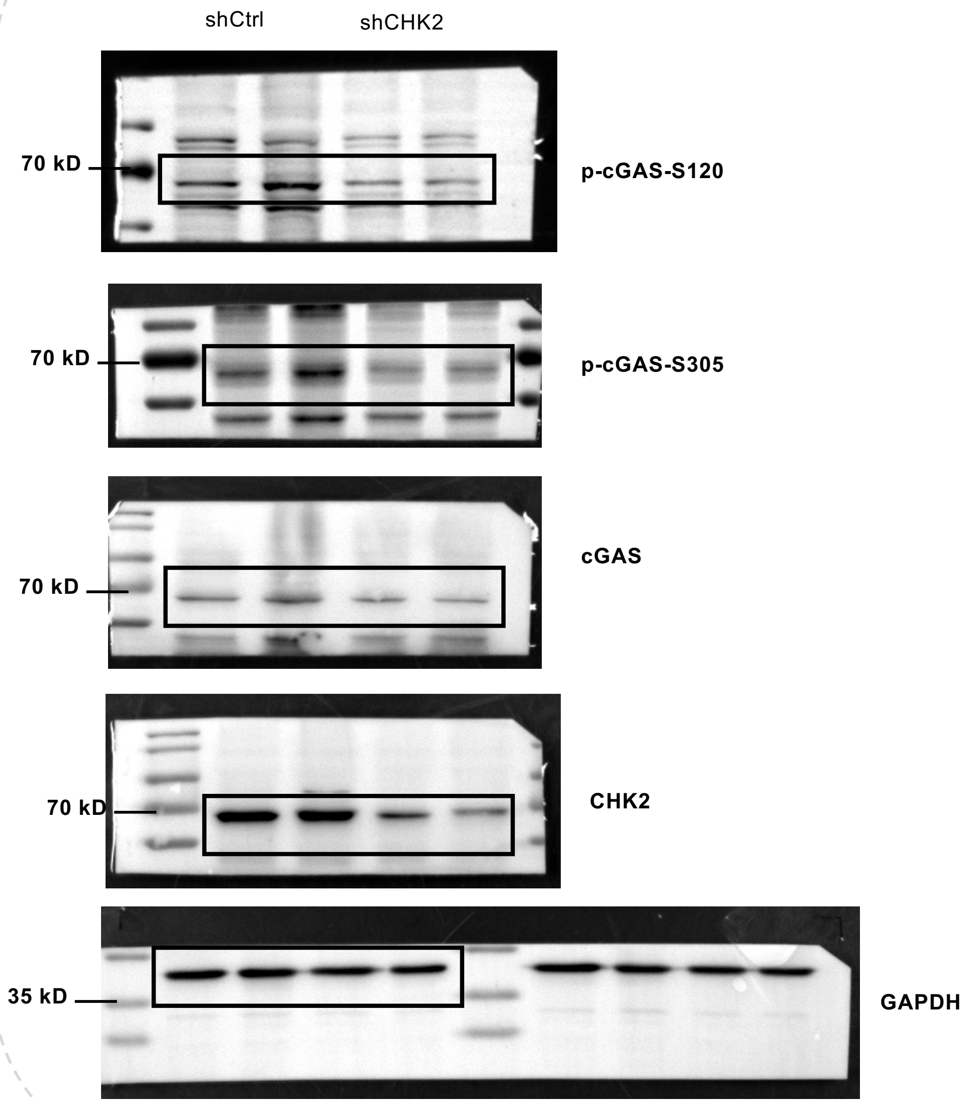
shCtrl
shCHK2
70 kD
p-cGAS-S120
70 kD
p-cGAS-S305
70 kD
cGAS
70 kD
CHK2
35 kD
GAPDH

Fig. 3I

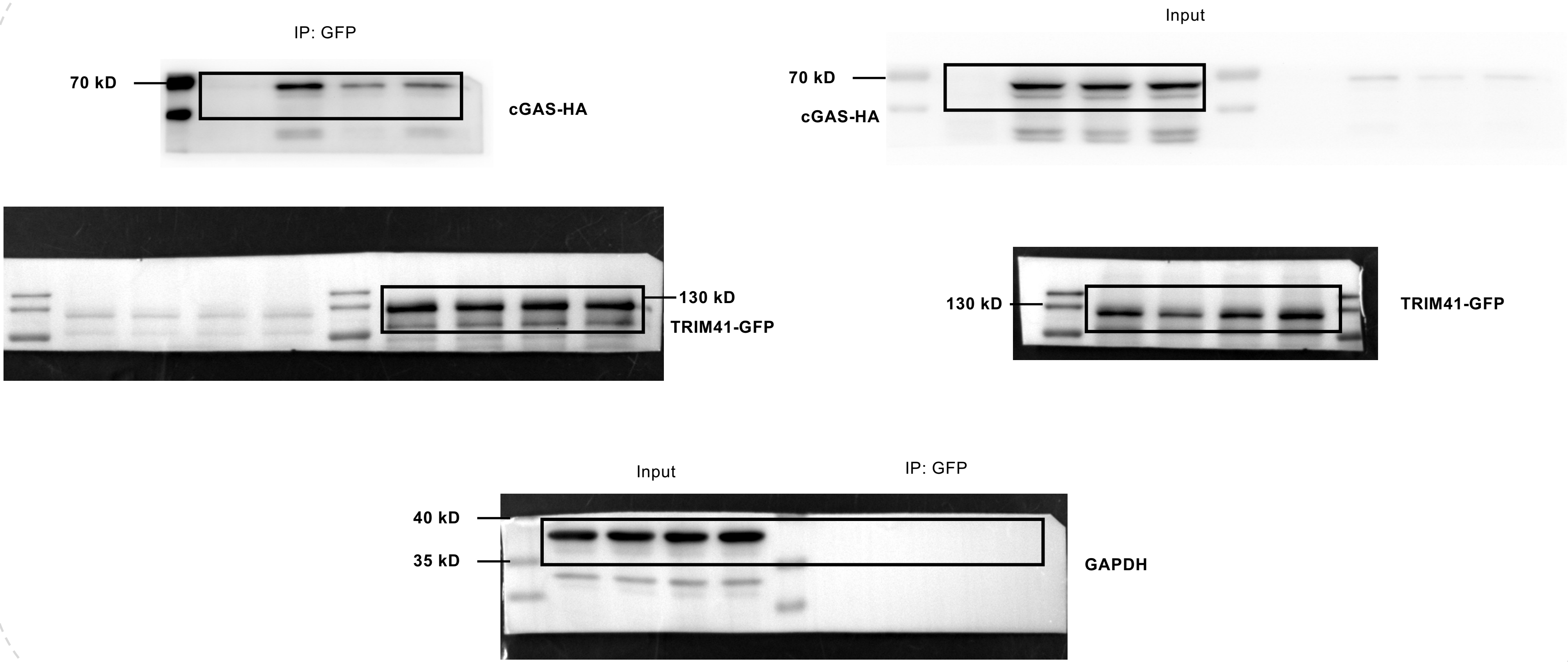
IP: GFP
70 kD
cGAS-HA
Input
70 kD
cGAS-HA
130 kD
TRIM41-GFP
130 kD
TRIM41-GFP
Input
IP: GFP
40 kD
35 kD
GAPDH

Fig. 3m

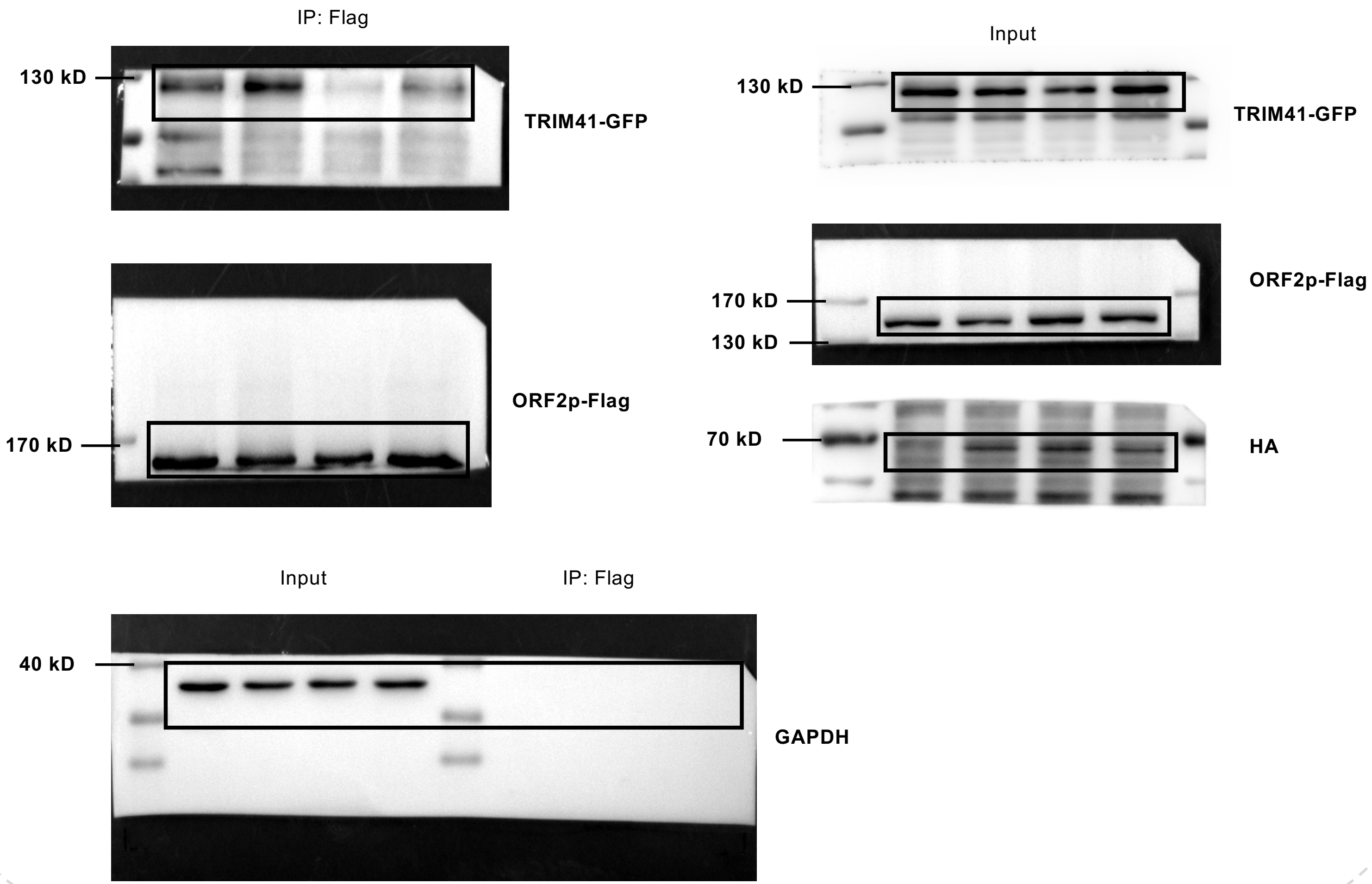
IP: Flag
130 kD
TRIM41-GFP
170 kD
ORF2p-Flag
Input
130 kD
TRIM41-GFP
170 kD
130 kD
ORF2p-Flag
70 kD
HA
Input
IP: Flag
40 kD
GAPDH

Fig. 3n

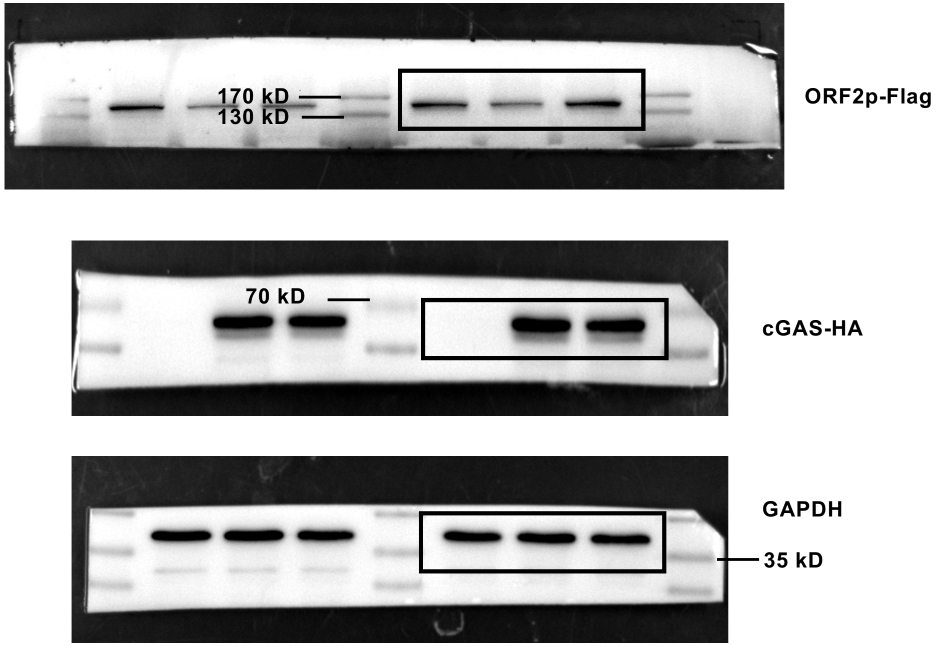

Fig. 4c

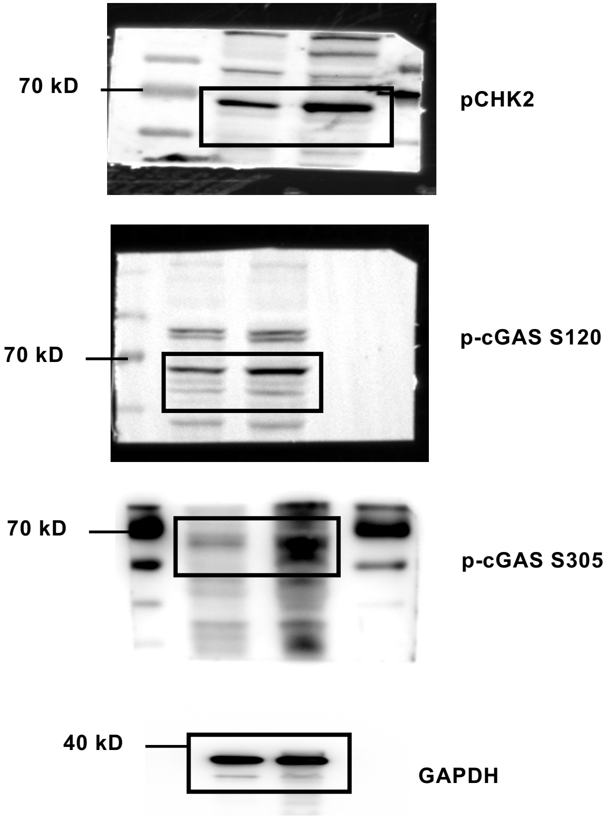
70 kD
pCHK2
p-cGAS S120
70 kD
70 kD
p-cGAS S305
40 kD
GAPDH

Fig. 4d

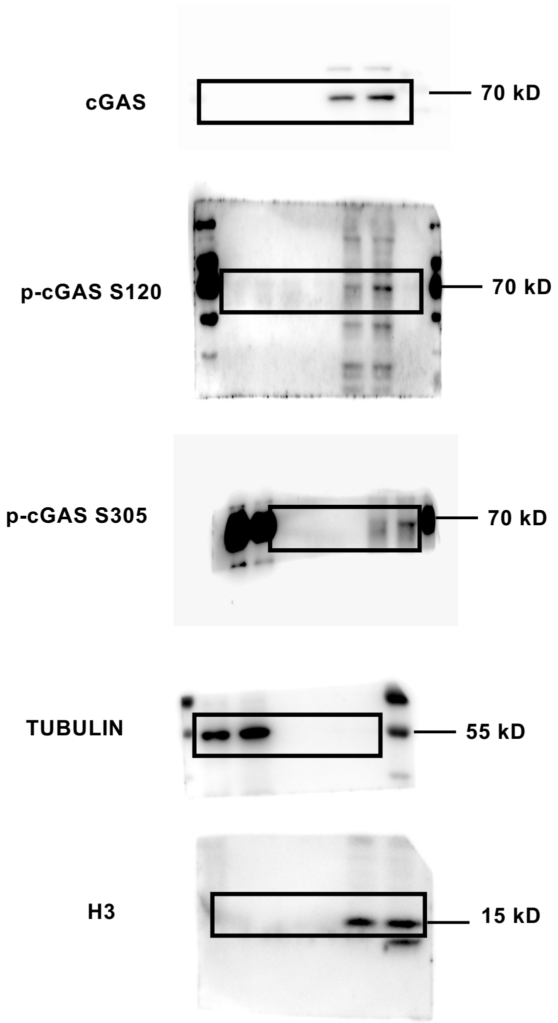
cGAS
70 kD
p-cGAS S120
70 kD
p-cGAS S305
70 kD
TUBULIN
55 kD
H3
15 kD

Fig. 4e

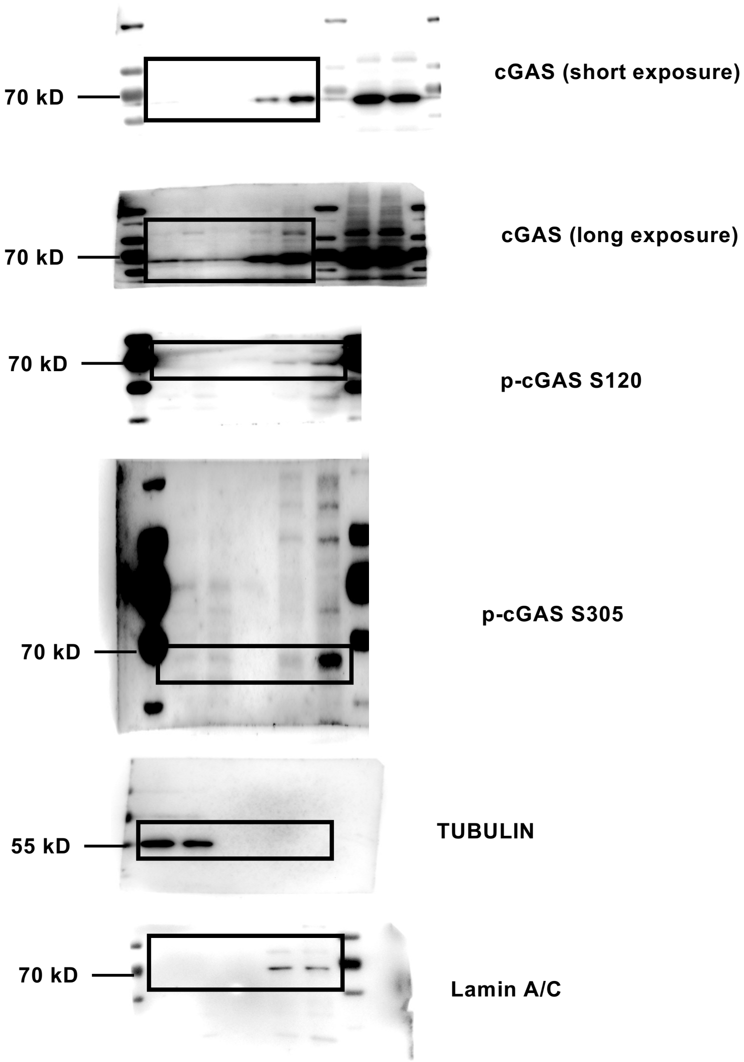
70 kD
cGAS (short exposure)
70 kD
cGAS (long exposure)
70 kD
p-cGAS S120
p-cGAS S305
70 kD
55 kD
TUBULIN
70 kD
Lamin A/C

Fig. 4f

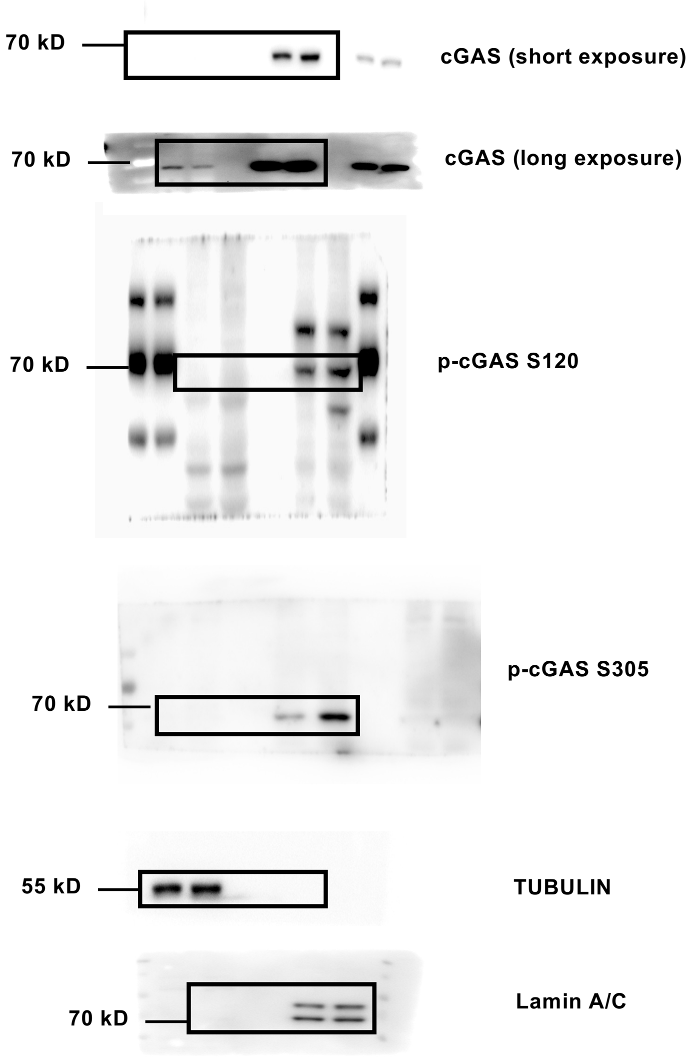
70 kD
cGAS (short exposure)
70 kD
cGAS (long exposure)
70 kD
p-cGAS S120
p-cGAS S305
70 kD
55 kD
TUBULIN
Lamin A/C
70 kD

Fig. 5c

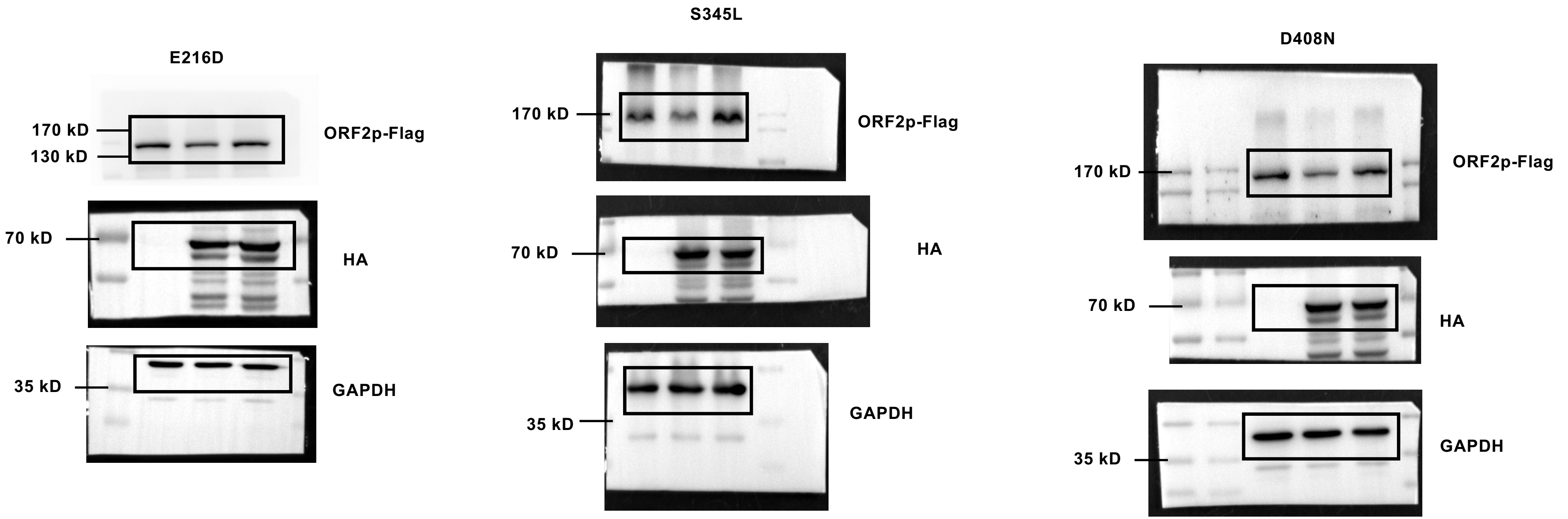
E216D
170 kD
130 kD
ORF2p-Flag
70 kD
HA
35 kD
GAPDH
S345L
170 kD
ORF2p-Flag
70 kD
HA
35 kD
GAPDH
D408N
170 kD
ORF2p-Flag
70 kD
HA
35 kD
GAPDH

Fig. 5c

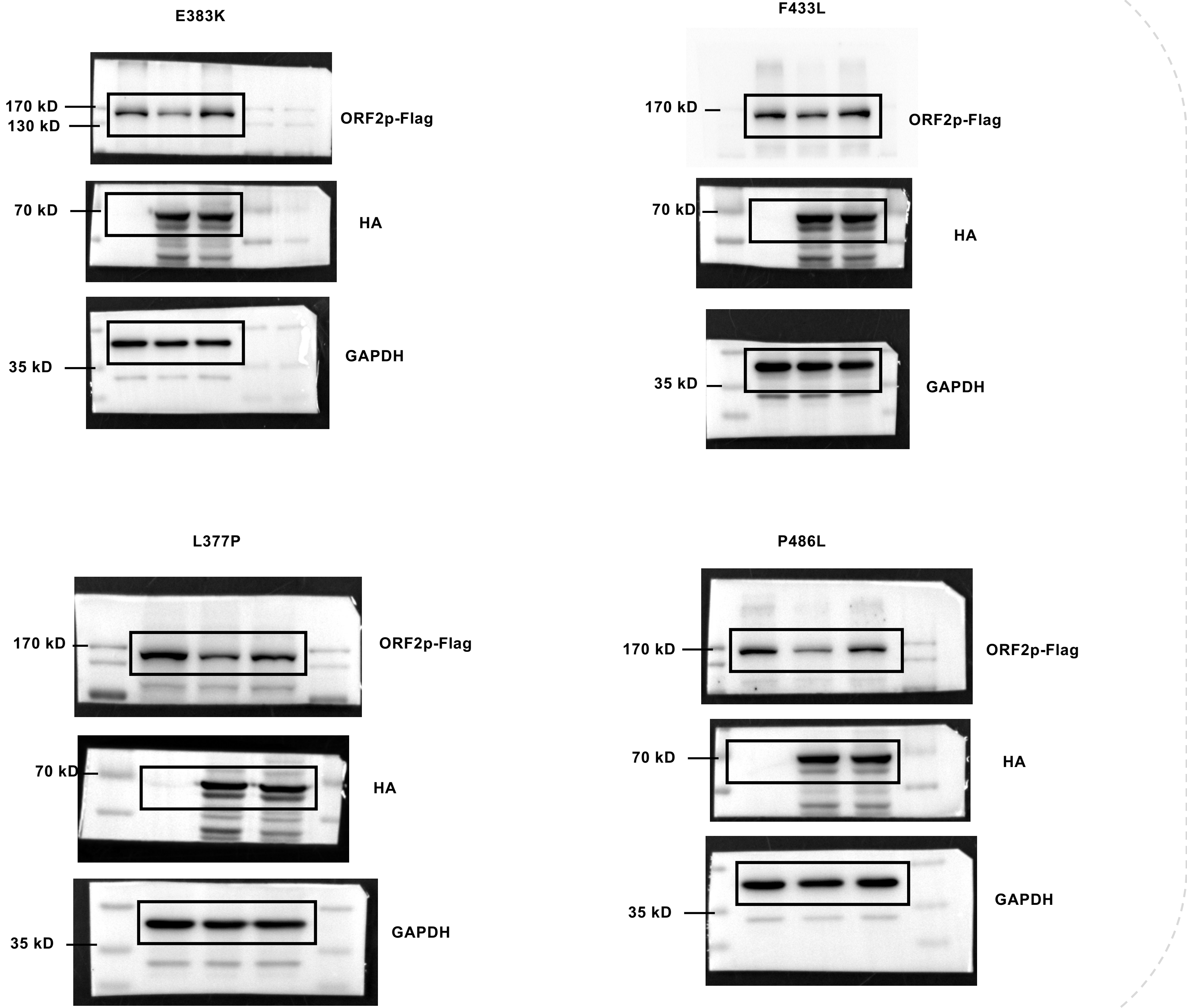
E383K
170 kD
130 kD
ORF2p-Flag
70 kD
HA
35 kD
GAPDH
F433L
170 kD
ORF2p-Flag
70 kD
HA
35 kD
GAPDH
L377P
170 kD
ORF2p-Flag
70 kD
HA
35 kD
GAPDH
P486L
170 kD
ORF2p-Flag
70 kD
HA
35 kD
GAPDH

Fig. 5d

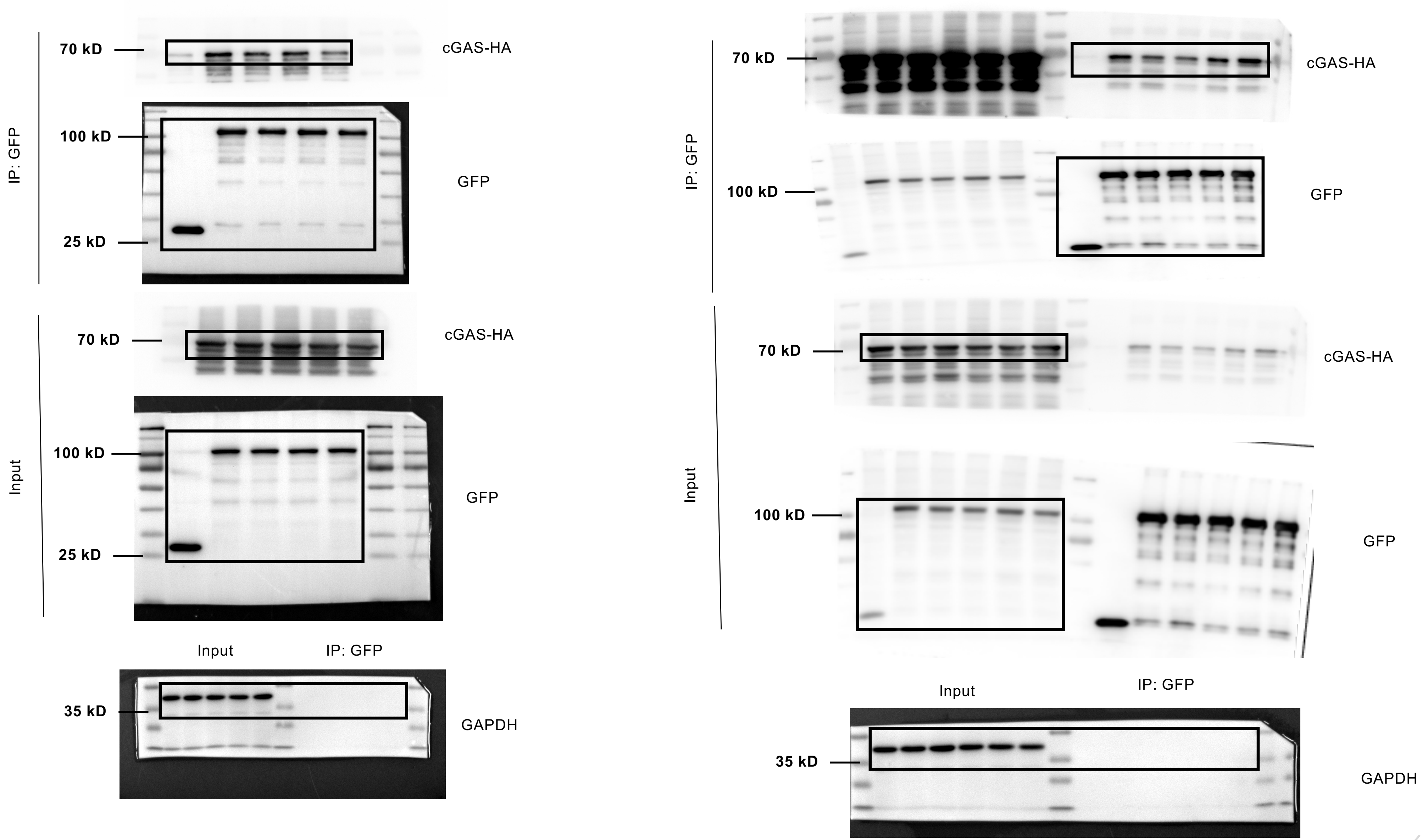
70 kD
cGAS-HA
100 kD
25 kD
GFP
IP: GFP
70 kD
cGAS-HA
100 kD
25 kD
GFP
Input
Input
IP: GFP
35 kD
GAPDH
70 kD
cGAS-HA
100 kD
GFP
IP: GFP
70 kD
cGAS-HA
100 kD
GFP
Input
Input
IP: GFP
35 kD
GAPDH

Fig. 5e

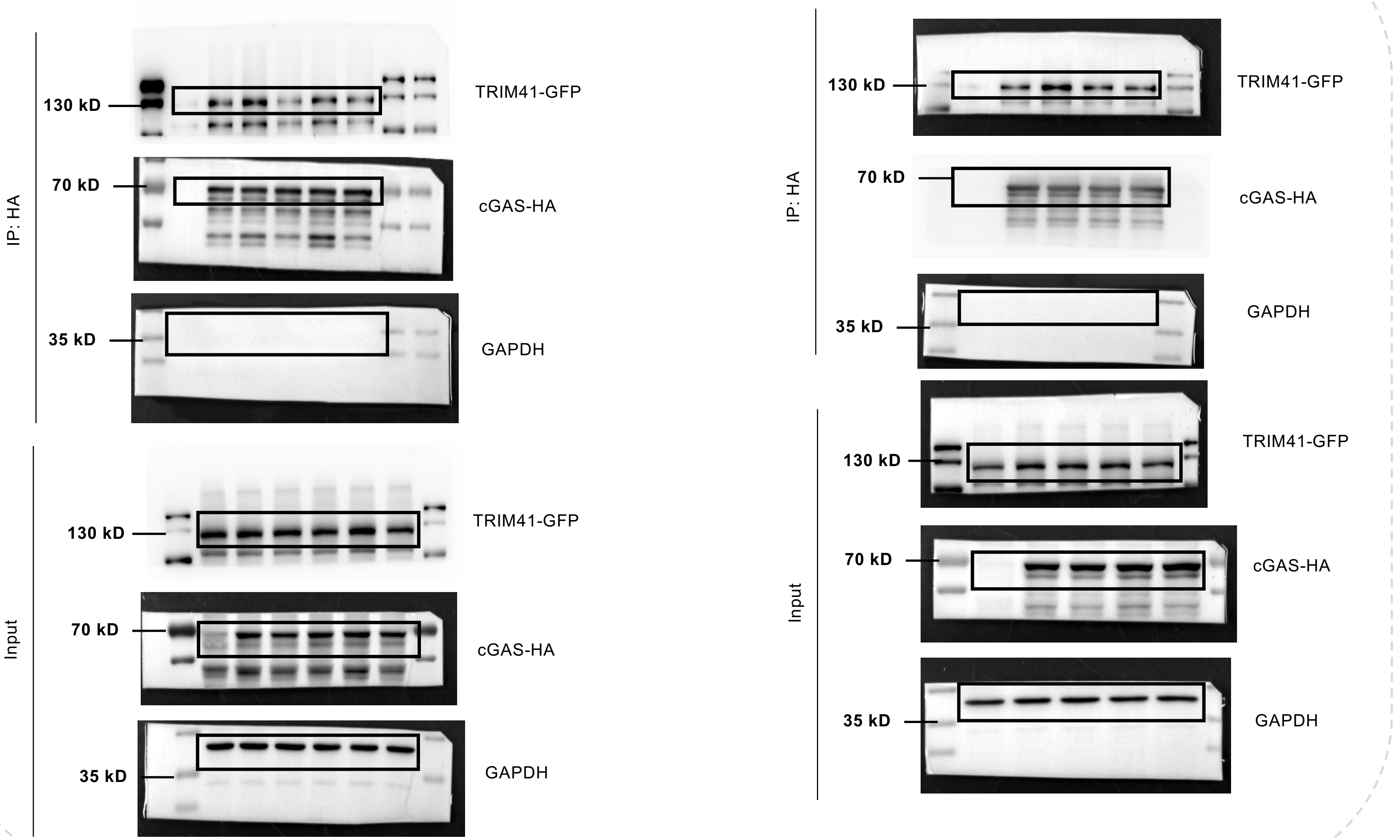

IP: HA
130 kD
TRIM41-GFP
70 kD
cGAS-HA
35 kD
GAPDH
Input
130 kD
TRIM41-GFP
70 kD
cGAS-HA
35 kD
GAPDH
IP: HA
130 kD
TRIM41-GFP
70 kD
cGAS-HA
35 kD
GAPDH
Input
130 kD
TRIM41-GFP
70 kD
cGAS-HA
35 kD
GAPDH

Fig. 5f

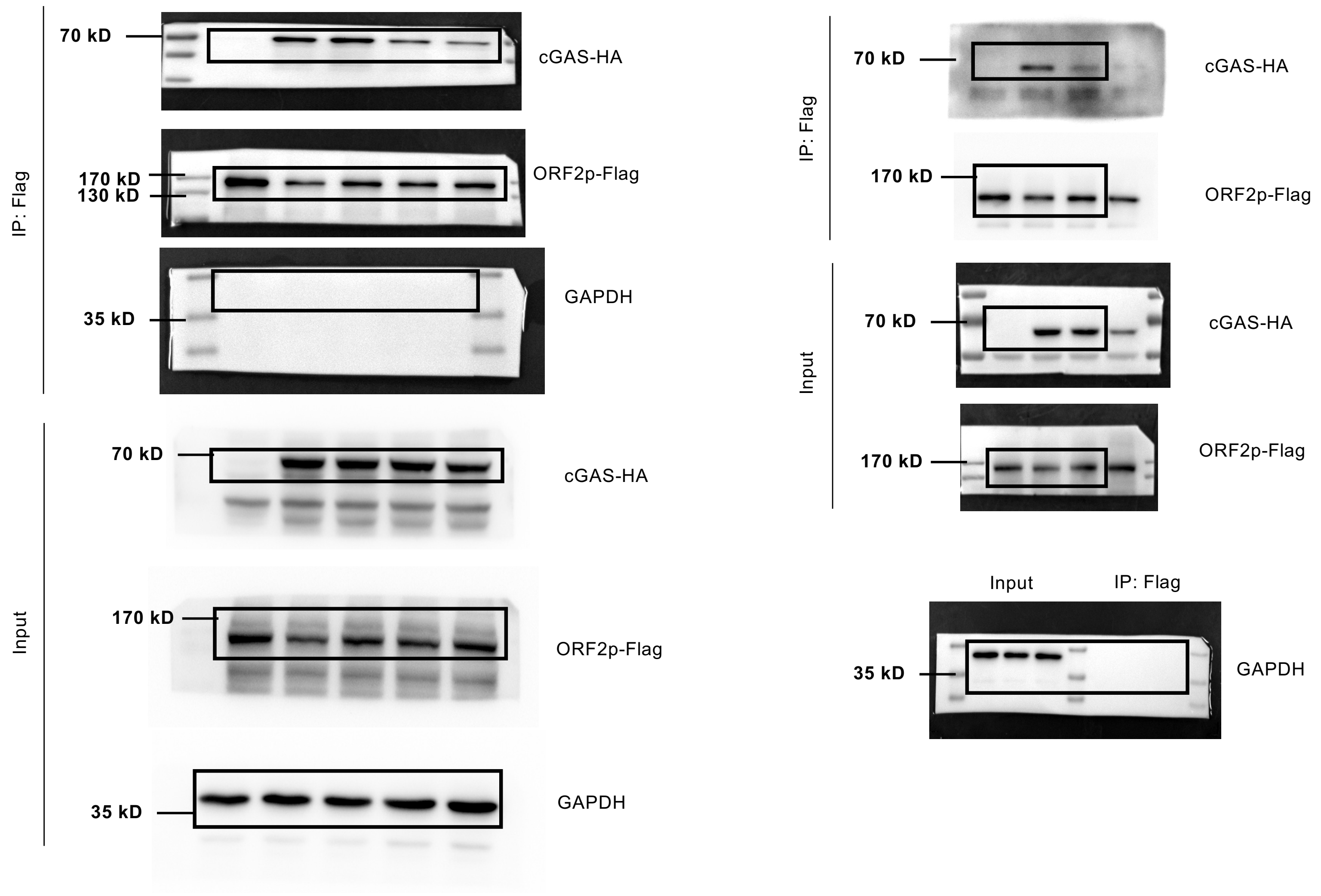
IP: Flag
Input
70 kD
cGAS-HA
170 kD
130 kD
ORF2p-Flag
35 kD
GAPDH
Input
IP: Flag

Fig. 5g

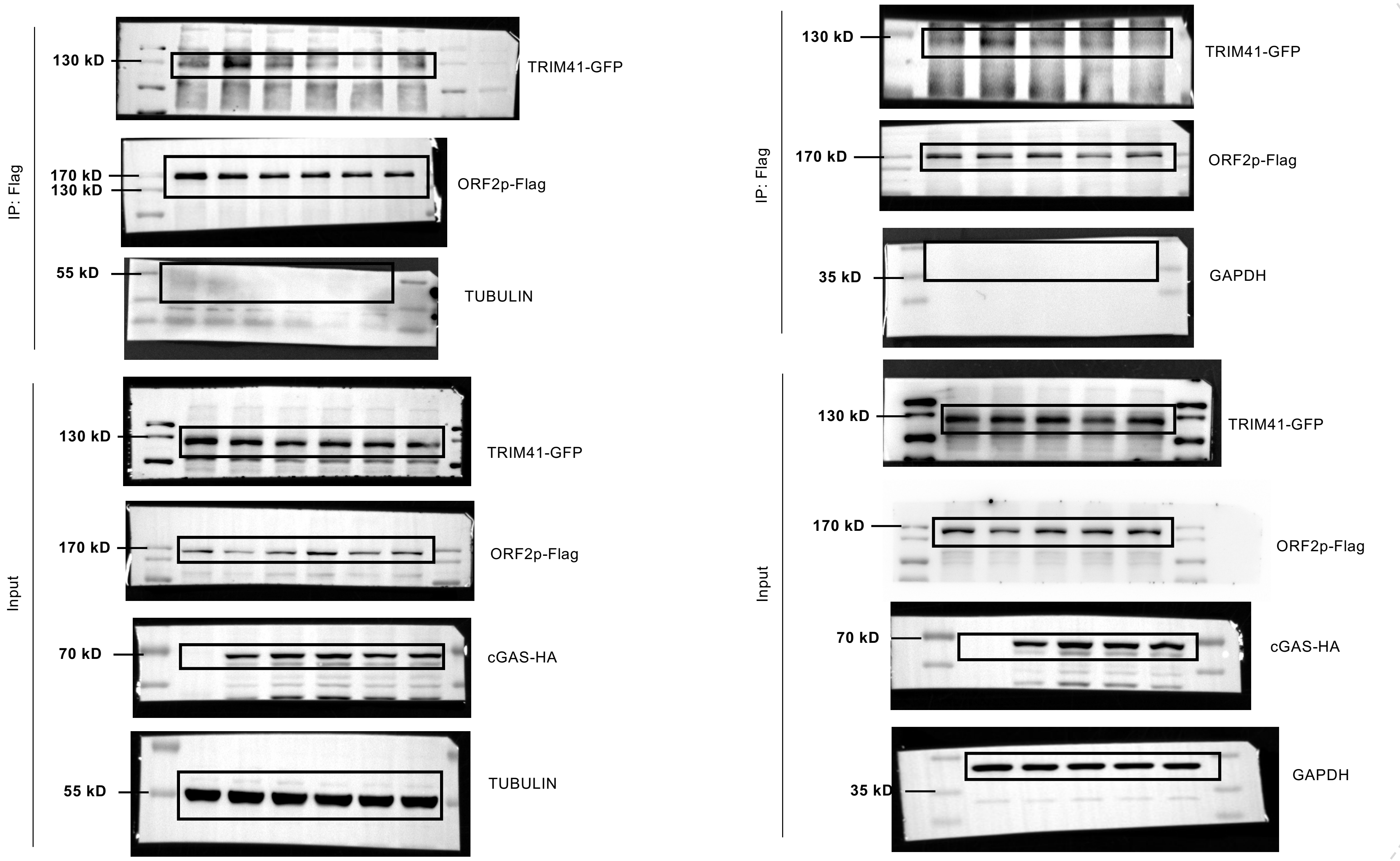

IP: Flag
130 kD
TRIM41-GFP
170 kD
130 kD
ORF2p-Flag
55 kD
TUBULIN
Input
130 kD
TRIM41-GFP
170 kD
ORF2p-Flag
70 kD
cGAS-HA
55 kD
TUBULIN
IP: Flag
130 kD
TRIM41-GFP
170 kD
ORF2p-Flag
35 kD
GAPDH
Input
130 kD
TRIM41-GFP
170 kD
ORF2p-Flag
70 kD
cGAS-HA
35 kD
GAPDH

Supplementary Fig. 1b

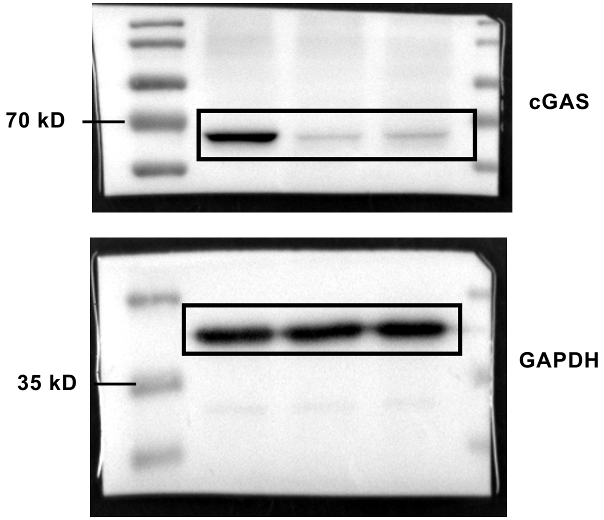

Supplementary Fig. 1c

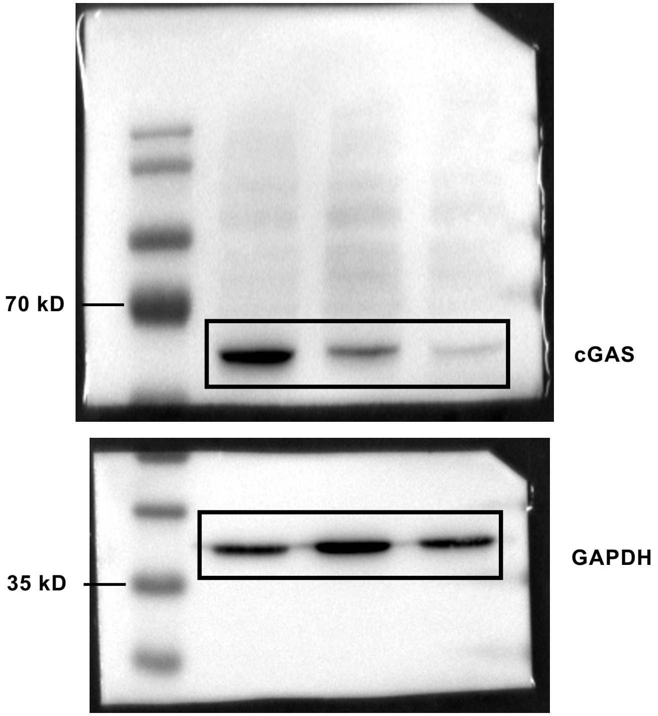

Supplementary Fig. 1e

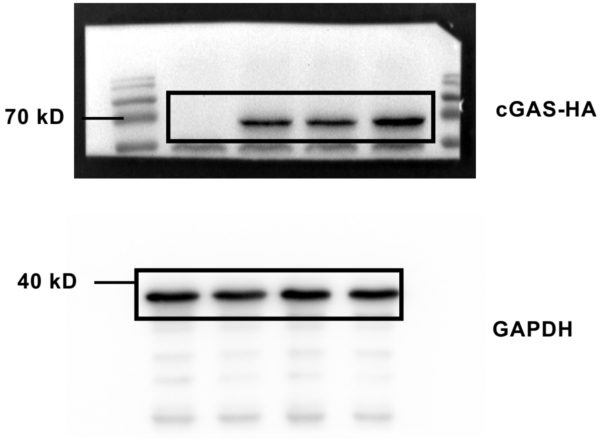

Supplementary Fig. 1f

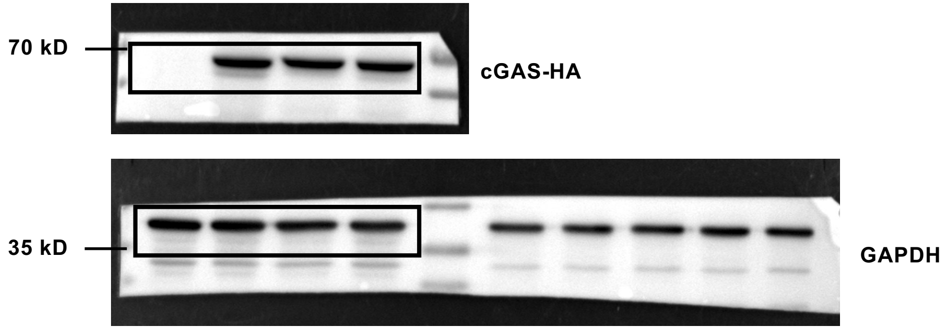

Supplementary Fig. 1g

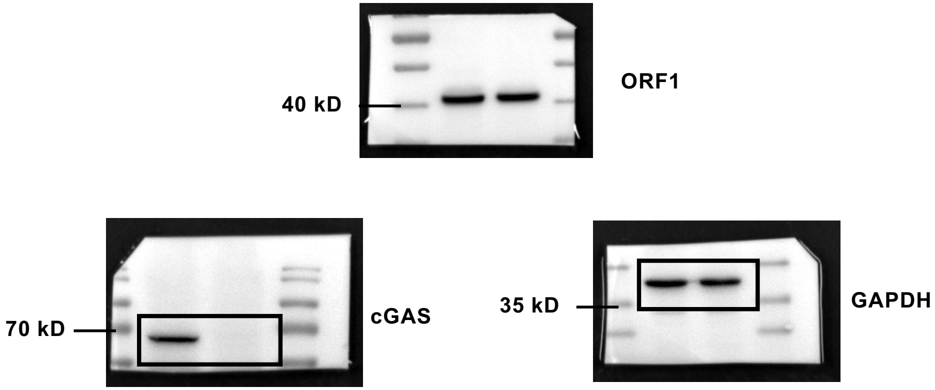

Supplementary Fig. 1h

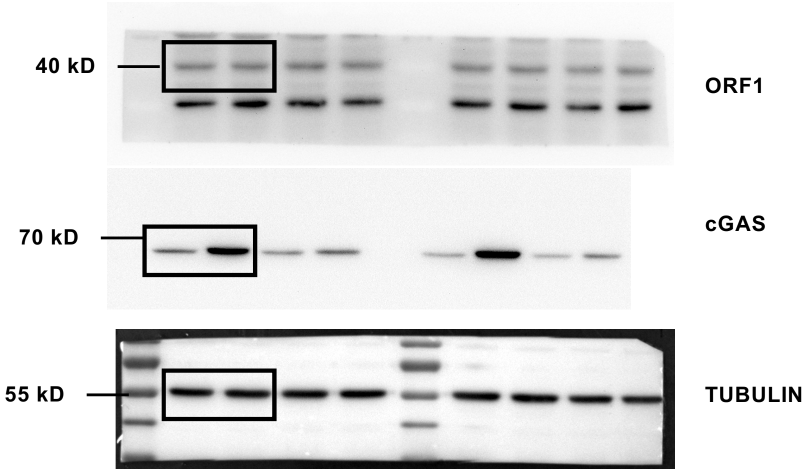

Supplementary Fig. 1j

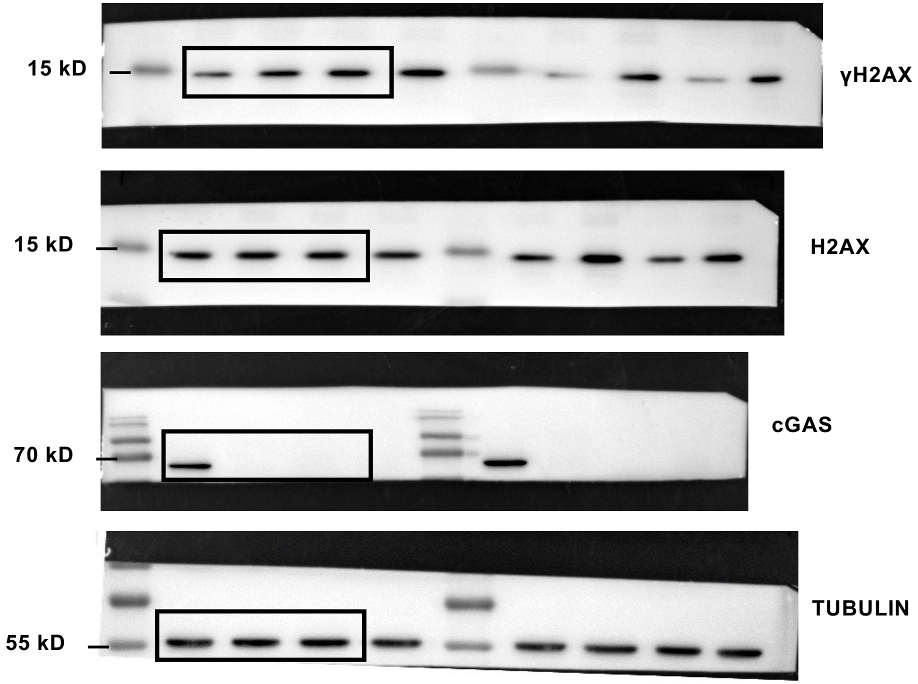

Supplementary Fig. 1i

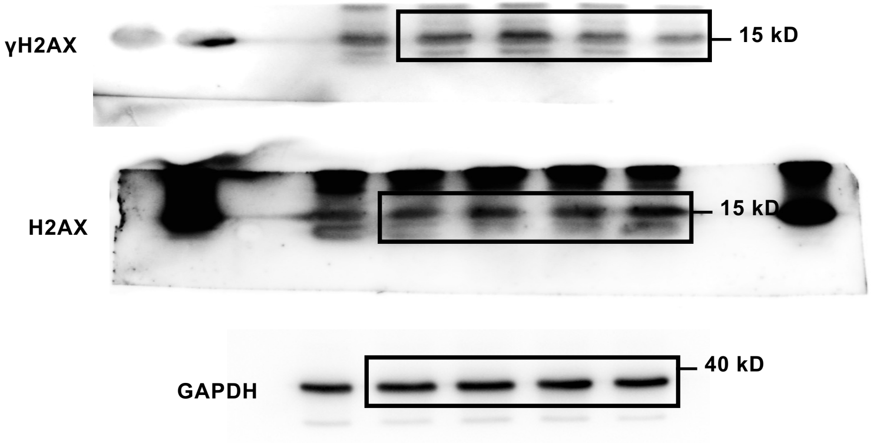

Supplementary Fig. 2a

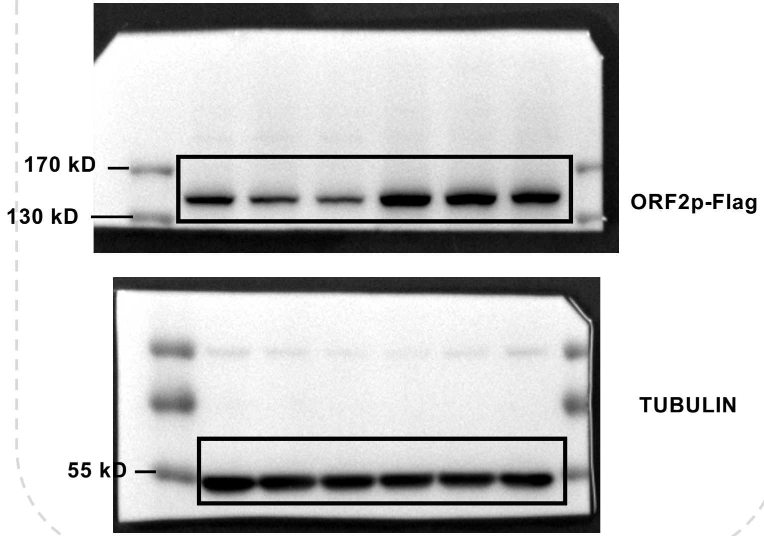

Supplementary Fig. 2b

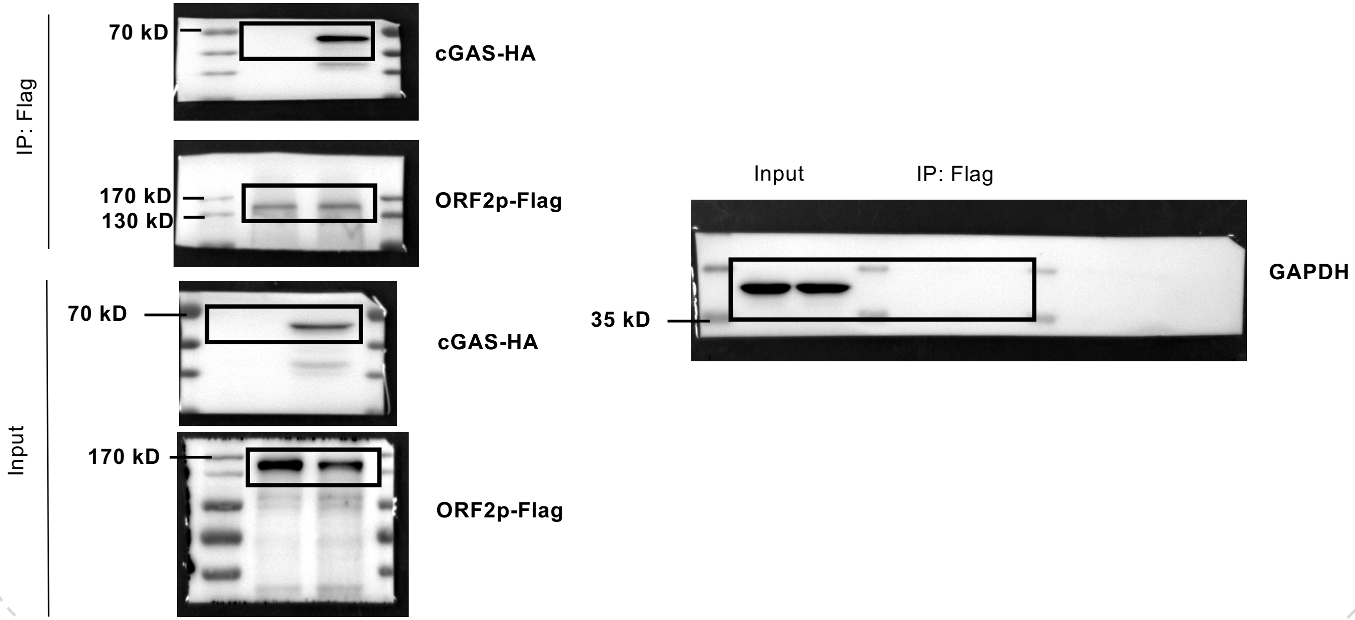

Supplementary Fig. 2c

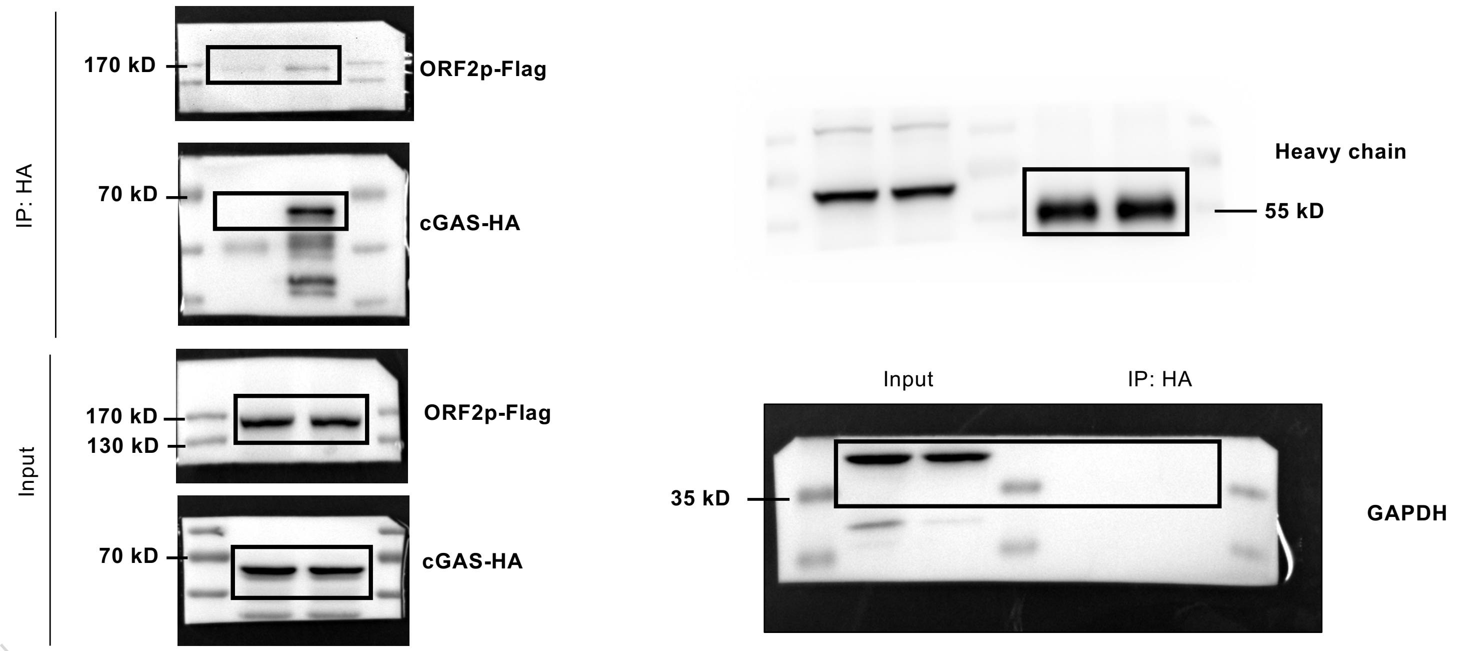

Supplementary Fig. 2d

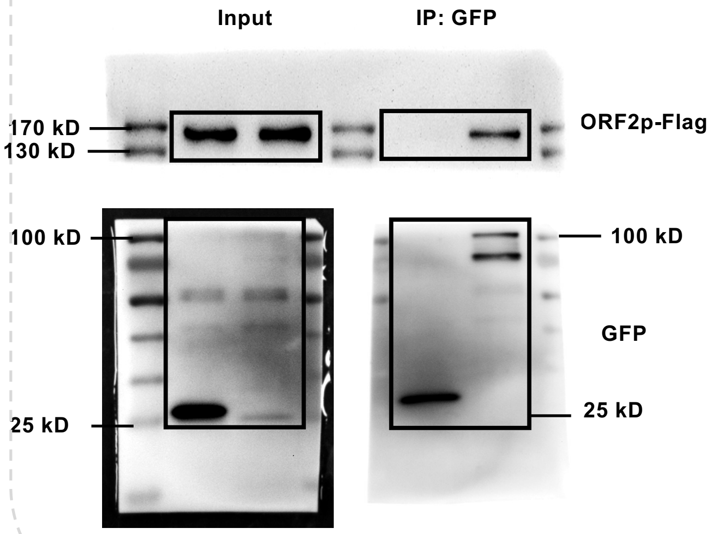

Supplementary Fig. 2g

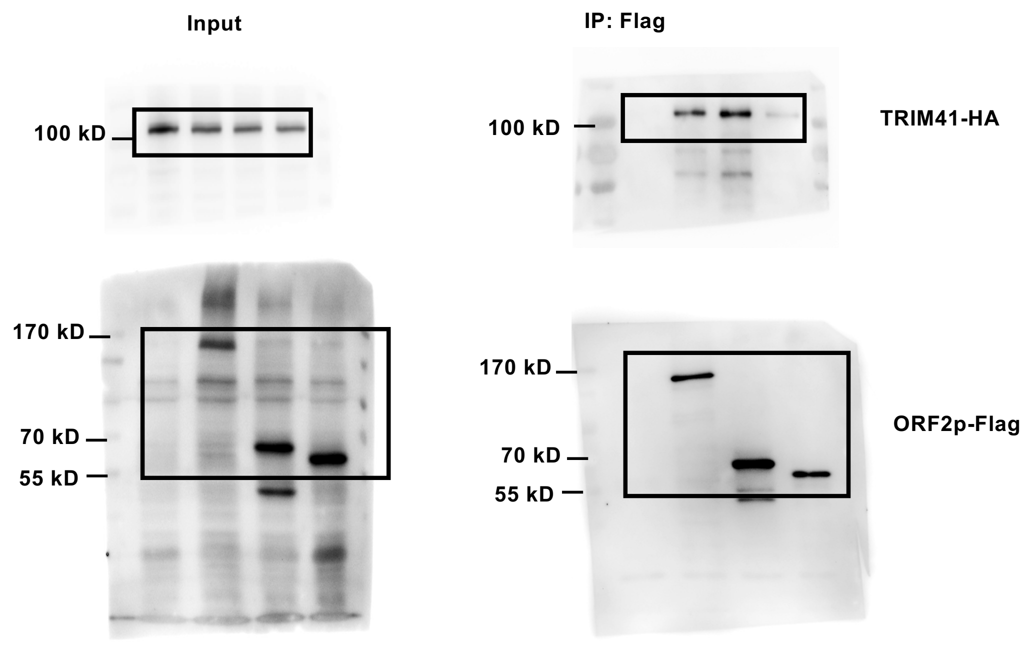

Supplementary Fig. 2h

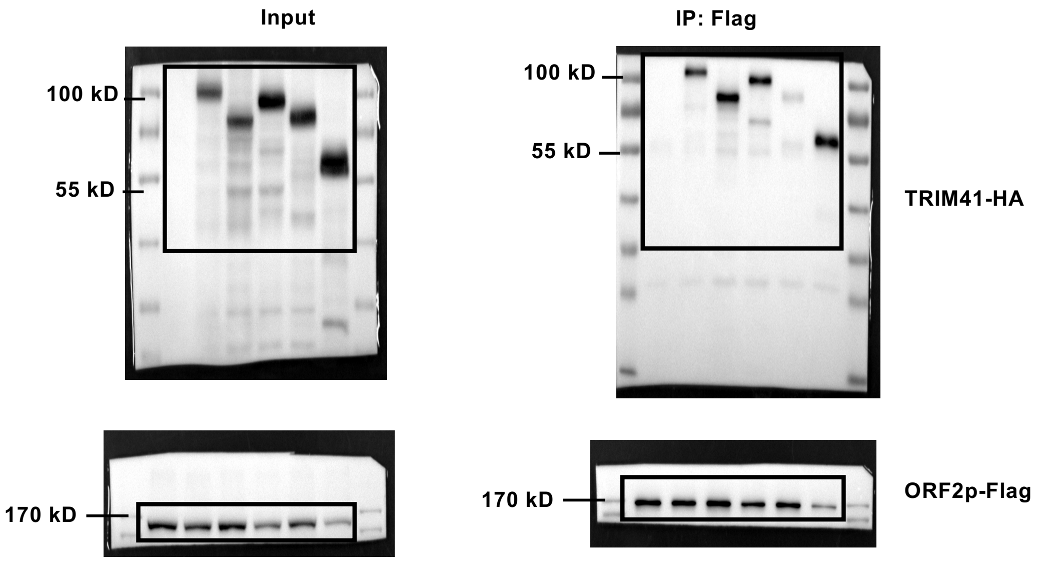

Supplementary Fig. 2i

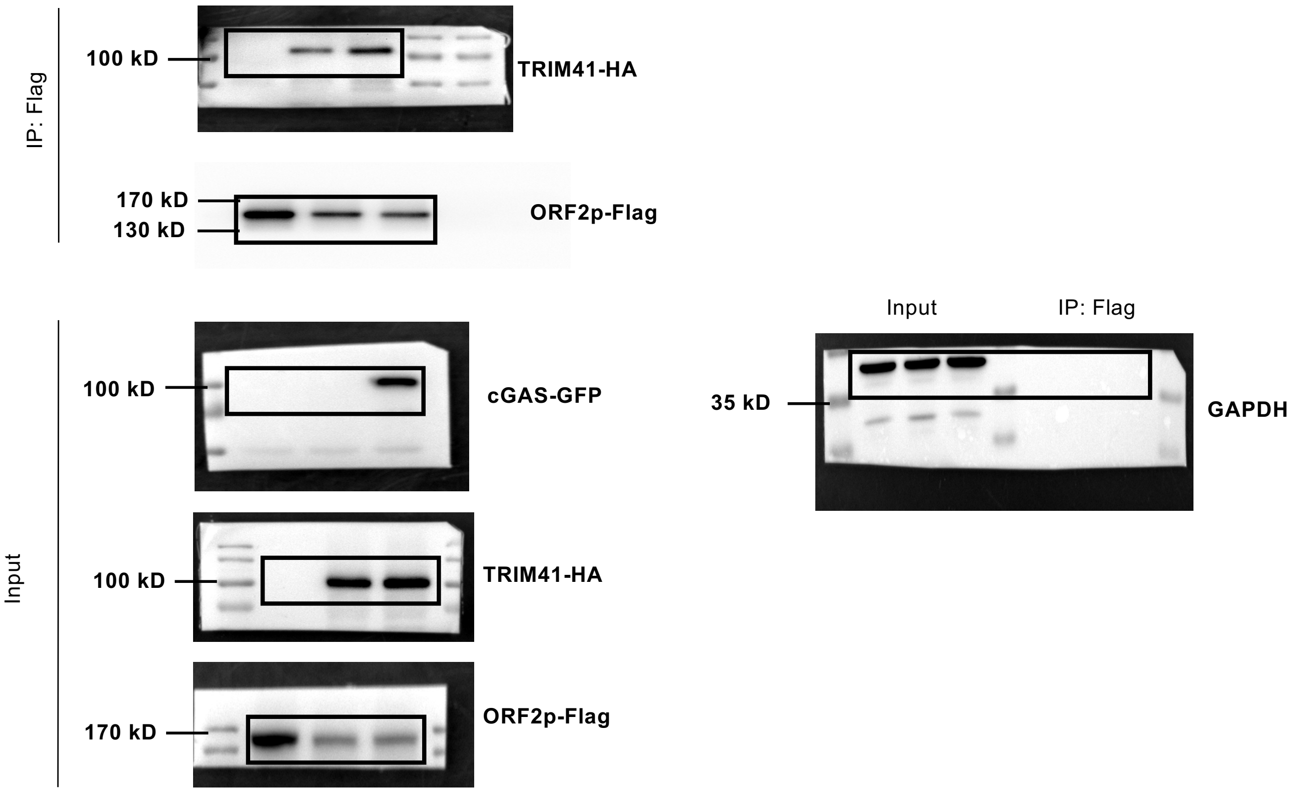

Supplementary Fig. 2j

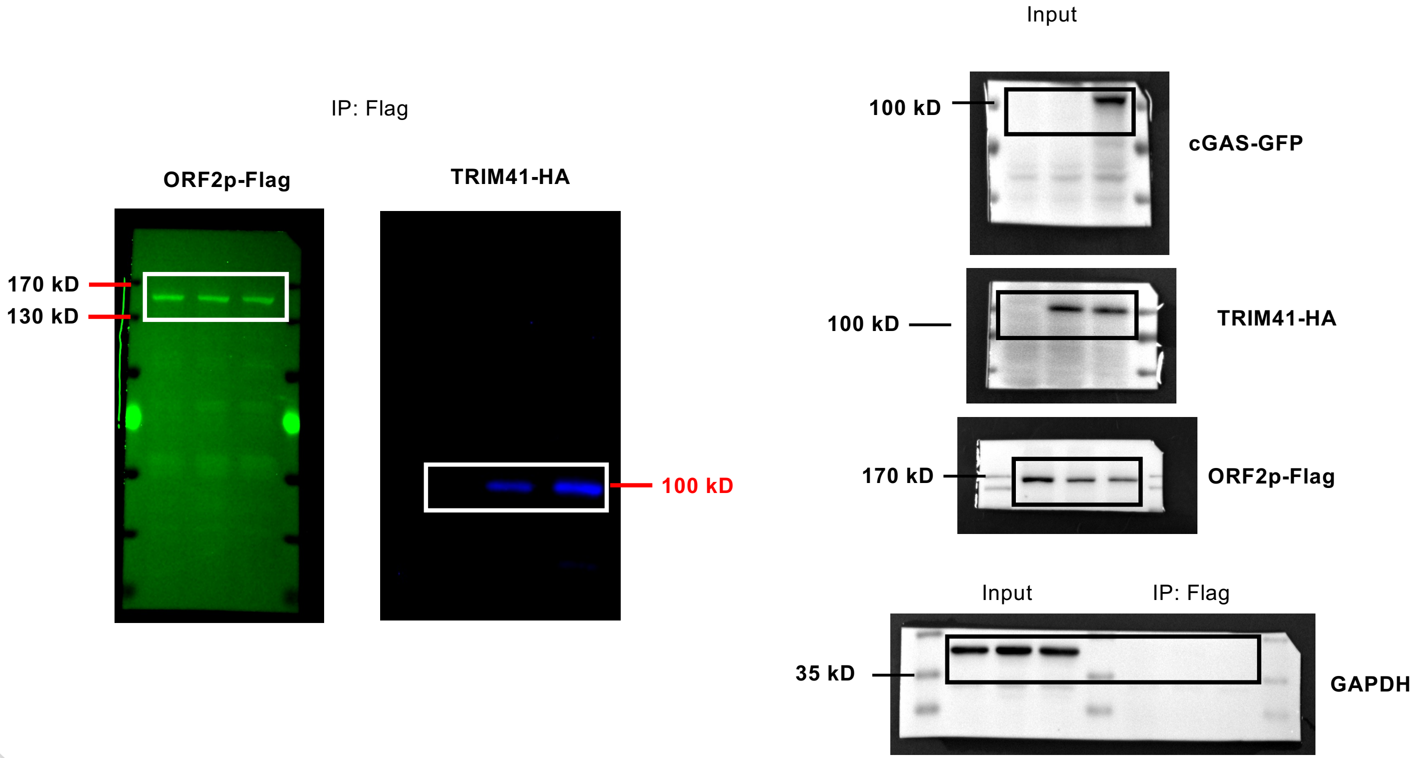

Supplementary Fig. 2l

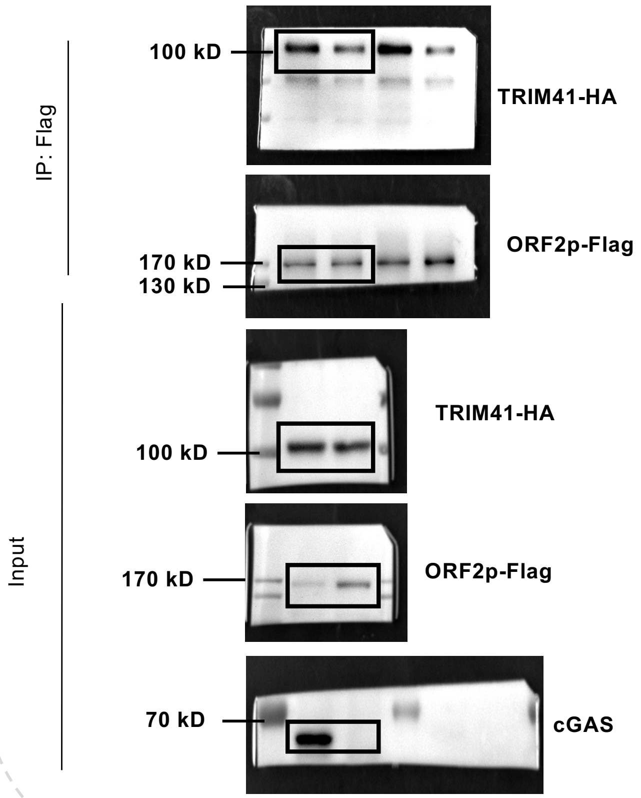

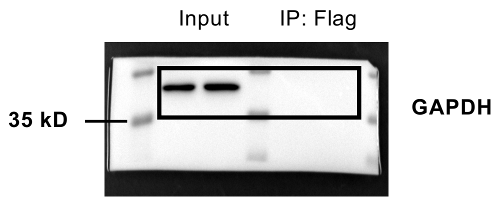

Supplementary Fig. 2m

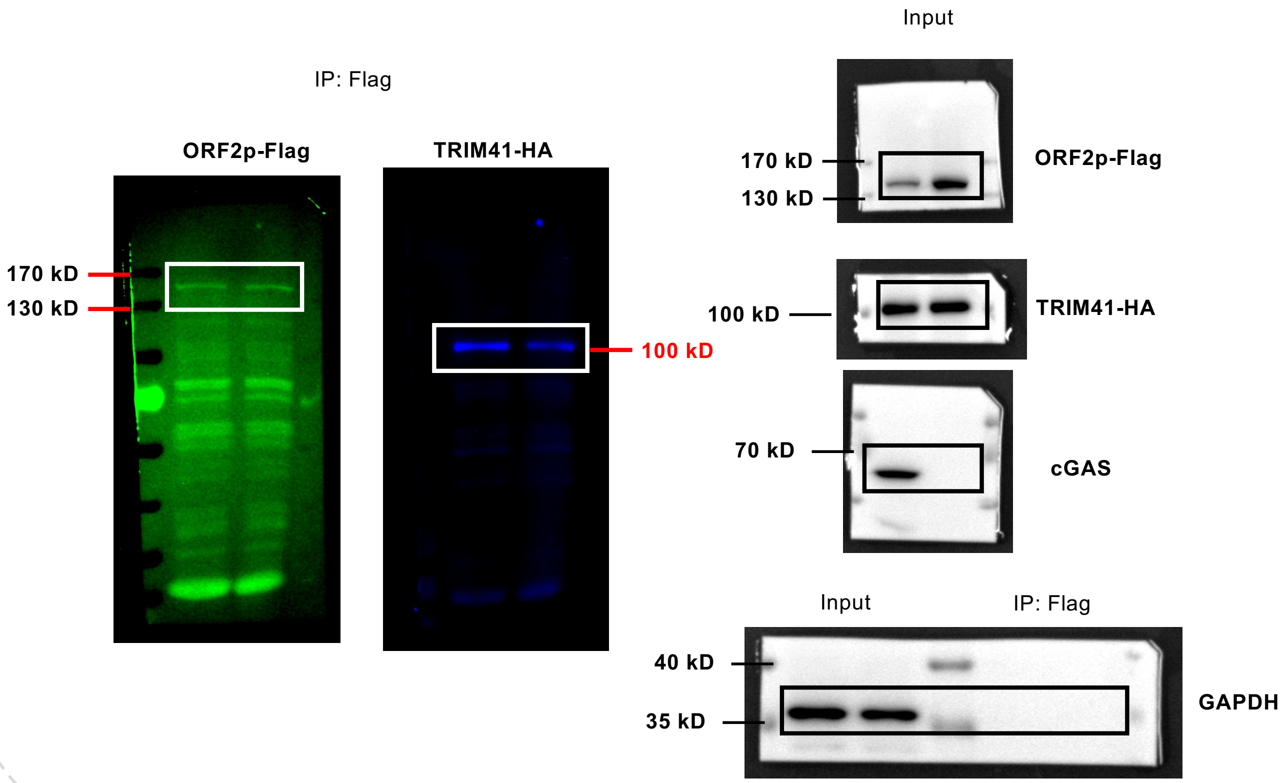

Supplementary Fig. 2n

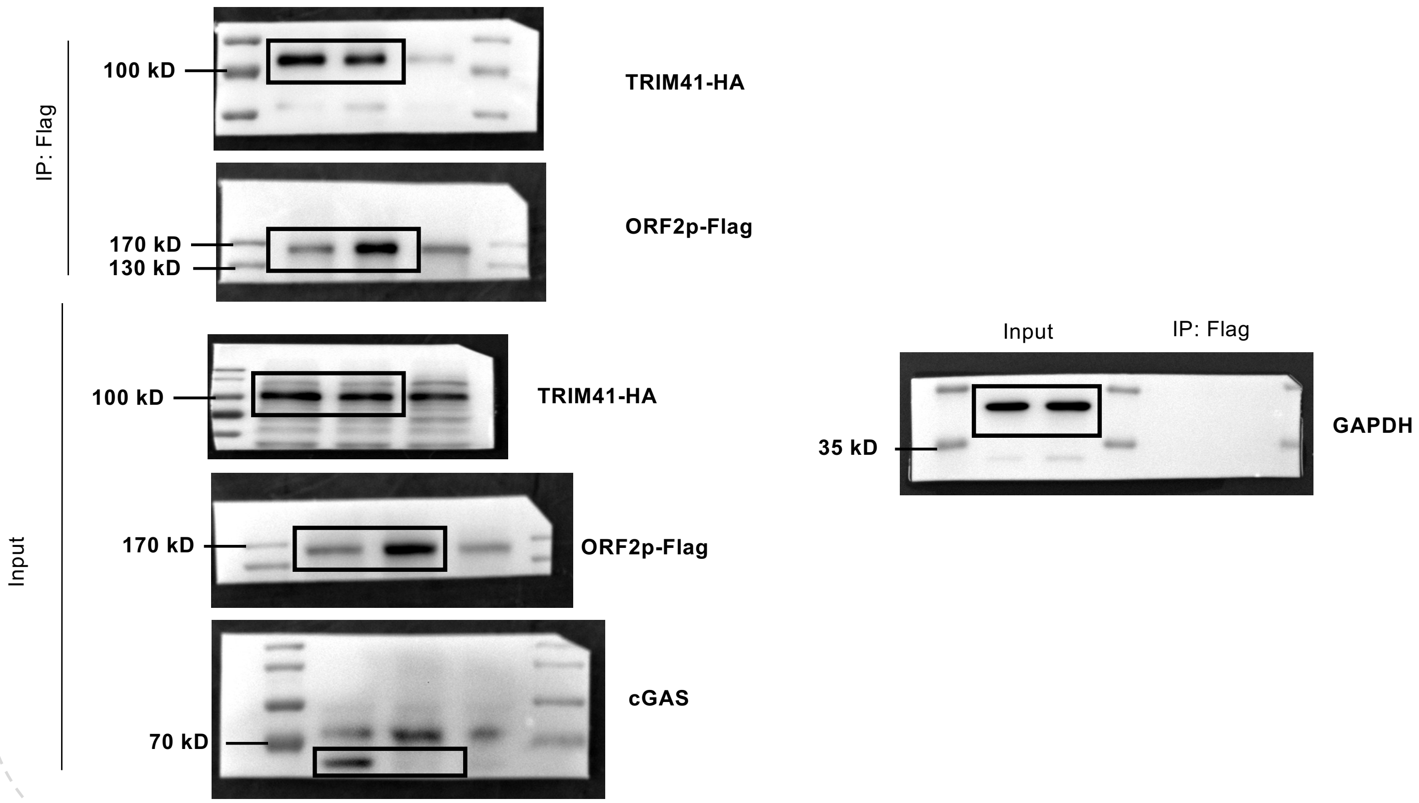

Supplementary Fig. 2o

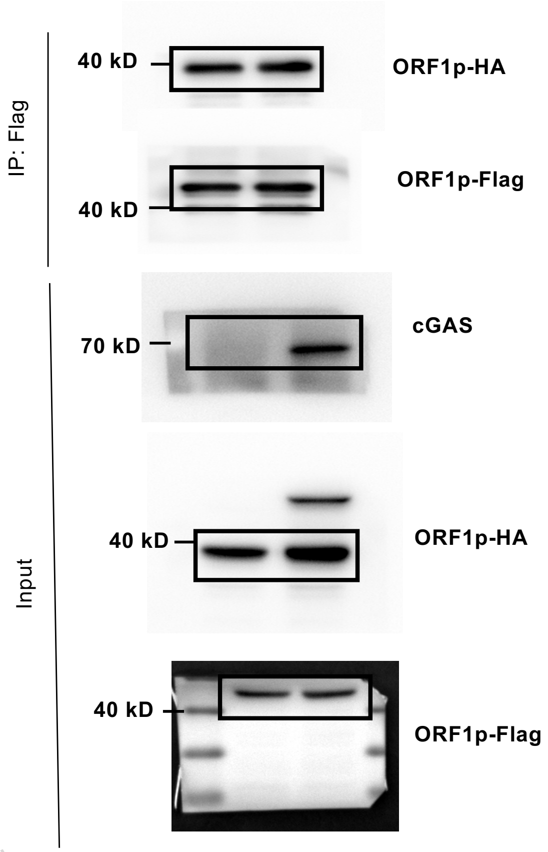

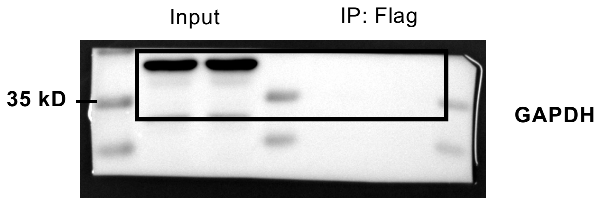

Supplementary Fig. 2p

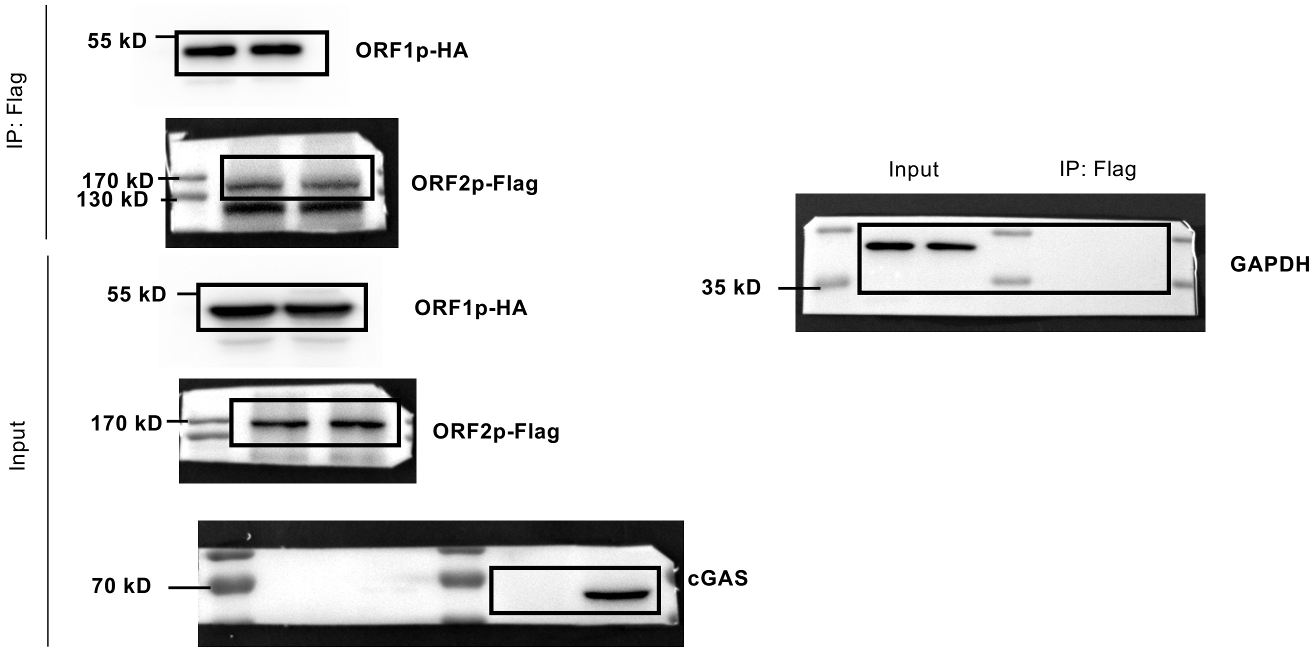

Supplementary Fig. 3a

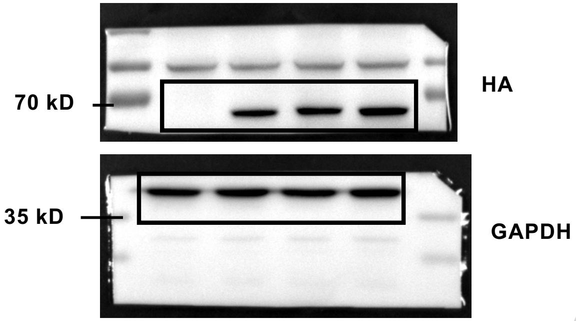

Supplementary Fig. 3c

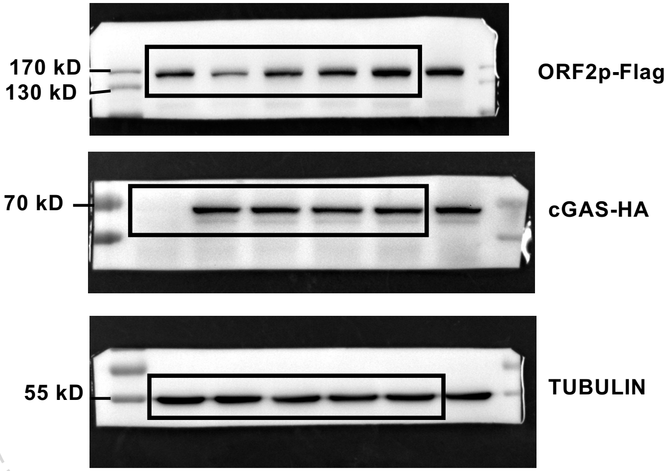

Supplementary Fig. 3d

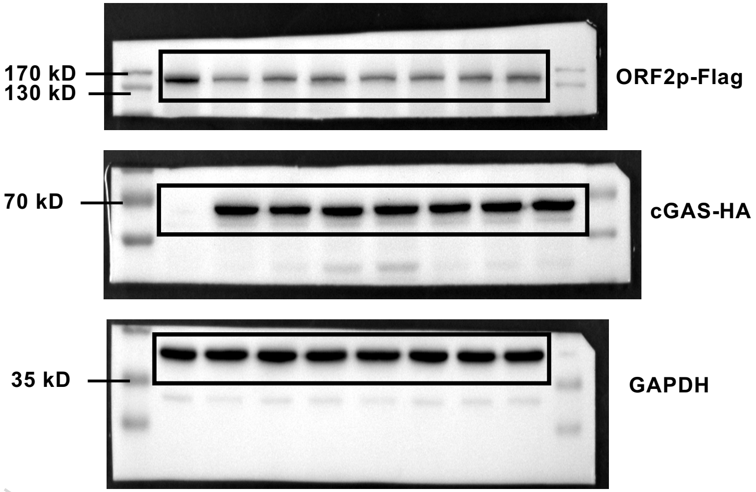

Supplementary Fig. 3f

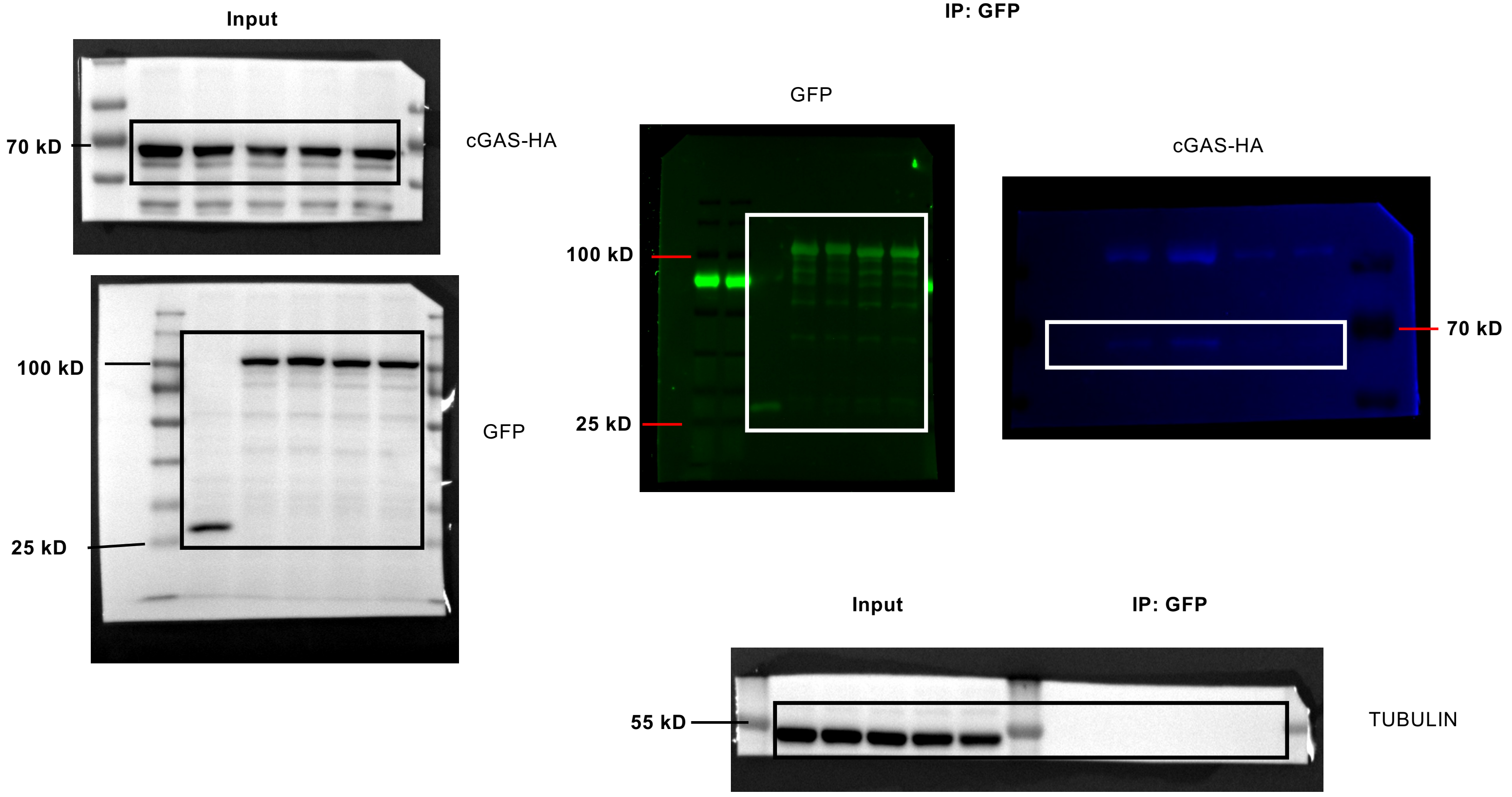

Supplementary Fig. 3g

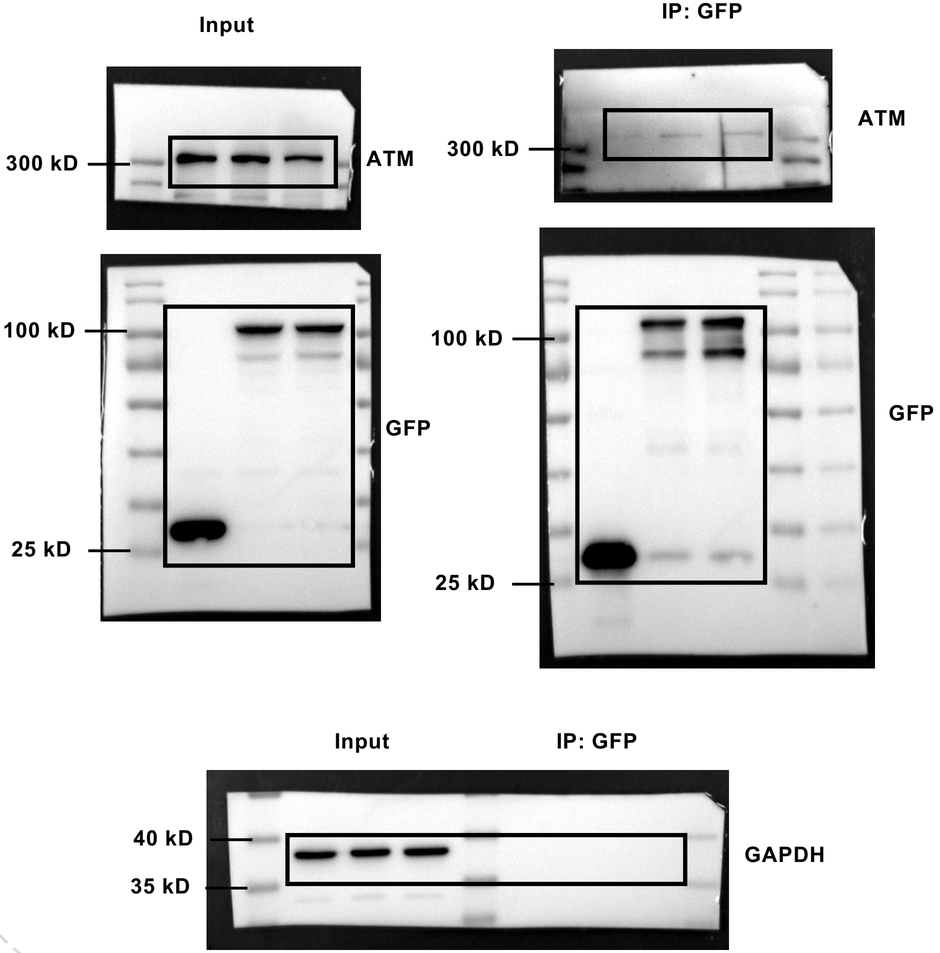

Supplementary Fig. 3h

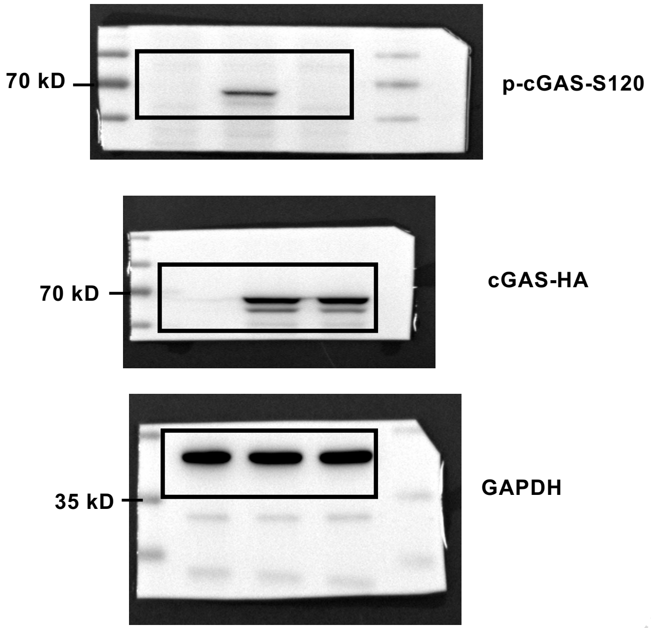

Supplementary Fig. 3i

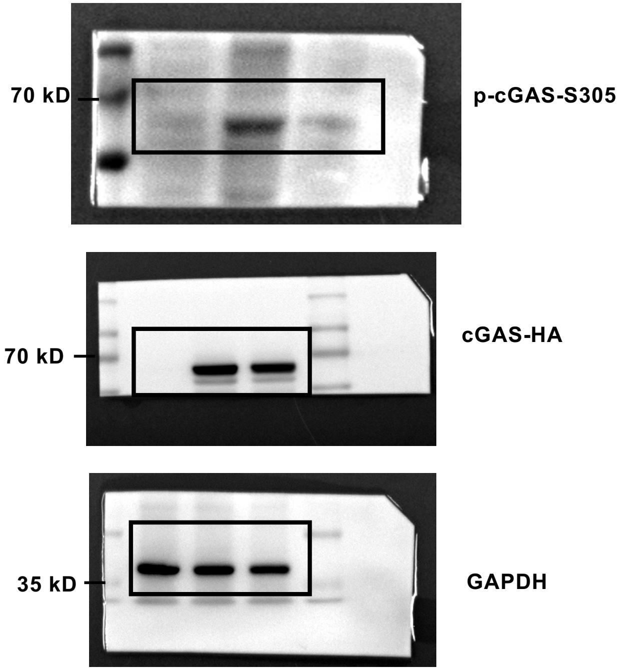

Supplementary Fig. 3j

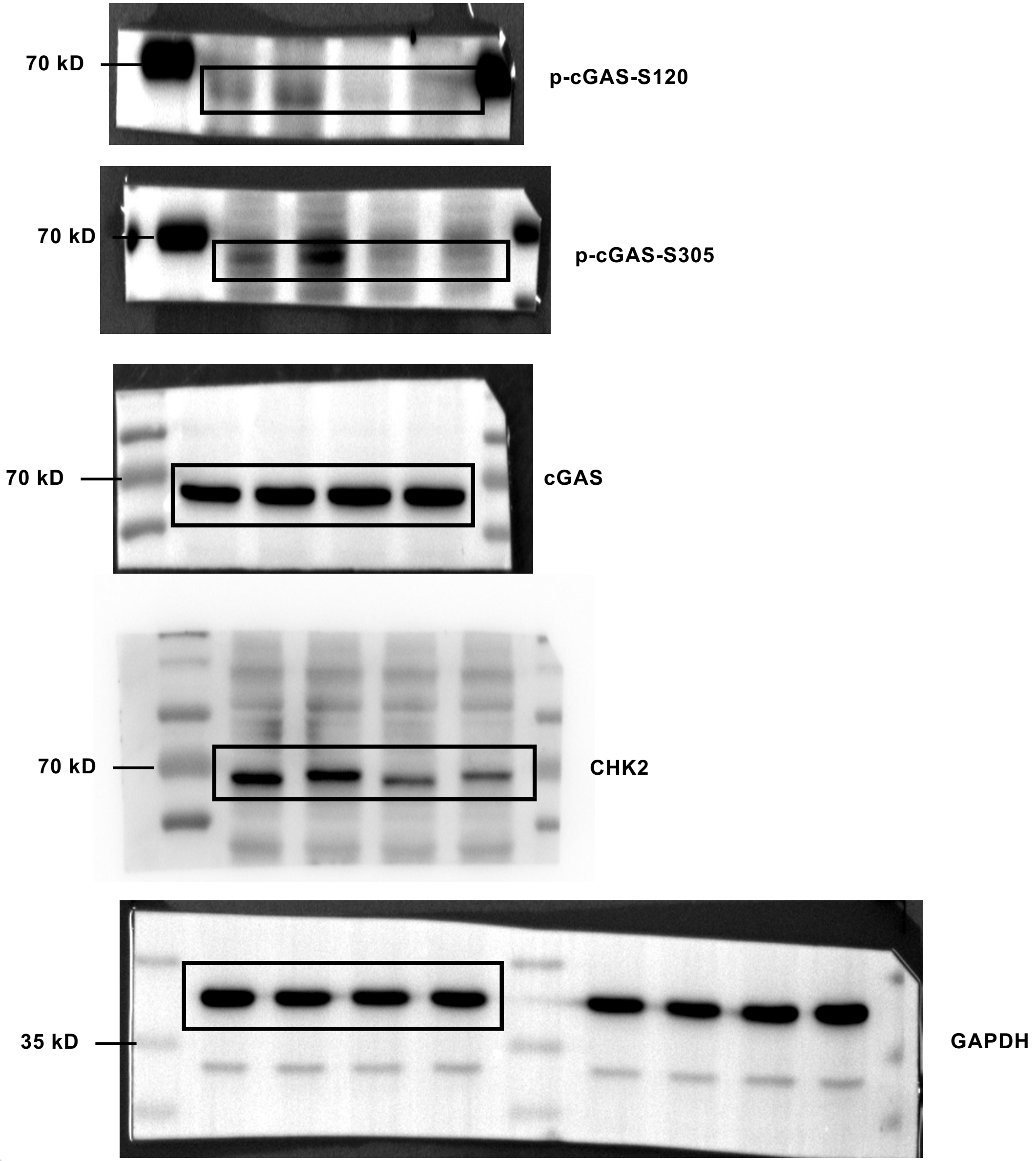

Supplementary Fig. 4a

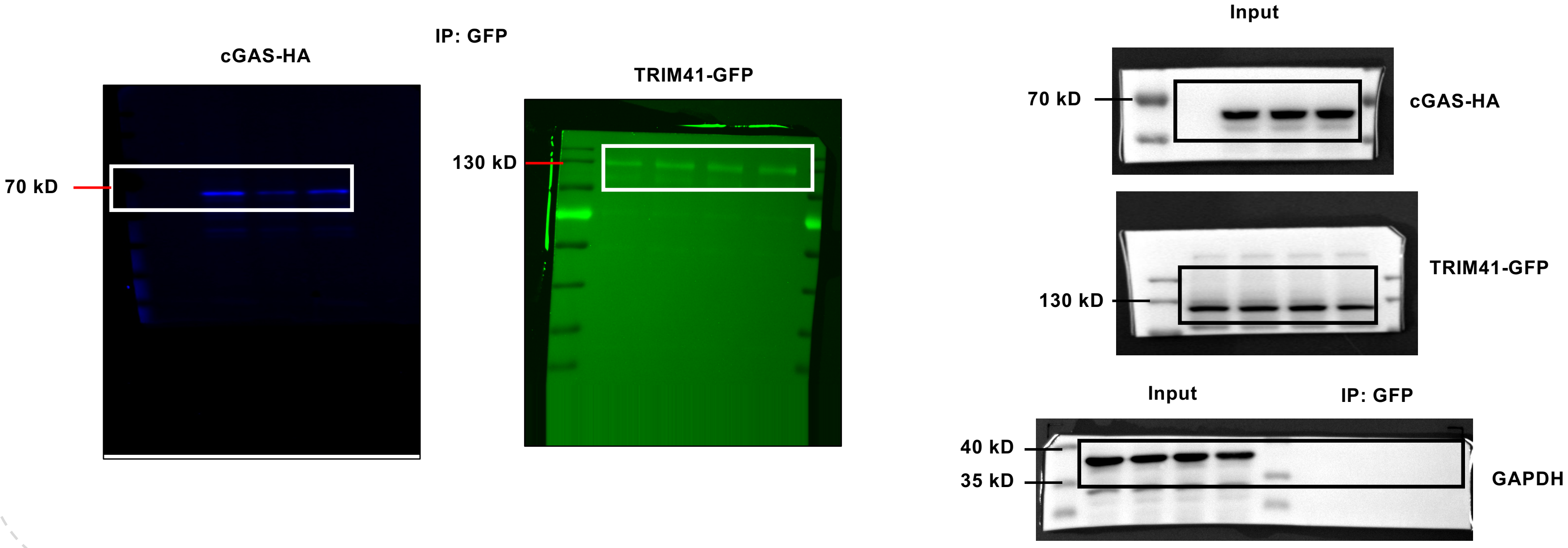

Supplementary Fig. 4c

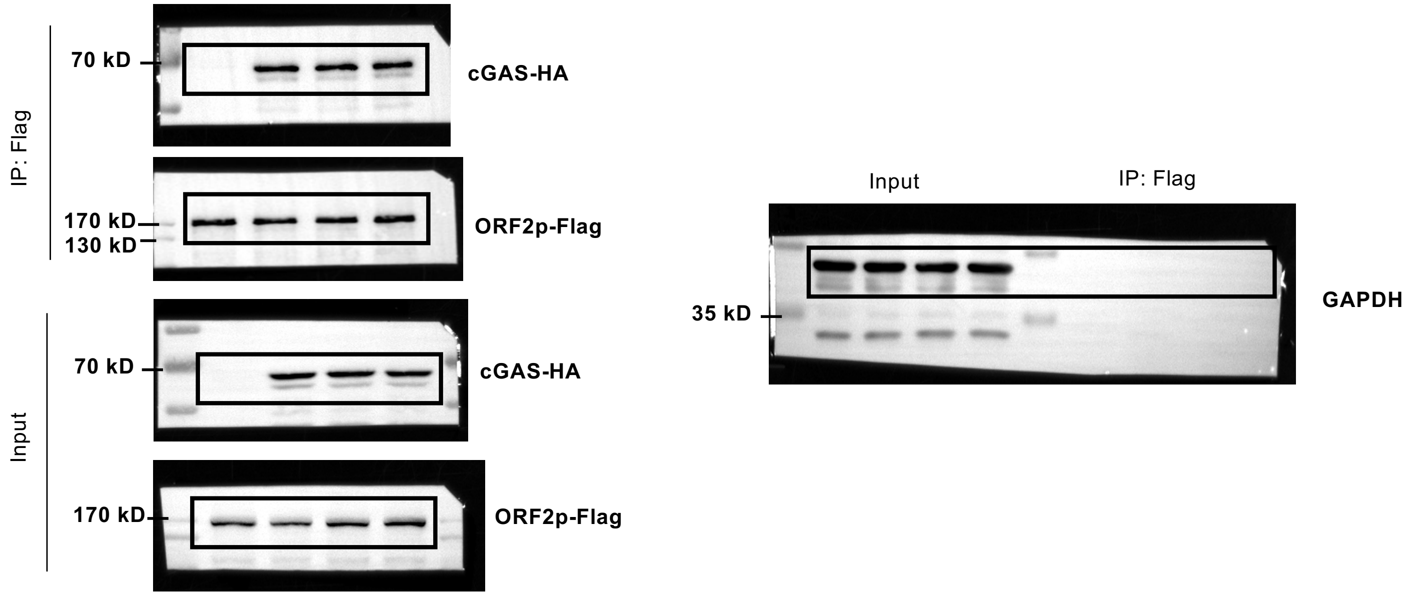

Supplementary Fig. 4d

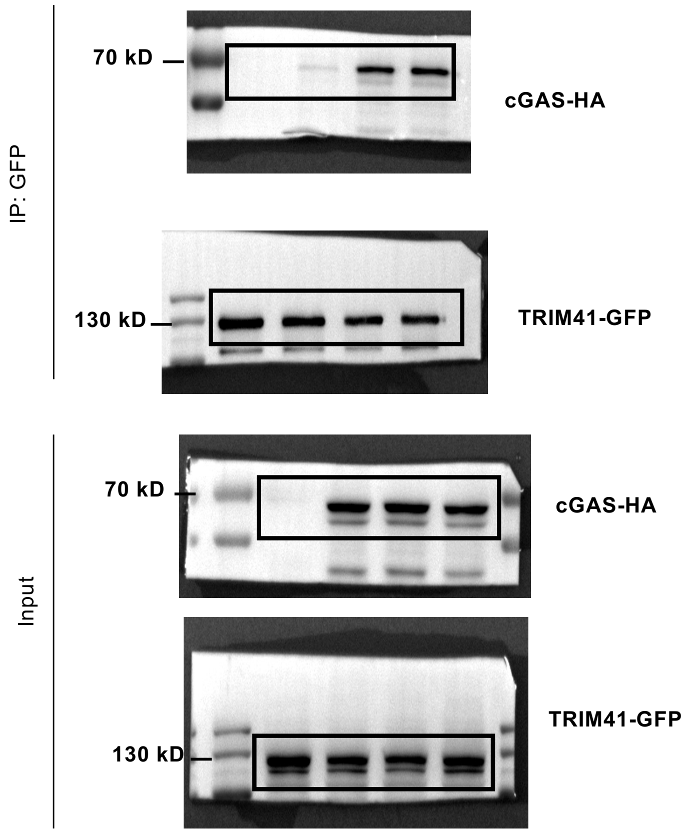

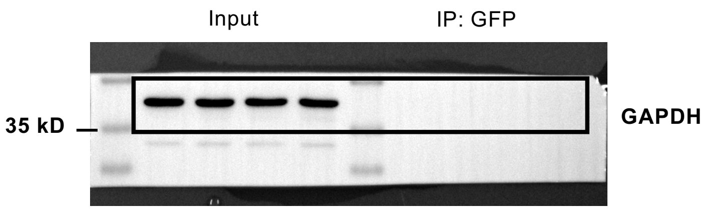

Supplementary Fig. 4e

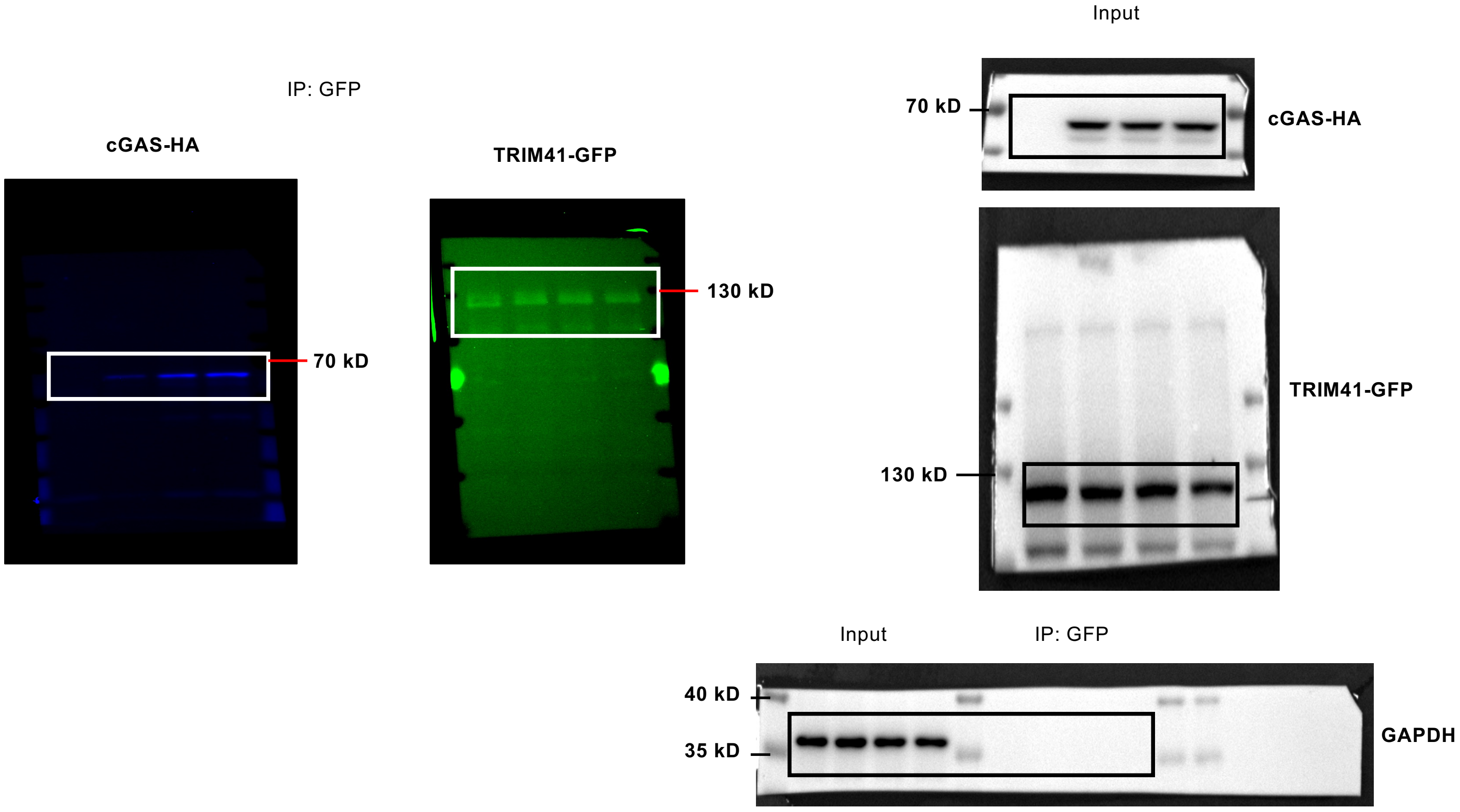

Supplementary Fig. 4f

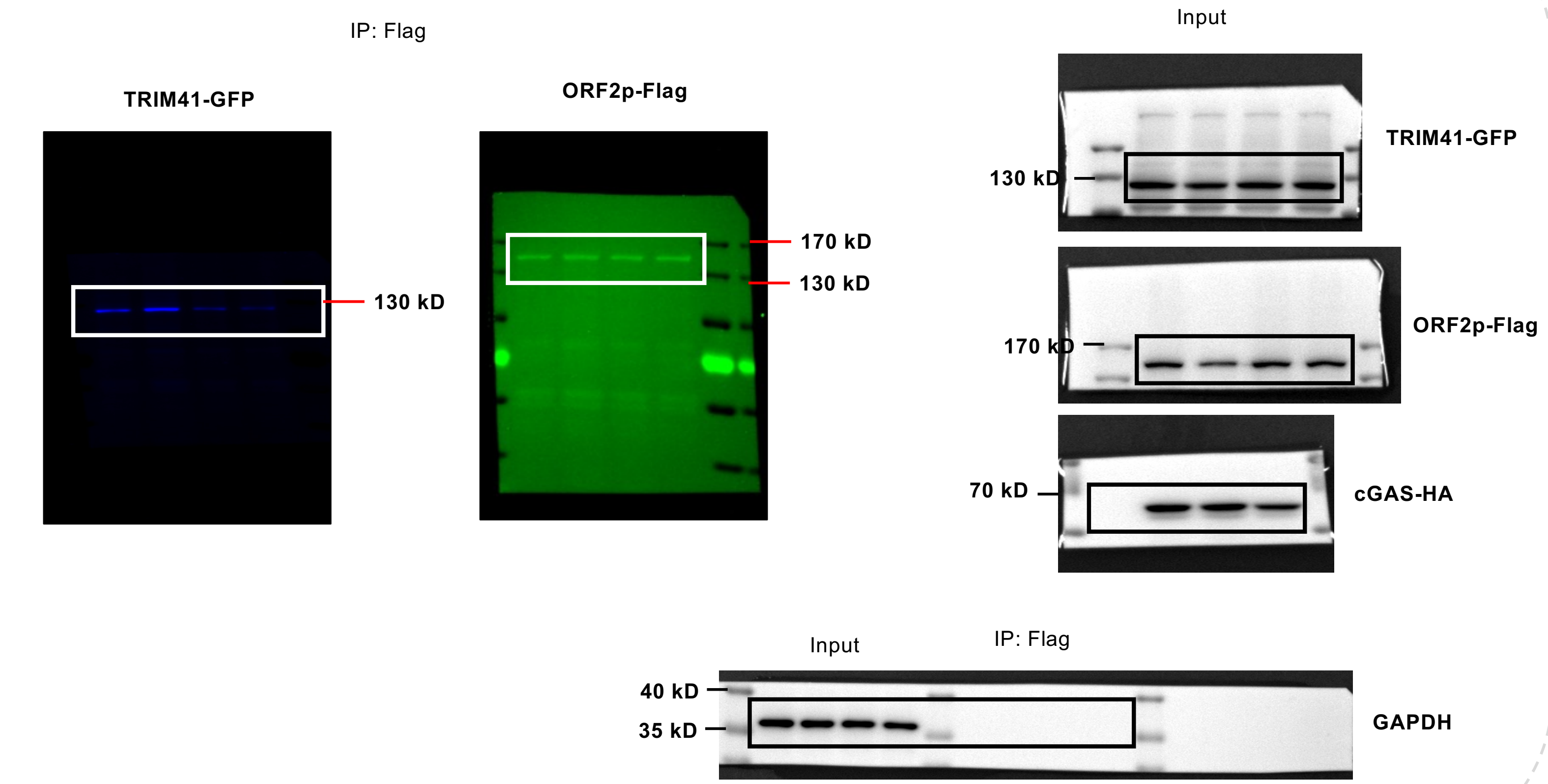

Supplementary Fig. 5a

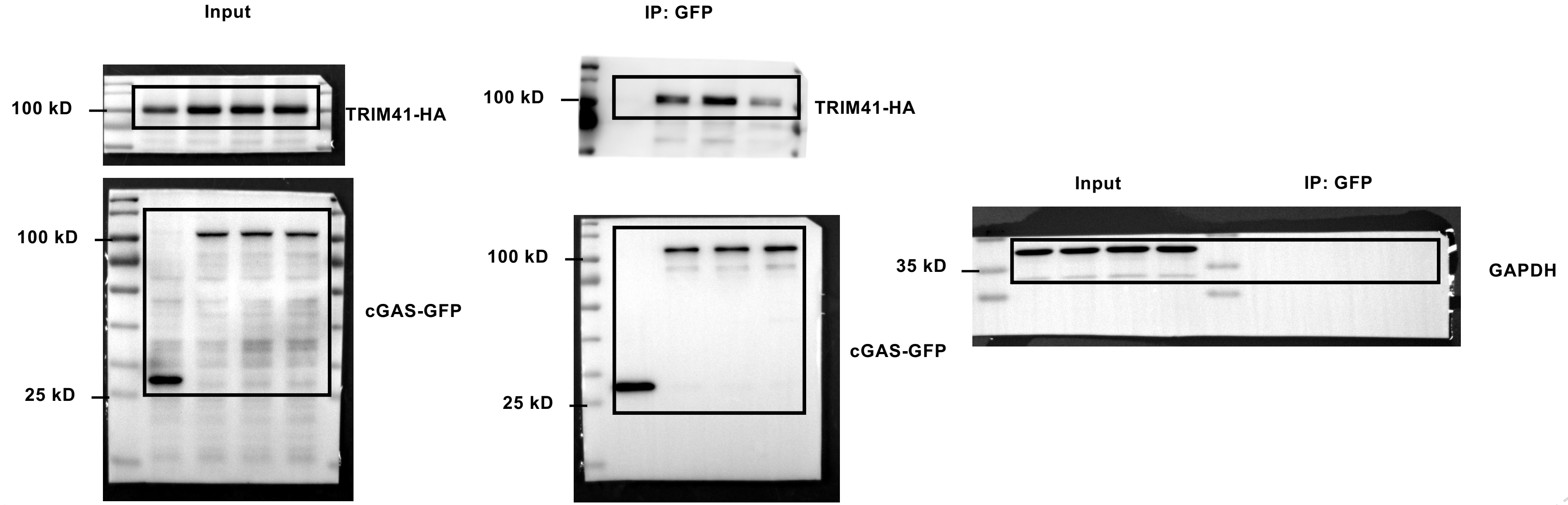

Supplementary Fig. 5b

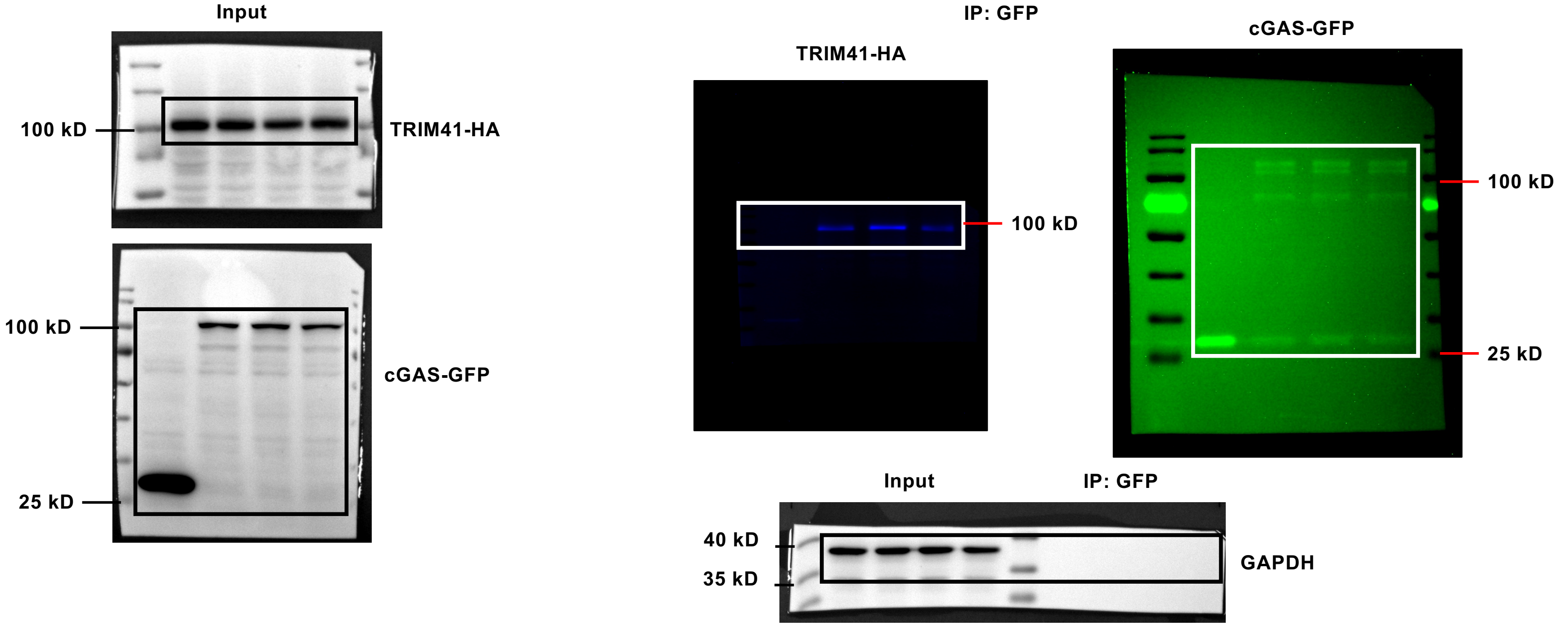

Supplementary Fig. 5d

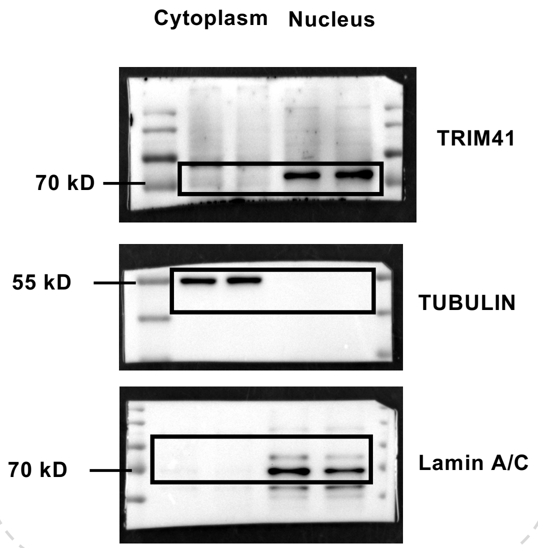

Supplementary Fig. 5f

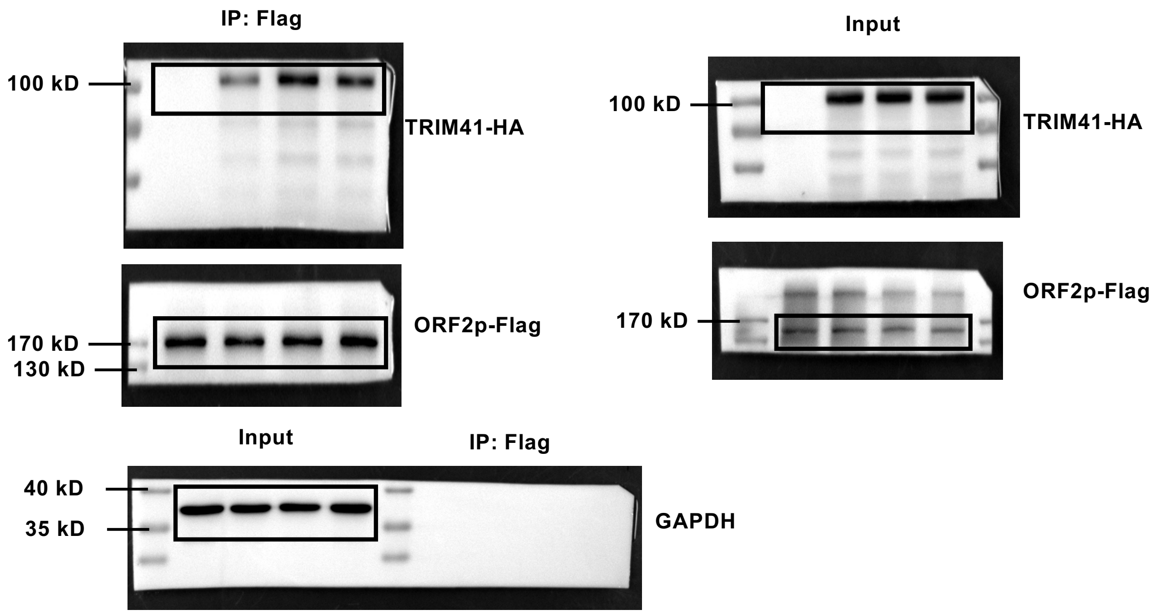

Supplementary Fig. 5g

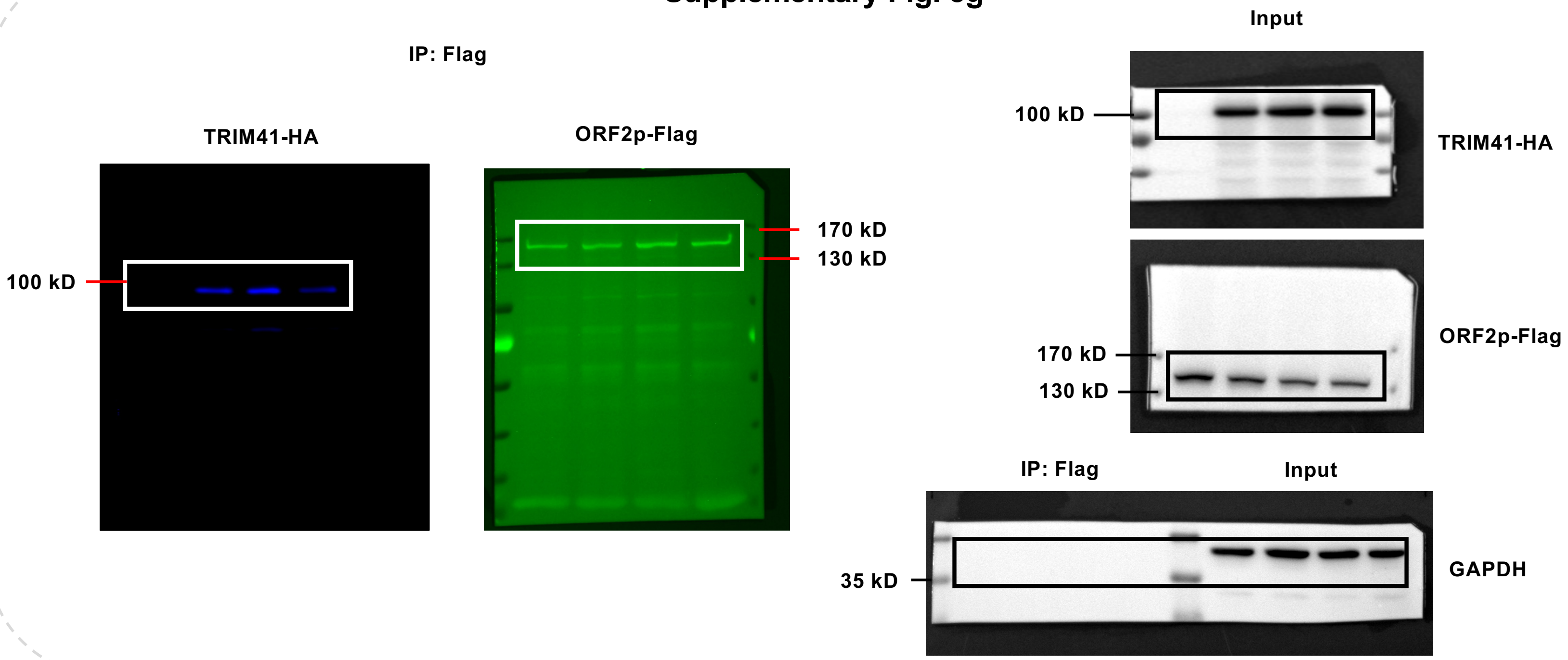

Supplementary Fig. 5i

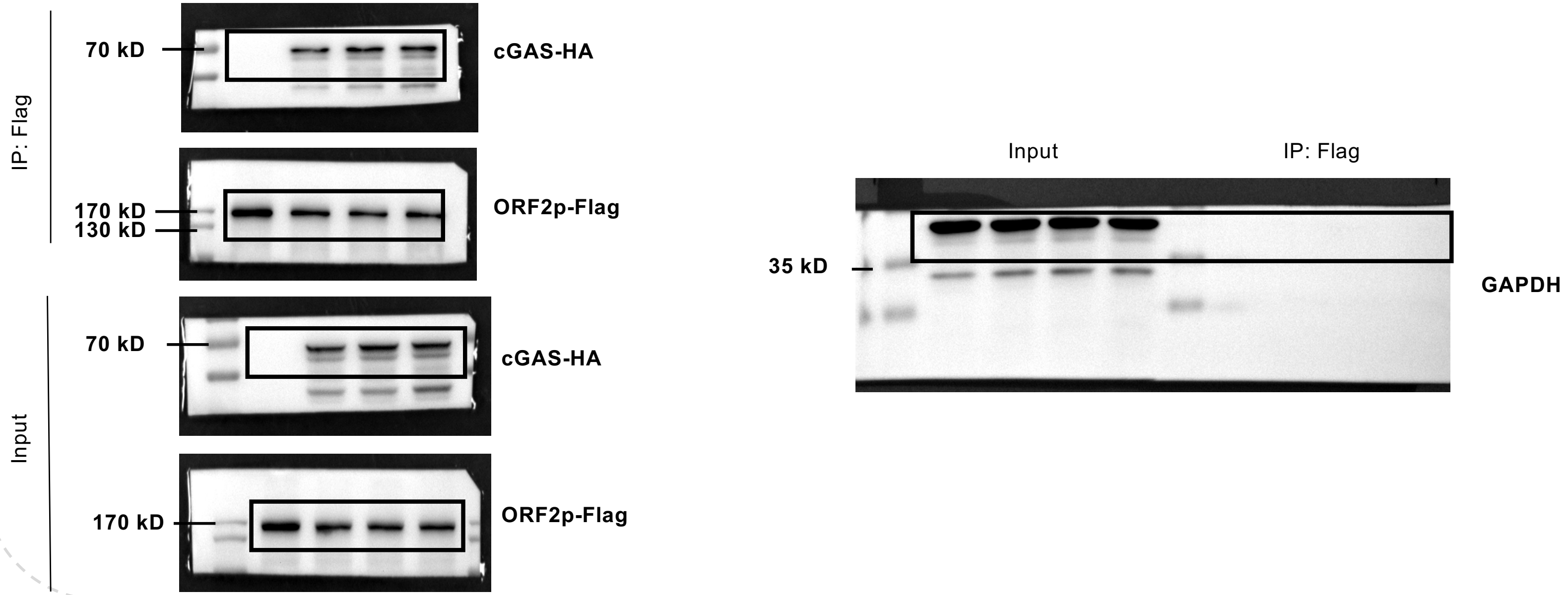

Supplementary Fig. 5j

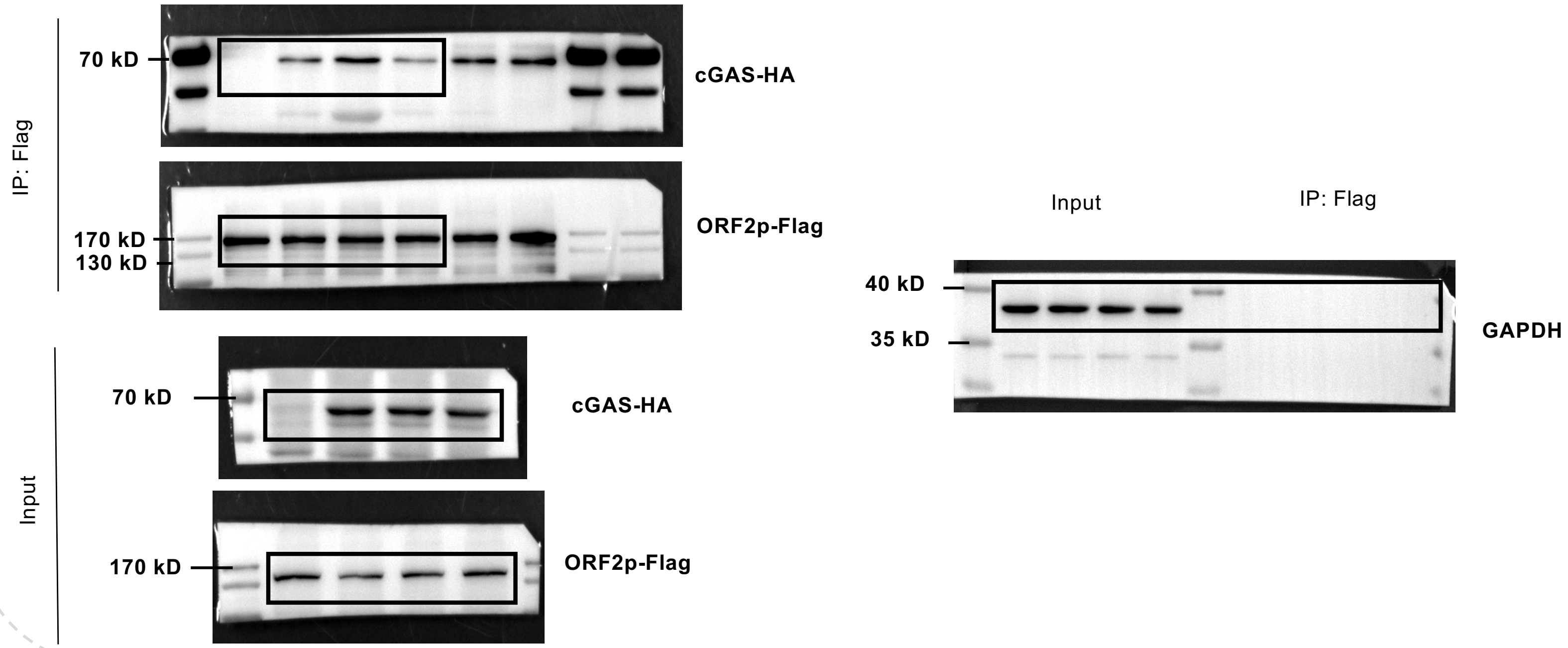

Supplementary Fig. 5k

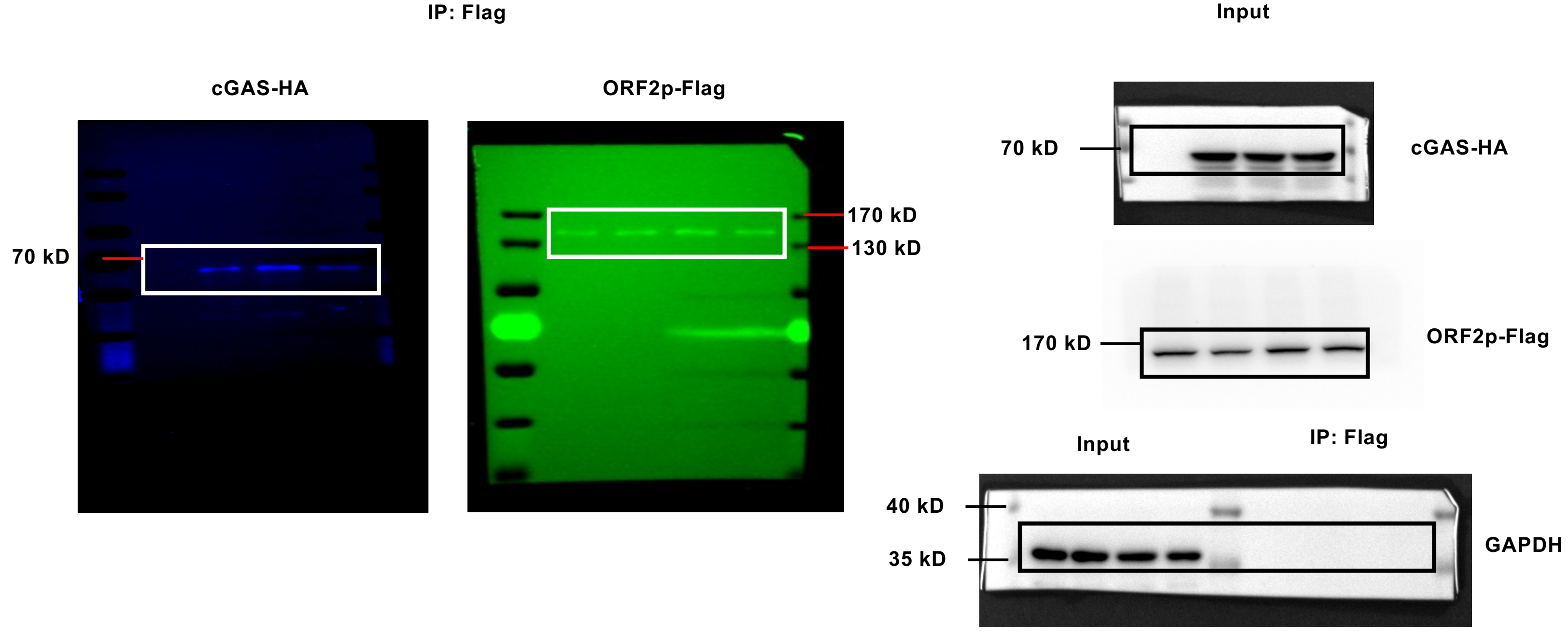

Supplementary Fig. 6a

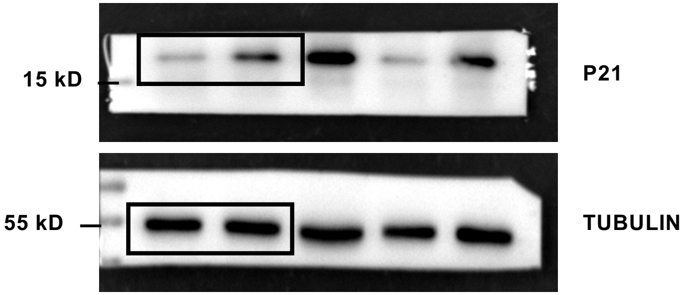

Supplementary Fig. 7d

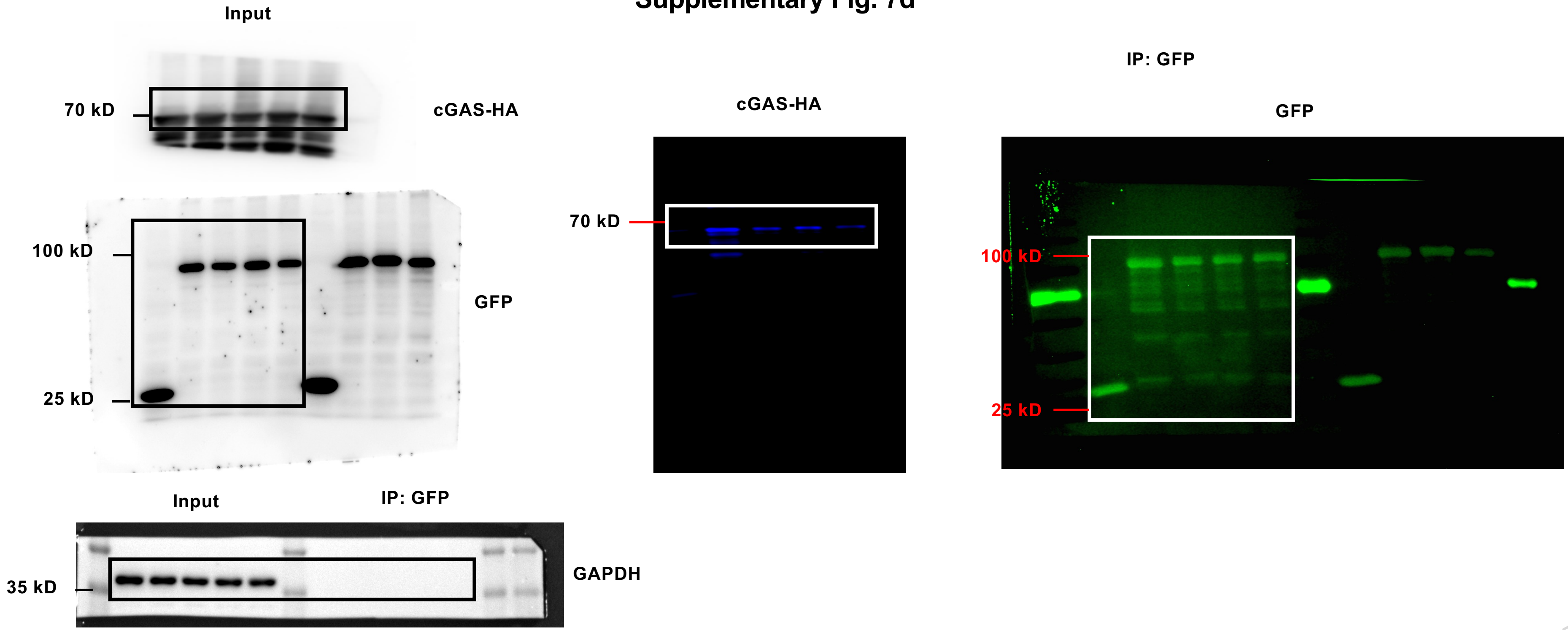

Supplementary Fig. 7e

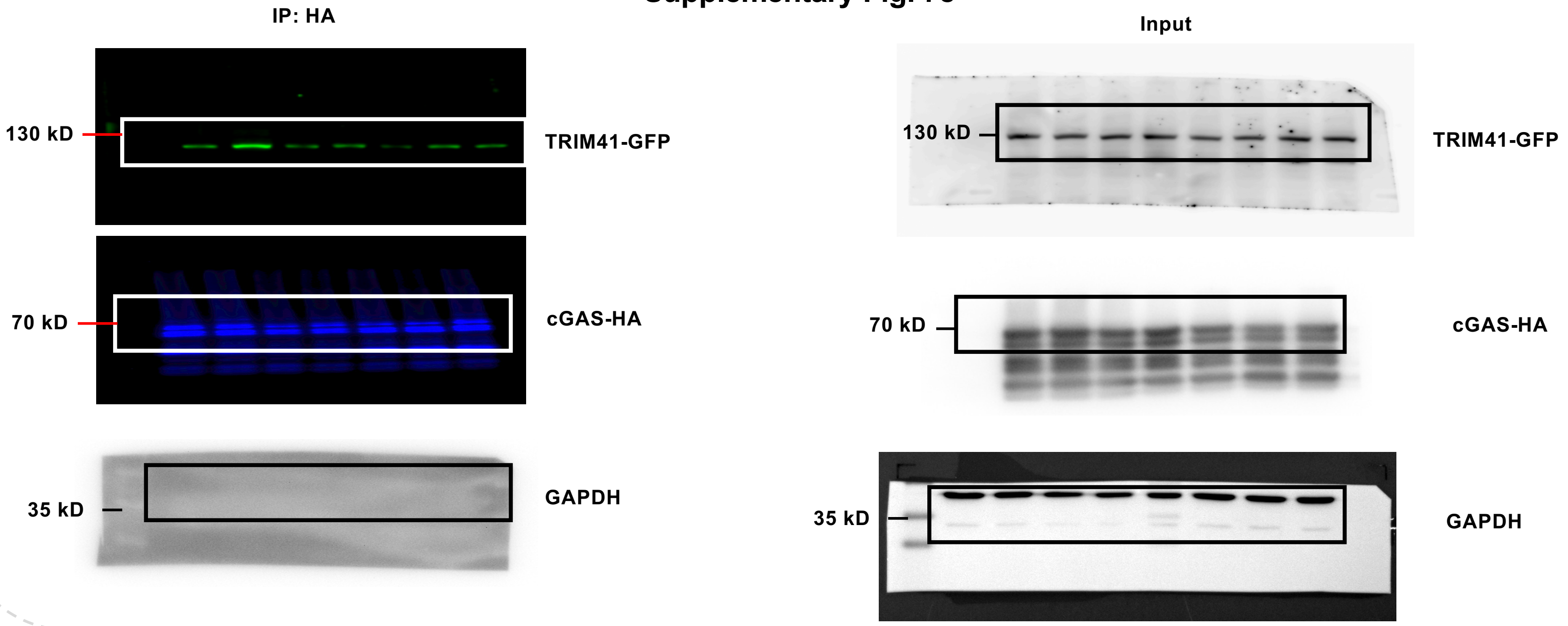

Supplementary Fig. 7f

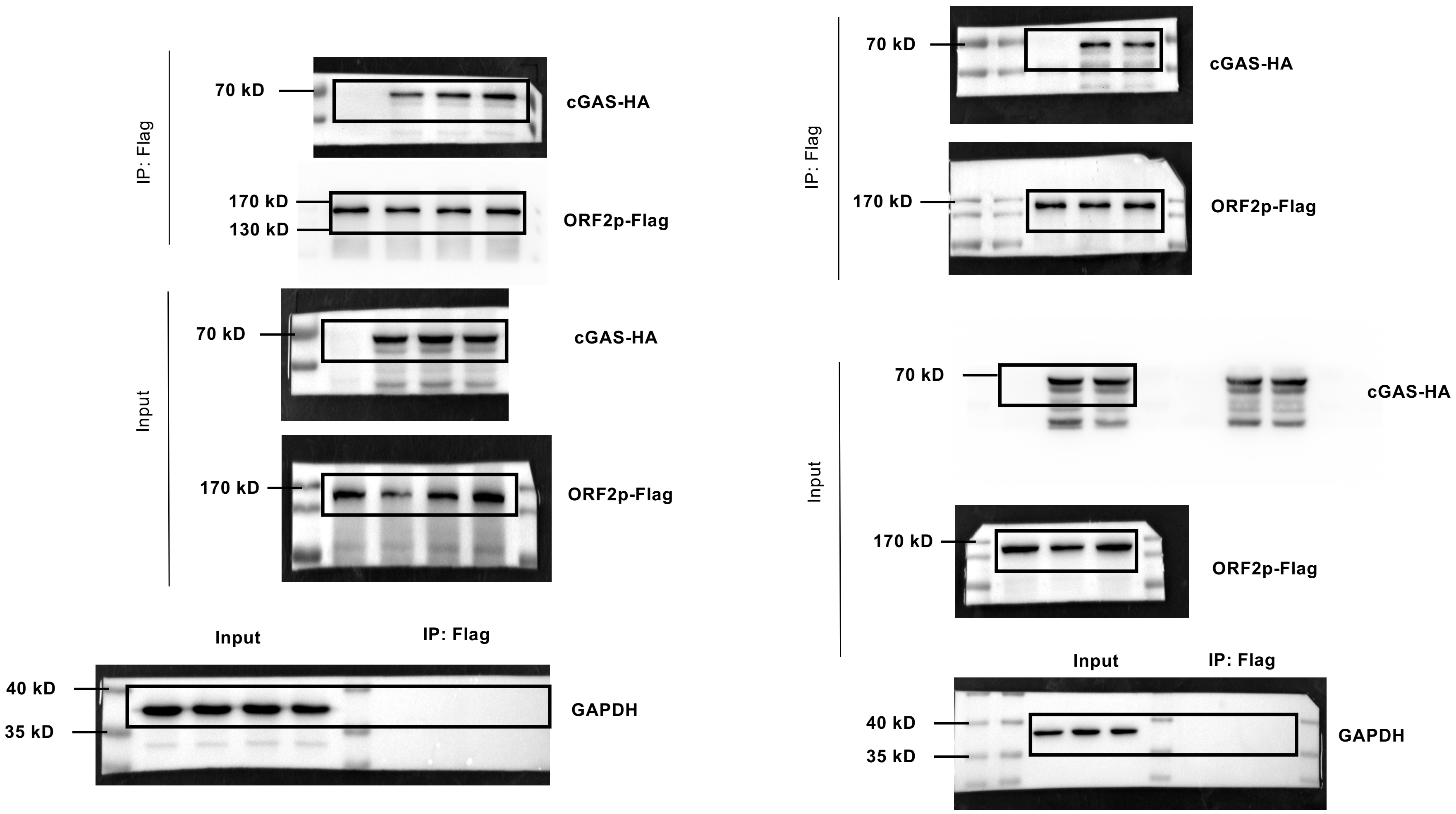

Supplementary Fig. 7g

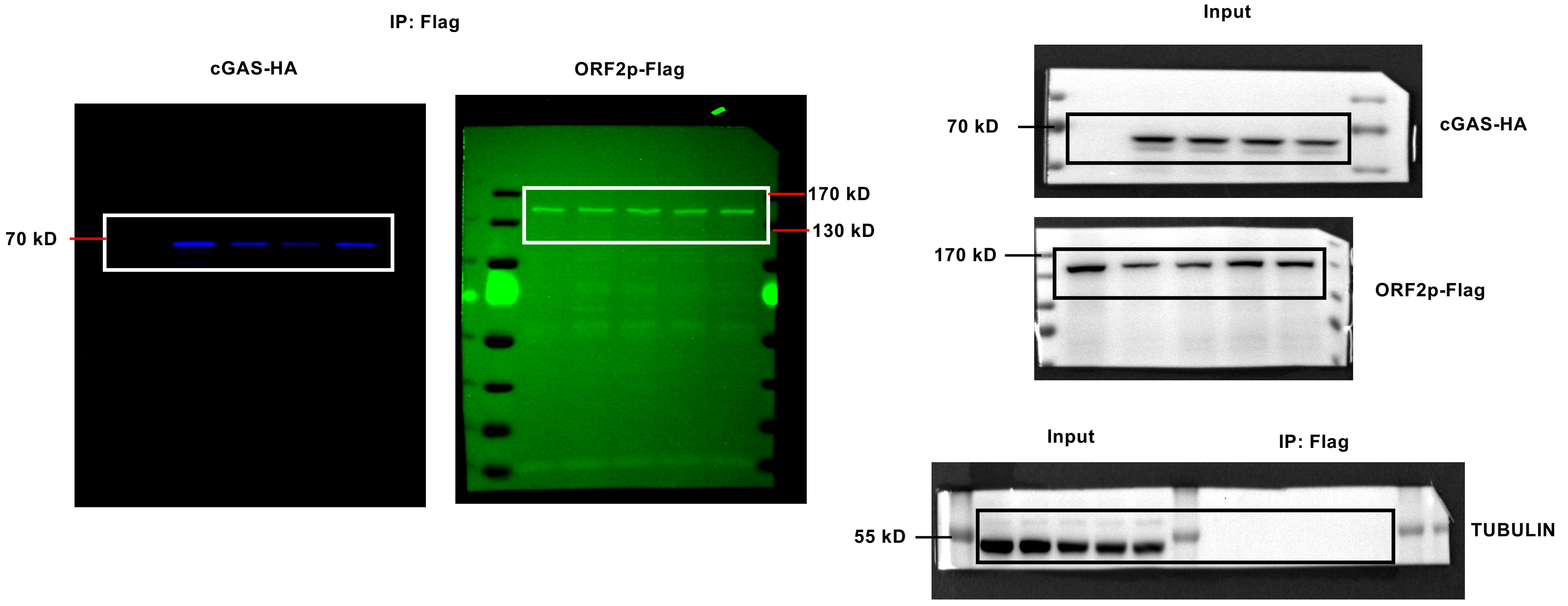

Supplementary Fig. 7h

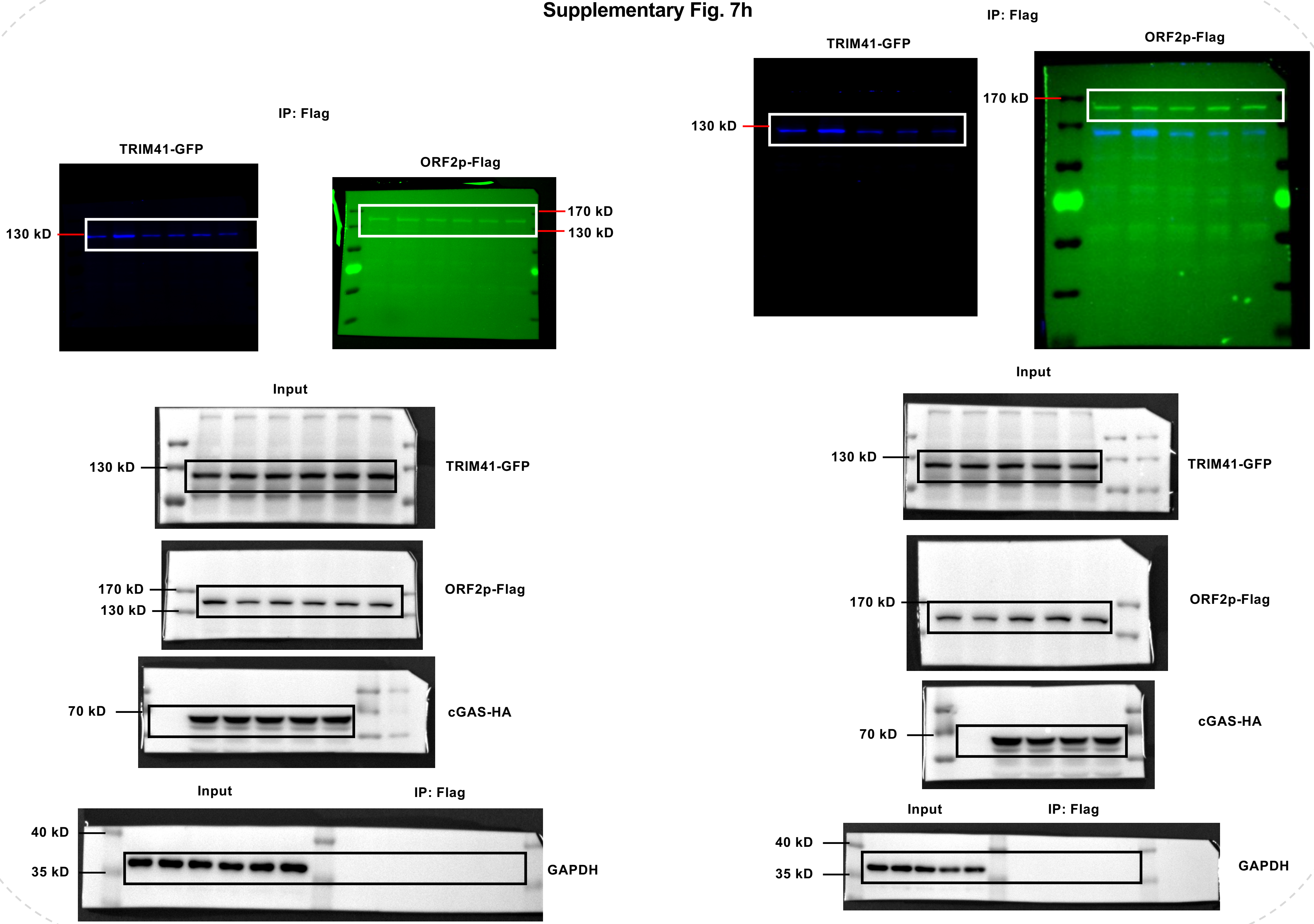

Supplement: Supplementary file 4 — Source Data [file 41467_2023_43001_MOESM4_ESM.zip › Source data/Mao source westerns.pdf]
